# Cytokine‐Engineered Chimeric Antigen Receptor‐T Cell Therapy: How to Balance the Efficacy and Toxicity

**DOI:** 10.1002/advs.202518547

**Published:** 2026-02-03

**Authors:** Xinru Zhang, Gulizeba Aimaiti, Yuanye Guan, Yuzhe Sha, Wei Zhou, Jiefeng Shen, Bo Zhao, Wei‐En Yuan

**Affiliations:** ^1^ Engineering Research Center of Cell & Therapeutic Antibody Ministry of Education and School of Pharmacy Shanghai Jiao Tong University Shanghai China; ^2^ Shanghai Frontiers Science Center of Drug Target Identification and Delivery School of Pharmaceutical Sciences Shanghai Jiao Tong University Shanghai China; ^3^ National Key Laboratory of Innovative Immunotherapy Shanghai Jiao Tong University Shanghai China; ^4^ College of Chemistry and Chemical Engineering Donghua University Shanghai China

**Keywords:** cancer therapy, CAR engineering, CAR‐T cell, cytokines, CRS

## Abstract

Chimeric antigen receptor (CAR)‐T cell immunotherapy has revolutionized the paradigm in hematological malignancies. However, its efficacy in treating solid tumors remains limited because the immunosuppressive tumor microenvironment (ITME) seriously blocks T cell activation, infiltration, and proliferation. Cytokines, driving potent assisted function by enhanced T cell expansion, persistence, and direct tumor cell killing, have long been acknowledged as promising candidates combined with CAR‐T cells to improve treatment outcomes. Despite their preclinical success, significant toxicity occurs in up to one‐third of patients induced by powerful immune‐mediated cytokine release syndrome (CRS) and immune effector cell‐associated neurotoxicity syndrome (ICANS). In these cases, the risk‐benefit unbalance is less advantageous for advanced cancer therapies, appealing for a profound understanding of pathophysiological mechanisms of CRS and ICANS, as well as improved management of regulating cytokine production. In this review, we first provide an overview of activation and cytotoxic mechanisms of CAR‐T cells. Second, obstacles to CAR‐T cells in the ITME are introduced in detail. Third, the advanced design of CAR‐T engineered cytokines, coupled with current research progress, is described. Furthermore, pathophysiology and clinical features of CRS and ICANS are described in detail. Lastly, prevention and/or intervention approaches of the two above‐mentioned toxicities are emphasized both for developing novel therapeutics and maximizing the benefit of patients.

## Graphical abstract

Cytokine‐engineered CAR‐T cells represent a promising immunotherapy against malignancies due to direct tumor killing and potent immunity response. However, significant toxicities, including CRS and ICANS, have restricted clinical applications. How to keep the risk‐benefit balance of the advanced therapy is of great importance for maximizing the benefit of patients?

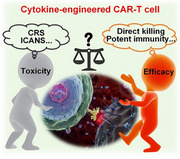

**Abbreviations**
ADCC: antibody‐dependent cellular cytotoxicity
ALP: alkaline phosphatase
ALT: alanine aminotransferase
ANP: atrial natriuretic peptide
AP‐1: activator protein 1
AST: aspartate aminotransferase
ASTCT: American Society for Transplantation and Cellular Therapy
ATP: adenosine triphosphate
B‐ALL: B cell acute lymphoblastic leukemia
BBB: blood‐brain barrier
CAFs: cancer‐associated fibroblasts
CAR: chimeric antigen receptor
cDCs: conventional dendritic cells
CEA: carcinoembryonic antigen
CMV: cytomegalovirus
CRS: cytokine release syndrome
CTL: cytotoxic T lymphocyte
CTLA‐4: cytotoxic T‐lymphocyte‐associated antigen 4
CXCL12: C‐X‐C motif chemokine ligand 12
DAMPs: damage‐associated molecular patterns
DBCO: dibenzocyclooctyl
DIC: disseminated intravascular coagulation
ECD: extracellular domain
ECM: extracellular matrix
EGFR: epidermal growth factor receptor
ERK: extracellular signal‐regulated kinase
Fas‐FasL: Fas‐Fas ligand
GM‐CSF: granulocyte‐macrophage colony stimulating factor
GvHD: graft‐versus‐host disease
HDAd: helper‐dependent adenovirus
HL: Hodgkin lymphoma
HMGB1: high‐mobility‐group protein B1
HSP90: heat‐shock protein 90
HSPGs: heparin sulfate proteoglycans
HSV‐TK: herpes simplex virus tyrosine kinase
ICAM‐1: intercellular adhesion molecule 1
ICAM‐2: intercellular adhesion molecule 2
ICANS: immune effector cell‐associated neurotoxicity syndrome
iCAR: inhibitory counterpart
iCasp9: inducible caspase 9
IFN: interferon
IL‐2: interleukin‐2
IS: immunological synapse
ITAMs: immunoreceptor tyrosine‐based activation motifs
ITEM: immunosuppressive tumor microenvironment
LAT: linker for activation of T cells
LID: ligand‐induced degradation
LINC: linker of nucleoskeleton and cytoskeleton
LOX: lysyl oxidase
mAbs: monoclonal antibodies
MDSCs: myeloid‐derived suppressor cells
MHC: major histocompatibility complex
MMP: matrix metalloproteinases
MSCs: mesenchymal stromal cells
MSCV: murine stem cell virus
MSLN: mesothelin
MTR: metyrosine
MT‐SP1: membrane‐type serine protease 1
NFAT: nuclear translocation of nuclear factor of activated T cells
NF‐κB: nuclear factor‐κB
NHL: non‐Hodgkin lymphoma
NK cells: natural killer cells
Oad: oncolytic adenovirus
ODD: oxygen degradation domain
OTOT: on‐target off‐tumor effect
PET: positron emission tomography
PGE2: prostaglandin E2
PLC‐γ: phospholipase C‐γ1
PSCA: prostate stem cell antigen
RCC: renal cell carcinoma
RECIST: response evaluation criteria in solid tumors
ROS: reactive oxygen species
scFv: single‐chain variable fragment
scRNA‐seq: single cell RNA sequencing
SH2: src homology 2
SLO: secondary lymphoid organ
SLP‐76: SH2 domain‐containing leukocyte protein of 76 kDa
SUPRA CAR‐T cell: split universal and programmable CAR‐T cell
synNotch: synthetic Notch
TAMs: tumor‐associated macrophages
tBID: truncated BH3‐interacting domain death agonist
TCR: T cell receptor
TGF‐β: transforming growth factor‐β
TH: tyrosine hydroxylase
Th1: type 1 T helper
TILs: tumor‐infiltrating lymphocytes
TMD: transmembrane domain
TNF: tumor necrosis factor
Tregs: CD4+Foxp3+ regulatory T cells
uPA: urokinase‐type plasminogen activator
VCAM‐1: vascular cell adhesion molecule 1
VEGF: vascular endothelial growth factor
VLA4: very late antigen‐4
vWF: von Willebrand factor
ZAP70: zeta‐chain‐associated protein kinase 70

## 1 Introduction

Based on the genetic reprogramming of T cells with chimeric antigen receptors (CARs), CAR‐T cells are endowed with the ability to directly identify and kill malignancies expressing corresponding antigens, as well as durable immunologic memory to suppress cancer recurrence [1]. Currently, there are six CAR‐T products approved by the FDA for blood cancer treatment, desiring to perpetuate glory after great success in CD19‐expressing refractory and relapsed B cell malignancies [2]. CARs are synthetic antigen recognition receptors, which are composed of an extracellular domain, a hinge region, a transmembrane domain (TMD), and an intracellular signaling domain. Extracellular domain is a single‐chain variable fragment (scFv) to recognize tumor‐specific antigens, and is further linked with TMD through the hinge region. Intracellular signaling domain, composed of the individual CD3ζ signaling domain initially and developing to include CD28 and 4‐1BB costimulatory domains recently, promotes CAR‐T cells proliferation and cytokines secretion in vivo once activated by antigen‐expressing target cells. However, serious obstacles still discourage CAR‐T cell‐based immunotherapies from application for solid tumors because of their dense tumor structure and complicated immunosuppressive tumor microenvironment (ITME), thus limiting T cell infiltration and promoting T cell exhaustion [3].

The ITME constitutes the principal impediment to CAR‐T cell therapy for solid tumors. The capacity of CAR‐T cells to efficiently migrate to the tumor site, penetrate inhibitory barriers, and persist within the hostile tumor microenvironment constitutes a critical prerequisite for exerting their antitumor function. However, there are three obstacles encountered by CAR‐T cells in this exhausting ITME: (A) “Integrate physical obstacles,” (B) “Mechanical constraints,” and (C) “Biochemical suppression.” Solid tumors are encased within a stiff physical barrier comprising the extracellular matrix (ECM), in contrast to hematological malignancies, which are recognized as the first physical barrier. As tumor progression advances, collagen deposition and cross‐linking within the ECM intensify, leading to a progressive increase in ECM stiffness. Concurrently, elevated interstitial pressure resulting from hypoxia and acidic shifts within the tumor microenvironment further elevates ECM rigidity [4]. This barrier significantly impedes CAR‐T cell infiltration into tumor tissues, thereby compromising the efficacy of CAR‐T cell therapy against solid tumors. In addition to stiff physical barriers, self‐mechanical limitations arise from CAR‐T cells' inherent properties, such as reduced deformability and motility under high ECM stiffness. These limitations hinder their ability to penetrate dense tumor matrices. Notably, nuclear DNA damage within CAR‐T cells predominantly occurs during this stage, potentially impairing their immune effector functions [5]. Moreover, the altered ECM composition, enriched with inhibitory molecules, creates a hostile milieu that suppresses CAR‐T cell activation and proliferation. Research has demonstrated that multiple molecules, constituting an “invisible immunosuppressive barrier,” populate the ECM within solid tumors. This barrier is characterized by the presence of immunosuppressive cells, such as macrophages and regulatory T cells, alongside immunosuppressive factors, including CXCL12 and TGF‐β. Collectively, these elements significantly impair the intensity of the immune response elicited by CAR‐T cells. These barriers necessitate innovative strategies to enhance CAR‐T cell efficacy. Modulating ECM stiffness or targeting immunosuppressive molecules could mitigate these adverse effects. Additionally, engineering CAR‐T cells to resist DNA damage and promoting their adaptability to diverse ECM environments might bolster their tumor‐penetrating capabilities, thereby improving overall therapeutic outcomes [6].

Cytokines are essentially small glycoproteins and polypeptides that execute functions via an autocrine or paracrine pathway. Through mediating interactions between immune and other cells, cytokines are able of inducing inflammations to promote or inhibit tumor cell growth. There are several classical cytokines (such as interleukin‐2 (IL‐2), IL‐12, IL‐15, IL‐18, and interferon (IFN)) that augment T cell priming, activation, and infiltration for further stimulating adaptive and innate immunity, and have possessed antitumor activity in preclinical trials [7]. For instance, both IL‐12 and IL‐18 accelerate the differentiation of CD4 T cells to Th1 cells, then Th1 cells secrete other cytokines such as IL‐2 and IFN‐γ to promote antitumor response. Activated by cytokines, including IL‐2, IL‐12, IL‐18, and IFN‐γ, natural killer (NK) cells can also trigger innate immunity to kill tumor cells [8]. Sharing sufficient effects conducted in patients with renal cell carcinoma (RCC), metastatic melanoma, follicular lymphoma, and hairy cell leukemia, IL‐2 and IFN‐α have already been approved by the FDA as monotherapy in clinical treatment [9]. Meanwhile, the cytokine antitumor activity seen in preclinical models led to the study of cytokines in clinical trials, with GM‐CSF, IL‐2, and IFN‐α being among the first studied. The promising immune‐amplifying potential of cytokines has ignited interest in combining them with other therapies, including genetic editing, biological recombination, as well as immune therapy, to combat complex malignancies collaboratively [10, 11]. Composite system‐engineered cytokines and CAR‐T cells may prove a shift in the field of cancer therapeutics. However, significant toxicities occur that are directly associated with the powerful immune induction, such as cytokine release syndrome (CRS) and immune effector cell‐associated neurotoxicity syndrome (ICANS). Therefore, keeping the balance between cytokines and CAR‐T cells to optimize T cell activation, proliferation, and infiltration, as well as circumvent CRS and ICANS are of great importance for future research. This review provides an overview of the activation and tumor‐killing mechanisms of CAR‐T cells. Second, the obstacles of CAR‐T cells in the ITME are introduced delicately. The advanced design of CAR‐T engineered cytokines, coupled with current research progress, is described in the third section. Furthermore, pathophysiology and clinical features of CRS and ICANS are described. Lastly, prevention and/or intervention approaches of the two above‐mentioned toxicities are emphasized both for developing novel CAR‐T cell therapeutics and maximizing benefiting patients.

## 2 Structure of CAR Molecule and Signaling Involved in CAR‐T Cell Activation

**FIGURE 1:**
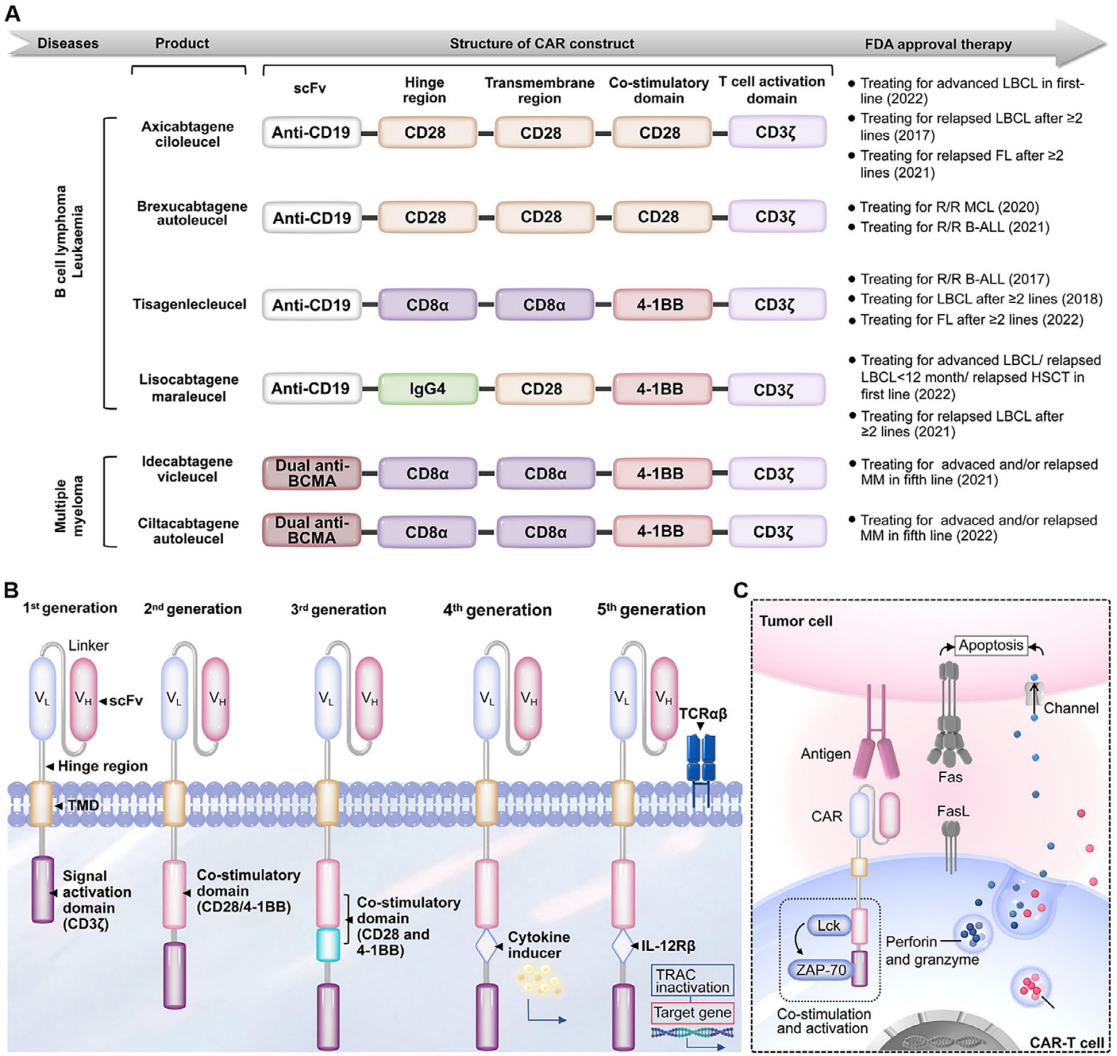
Clinical development, structures, and activation signaling of CAR‐T cells. (A) A total of six CAR products are currently available commercially, including four for patients with B‐cell lymphomas, two for patients with B‐ALL, and two for those with MM. All approved products have a second‐generation CAR construct. (B) Structure of different generations of CAR molecules. (C) CAR‐T cell signaling and targeted killing when engaged with tumor antigens.

CAR‐T cells represent engineered T lymphocytes incorporating a synthetic receptor that combines antigen‐binding capability with T cell activation functionality. Presently, six CAR‐T cell products featuring CD28 or 4‐1BB costimulatory domains have received FDA approval for treating hematological malignancies and multiple myeloma (Figure 1A). The molecular design of CAR confers the capacity for specific tumor cell recognition and major histocompatibility complex (MHC)‐unrestricted cytolytic activity. CAR‐T technology has evolved through fifth generations, with constructs comprising four principal domains: an extracellular tumor antigen‐recognition domain, a hinge region, a TMD, and an intracellular signaling domain (Figure 1B) [12]. Remarkably, the disparity across generations is mainly characterized by the enhancement of intracellular structures and the incorporation of cytokines.

### 2.1 Extracellular Tumor Antigen‐Recognition Domain

The antigen recognition domain constitutes the molecular foundation for CAR‐specific binding to tumor antigens, predominantly utilizing scFv. This structure is typically engineered by connecting the variable light chain (V_L_) and variable heavy chain (V_H_) domains of monoclonal antibodies via antigen‐specific peptide linkers. Theoretically, the V_L_‐linker‐V_H_ configuration more closely approximates the natural antibody conformation. Both V_L_‐linker‐V_H_ and V_H_‐linker‐V_H_ orientations demonstrate comparable antigen recognition performance. The linker sequences‐(Gly‐Gly‐Gly‐Gly‐Gly‐Ser)_3_‐ and ‐(Gly‐Gly‐Gly‐Gly‐Ser)‐ are the most commonly employed, as they effectively connect V_H_ and V_L_ domains while preserving structural flexibility [13]. This flexibility permits the V_H_ and V_L_ complementary determining regions (CDRs) to pair correctly, forming a monovalent antigen‐binding site. The scFv fragment is inherently monovalent. A structure comprising two heavy chain variable regions and two light chain variable regions within a single polypeptide chain is designated a tandem di‐scFv. Reducing the linker peptide length from approximately 15 amino acids to 3–12 amino acids facilitates the pairing of V_H_ and V_L_ domains derived from two distinct scFv molecules, forming a dimeric structure known as a bispecific T‐cell engager (a bispecific antibody composed of variable regions targeting two different antigens). Similarly, a tandem tri‐scFv antibody, incorporating three heavy chain and three light chain variable regions, can be engineered. Further shortening the linker peptide enables the pairing of V_H_ and V_L_ domains from three different molecules, resulting in the formation of a triabody. These scFv oligomers exhibit higher antigen‐binding valency and affinity compared to monomeric scFv fragments [14]. scFv‐based constructs offer several advantages: First, the absence of an Fc fragment minimizes Fc receptor (FcR)‐mediated binding to immune cells. Second, the shorter amino acid sequence reduces immunogenicity and host rejection of heterologous antibodies. Third, their smaller molecular weight enhances tumor tissue penetration. Finally, the generation of bispecific antibodies avoids the requirement for cell hybridization and chemical crosslinkers, thereby improving safety and reducing immunogenicity. Collectively, the integration of scFv fragments confers or augments the antigen recognition and binding capacity of CARs. This strategy liberates T cells from MHC restriction, thereby mitigating tumor immune evasion, and additionally enables immune cells to recognize non‐peptide antigens [15]. Fine‐tuning CAR affinity can further reduce on‐target/off‐tumor toxicity against normal tissues expressing low antigen levels while maintaining sufficient effector function to eliminate antigen‐overexpressing malignant cells. Another significant advantage of scFv‐based CARs is their MHC‐independent antigen recognition, which overcomes tumor escape mechanisms involving MHC downregulation and permits targeting of non‐peptide antigens, such as glycolipids or tumor‐specific glycosylation patterns. However, scFv‐based CARs also present distinct limitations. First, due to the non‐exclusive expression of target antigens on tumor cells, “on‐target off‐tumor” toxicity can occur, leading to the elimination of normal cells expressing the antigen. Second, scFvs exhibit relatively poor stability and are susceptible to denaturation by environmental factors such as temperature and pH fluctuations. Consequently, variations in manufacturing processes or the in vivo milieu may compromise scFv integrity. Additionally, the V_H_ and V_L_ domains of distinct scFvs are prone to mispairing, potentially resulting in loss of target antigen binding specificity and increased immunogenicity of the CAR construct [16, 17].

### 2.2 Hinge Region

The hinge domain serves to connect the antigen recognition domain with the transmembrane domain. The origin, length, flexibility, and composition of this domain significantly influence CAR antitumor activity and associated adverse effects. The optimal hinge domain length is contingent upon the location and accessibility of the target antigen and its specific epitopes. CAR‐T cells exhibit enhanced activation when the target epitope resides proximal to the target cell membrane. Modulation of hinge region length facilitates maintenance of the optimal spatial distance between CAR‐T cells and target cells, thereby promoting immune synapse formation and preventing attenuation of CAR signaling. Studies have demonstrated that shorter hinge domains confer superior activation potential for CAR‐T cells targeting CD19, carcinoembryonic antigen, and IL‐13 receptor α2 compared to longer hinges. Conversely, for targets including ROR1, MUC1, NCAM, and 5T4, an extended hinge domain is essential to overcome spatial hindrance and enable effective contact with the target antigen. Consequently, the optimal hinge length varies substantially depending on the specific epitope targeted. The structural origin of the hinge domain warrants careful consideration. Current hinge designs predominantly derive from IgG hinge regions or the extracellular domains of CD8α or CD28. IgG‐based hinges typically incorporate the CH_2_–CH_3_ domains, primarily sourced from IgG1 or IgG4 subclasses [18]. While offering considerable structural flexibility, a significant disadvantage of IgG‐derived hinges, observed in clinical studies, is their association with poor CAR‐T cell persistence [19]. This limitation potentially stems from the presence of amino acid motifs within the CH_2_ domain capable of binding Fcγ receptors (FcγRs) expressed on innate immune cells, including monocytes/macrophages, dendritic cells (DCs), neutrophils, and NK cells [20]. Such Fc‐FcγR interactions can trigger non‐productive innate immune activation, encompassing antibody‐dependent cell‐mediated cytotoxicity (ADCC) and phagocytosis, ultimately contributing to CAR‐T cell exhaustion. Furthermore, Fc‐FcγR engagement may induce ligand‐independent tonic signaling, leading to activation‐induced T cell death (AICD). Current research strategies often mitigate these detrimental effects through structural modifications of IgG‐derived hinges, such as substituting IgG1‐CH_2_ framework residues with corresponding IgG2 amino acids or deleting the CH_2_ domain outright.

### 2.3 Transmembrane Domain

As a structural bridge connecting extracellular and intracellular compartments, the TMD, typically derived from specific transmembrane receptor proteins, exerts a significant influence on the timely and stable exchange of information between intracellular and extracellular domains. Clinically prevalent TMD sources include CD4, CD8α, CD28, and CD3ζ. Researchers have analyzed the regulatory effects of hinge domains and TMDs on CAR expression levels and cytotoxicity. Results demonstrated that CAR constructs co‐modified with hinge region/transmembrane domain exhibited stronger regulatory effects than those modified solely with the hinge region. This indicates that the TMD significantly impacts CAR expression stability and functionality. TMD‐mediated CAR dimerization and interactions with endogenous proteins facilitate the formation of dimers or trimers, ultimately potentiating T cell activation. Multiple studies confirm that TMDs (including FcεRIγ, CD3ζ, CD28, CD16, NKp44, NKp46, NKG2D, DNAM‐1/2, and 4‐1BB) promote signal transduction and T cell activation through CAR dimerization [21]. Compared to CD8α‐derived TMD, the CD28 TMD demonstrates a greater propensity for dimerization, thereby reducing the antigen density threshold required for T cell activation. Furthermore, TMD composition influences cytokine secretion profiles in CAR‐engineered cells. Studies have demonstrated that CARs incorporating 86‐amino acid TMDs not only elicit potent antitumor responses but also exhibit favorable safety profiles. Strategic modification of the CAR TMD can effectively modulate cytokine secretion and ameliorate CAR‐T cell toxicity [22].

### 2.4 Intracellular Signaling Domain

The continuous optimization of the CAR structure is critically reflected in the design of the co‐stimulatory domain. Complete T cell activation necessitates at least two distinct stimulatory signals. The primary activation signal is delivered by the engagement of MHC molecules presenting specific antigenic peptides on antigen‐presenting cells (APCs) with the TCR. A second critical activation signal typically arises from the binding of costimulatory receptors on the T cell surface to their cognate ligands on APCs. Incomplete antigenic stimulation can induce T cell anergy. The first generation of CARs comprised solely an antigen recognition domain fused to signaling domains derived from FcγR or CD3ζ. While this minimal structure demonstrated cytotoxicity against target cells both in vitro and in vivo, it exhibited limited antitumor efficacy and poor persistence in clinical trials [2]. To enhance T cell proliferation and sustained survival, researchers engineered second‐generation CARs by incorporating costimulatory domains (CD28 or 4‐1BB) to provide the requisite “second signal” for T cell activation. Although CD28 as a costimulatory domain enhances T cell cytotoxic capacity, it fails to significantly improve sustained T cell persistence [23]. Conversely, 4‐1BB (TNFRSF9, CD137, ILA), predominantly expressed on activated T cells, stimulates T cells by activating downstream pathways involving NF‐κB, c‐Jun, and p38. Unlike CD28, 4‐1BB enhances T cell activity primarily by stimulating the proliferation, cytokine release, and cytolytic activity of effector T cells (rather than naive T cells), while concurrently inhibiting AICD. However, the cytotoxic potency mediated by 4‐1BB costimulation remains relatively constrained. To augment antitumor efficacy and prolong in vivo persistence, third CARs were developed by combining two distinct costimulatory domains [24]. Commonly employed costimulatory domains in CAR design originate from either the immunoglobulin superfamily (e.g., CD28, ICOS) or the tumor necrosis factor receptor superfamily (TNFRSF) (e.g., 4‐1BB, OX40, CD27). Studies indicate that CAR‐T cells incorporating CD28‐OX40 exhibit superior in vitro expansion and cytotoxicity compared to those lacking OX40 [25]. Regrettably, the clinical performance of third‐generation CAR‐T cells has not proven superior to second‐generation constructs. This observation suggests that merely increasing the number of integrated costimulatory domains does not necessarily enhance CAR‐mediated immune cell activation.

To integrate immune checkpoint blockade and counteract the tumor immunosuppressive microenvironment, fourth‐generation CARs (also termed TRUCKs‐T cells redirected for universal cytokine‐mediated killing) were engineered. These augment second‐generation CARs with the capacity for inducible expression of specific cytokines (e.g., IL‐12, IL‐15, IL‐18). This modification promotes the infiltration of NK cells and macrophages at the tumor site, thereby amplifying the antitumor response [26]. Furthermore, addressing CAR‐T cell controllability concerns, some researchers have incorporated inducible suicide genes (e.g., drug‐sensitive cassettes) into fourth‐generation CAR‐T constructs to regulate their in vivo persistence. Alternative strategies propose the design of molecular switch mechanisms for CAR‐T cell activity modulation [27]. Based on dual targeted therapy and the “AND” logic gate principle, other researchers have modified CAR‐T cells to only exert killing effects when recognizing both A and B antigens simultaneously. This type of logic gate can enhance the accuracy of CAR‐T and reduce off‐target toxicity. The specific structure and design concept will be explained in detail in the following chapters. CAR cell therapy typically involves engineering the patient's own cells in vitro and then reintroducing them back into the body, which limits the scale application and clinical translation of CAR cell therapy, especially CAR‐T cells. To this end, researchers have developed the fifth‐generation CAR‐T therapy—Universal CAR‐T (UCAR‐T). This technology separates the extracellular antigen targeting domain from the T cell signaling unit by introducing two “third‐party” systems (BBIR CAR or supra CAR), enabling CAR‐T cells to recognize multiple antigens [28]. In addition, in vitro destruction of TCR genes and HLA class I genes in T cells derived from allogeneic healthy recipients using gene editing techniques (ZFN, TALEN, and CRISPR/Cas9) can eliminate graft‐versus‐host disease (GVHD) [29]. The development of fifth‐generation CAR‐T cells is advancing. The principal distinction lies in the integration of supplementary membrane receptors. Multiple research avenues are currently under investigation, with the incorporation of the IL‐2 receptor signaling pathway to induce antigen‐dependent JAK/STAT pathway activation being among the most promising. This signaling pathway not only sustains CAR‐T cell functionality and promotes memory T cell formation but also reactivates broader immune responses. The safety of the fifth‐generation CAR‐T is still in the early stages of exploration.

The signal activation domain primarily facilitates transduction of T cell activation signals. Currently, CD3ζ represents the most prevalent activation domain source utilized in CAR‐T cells. The CD3ζ activation domain comprises three immunoreceptor tyrosine‐based activation motifs (ITAMs), whose function exhibits high dependency on lymphocyte‐specific protein tyrosine kinase (Lck) activity. While the number and type of ITAMs in T cells can influence signaling processes, research indicates that a single functional ITAM suffices to achieve therapeutic antitumor efficacy. Furthermore, CAR constructs containing a single ITAM demonstrate superior in vivo performance compared to those incorporating multiple ITAMs, as evidenced by restricted T cell differentiation, increased proportions of central memory CAR‐T cells, and enhanced persistence [30, 31]. Beyond CD3ζ, CD3δ, CD3ε, and CD3γ, theoretically constitute alternative structural domains for CARs. Researchers substituted the conventional CD3ζ domain with CD3δ, CD3ε, and CD3γ peptide chains to evaluate T cell activation efficacy [32]. Results demonstrate that employing these alternative peptide chains in CARs reduces CRS incidence, enhances CAR‐T cell persistence in vivo, and improves the safety and efficacy profile of CAR‐T therapy [33].

### 2.5 Signaling Involved in CAR‐T Cell Activation

CAR‐T cell activation and effector function initiation occur upon CAR engagement with specific tumor antigens. The hinge and TM regions provide structural flexibility, facilitating CAR molecule binding to tumor‐surface antigens. This binding event induces phosphorylation of the intracellular CD3ζ ITAMs [34, 35]. Consequently, Zeta‐chain‐associated protein kinase 70 (ZAP70) docks with phosphorylated ITAMs via its dual Src homology 2 (SH2) domains, initiating phosphorylation of downstream signaling molecules. These include linker for activation of T cells (LAT), SH2 domain‐containing leukocyte protein of 76 kDa (SLP‐76), and phospholipase C‐γ1 (PLC‐γ) [36]. These signaling cascades subsequently transmit CAR activation signals to endogenous T cell activation pathways through three principal mechanisms: (1) The calcium (Ca^2+^)‐calcineurin signaling pathway triggers nuclear translocation of nuclear factor of activated T cells (NFAT), thereby augmenting cytokine gene expression; (2) The extracellular signal‐regulated kinase (ERK)‐mitogen‐activated protein kinase (MAPK) pathway activates activator protein 1 (AP‐1), a key transcription factor, enhancing T cell proliferation and survival; (3) The NF‐κB pathway induces nuclear translocation of NF‐κB, promoting T cell functional differentiation and cytokine secretion. second‐ and third‐generation CAR designs incorporate co‐stimulatory domains fused with CD3ζ to stimulate robust CAR‐T cell expansion and activation. Cytotoxic effector molecules, such as perforin and granzymes, play a significant role in tumor killing and are released during CAR‐T cell‐tumor cell contact upon nonclassical immunological synapse (IS) formation [37]. Through this IS‐mediated transient cell–cell contact cycle, CAR‐T cell stimulation via perforin and granzyme additionally triggers the Fas‐Fas ligand (Fas‐FasL) pathway, resulting in cytokine production and synergistic tumor cell killing. Perforin forms pores in the tumor cell membrane in a Ca^2+^‐dependent manner, facilitating granzyme entry and inducing tumor cell apoptosis. Furthermore, engagement of FasL expressed on CAR‐T cells with Fas expressed on tumor cells directly induces tumor cell apoptosis [30, 38]. Furthermore, cytokines, primarily IFN‐γ and tumor necrosis factor (TNF), are generated during CAR‐T cell effector activation. These cytokines amplify T cell anti‐tumoral efficacy by sensitizing the tumor stroma and enhancing T cell infiltration (Figure 1C) [39]. IFN‐γ enhances CAR‐T cell efficiency through two principal mechanisms: (1) recruiting other immune cells, such as macrophages, to function as antigen‐presenting cells for cytotoxic T cell activation; and (2) upregulating MHC‐I expression and accelerating antigen processing on the tumor surface to overcome T cell recognition barriers. Additionally, CAR‐T cell‐mediated tumor control is indirectly regulated by numerous cellular and molecular events, including adhesion molecule interactions. Structurally, adhesion molecules are expressed on both tumor cells and tumor‐infiltrating lymphocytes (TILs), crucially facilitating immunological synapse (IS) formation for precise tumor killing, as well as promoting CAR‐T cell migration and infiltration into the tumor parenchyma. Nevertheless, CAR‐T cell efficacy in solid tumors remains constrained due to downregulated adhesion molecules within the immunosuppressive tumor microenvironment. Evidence indicates that limited CAR‐T cell‐tumor contact primarily results from reduced expression of the adhesion molecules LFA‐1 and CD2 on TILs, coupled with downregulated or lost expression of their corresponding ligands—ICAM‐1 (for LFA‐1) and CD58 (for CD2)—on the tumor cell surface. Furthermore, diminished extracellular magnesium (Mg^2^^+^) levels within the tumor microenvironment can impair CAR‐T cell effector function and cytotoxicity by reversing the affinity state of LFA‐1 [40, 41]. Conversely, ICAM‐1 expression in solid tumors positively influences CAR‐T cell‐mediated tumor control via an IFN‐γ‐dependent mechanism. Increased IFN‐γ secretion elevates ICAM‐1 transcription, thereby strengthening CAR‐T cell‐tumor adhesion, infiltration, and tumor killing.

## 3 The Dilemma of CAR‐T Cells in Solid Tumor Microenvironment

**FIGURE 2:**
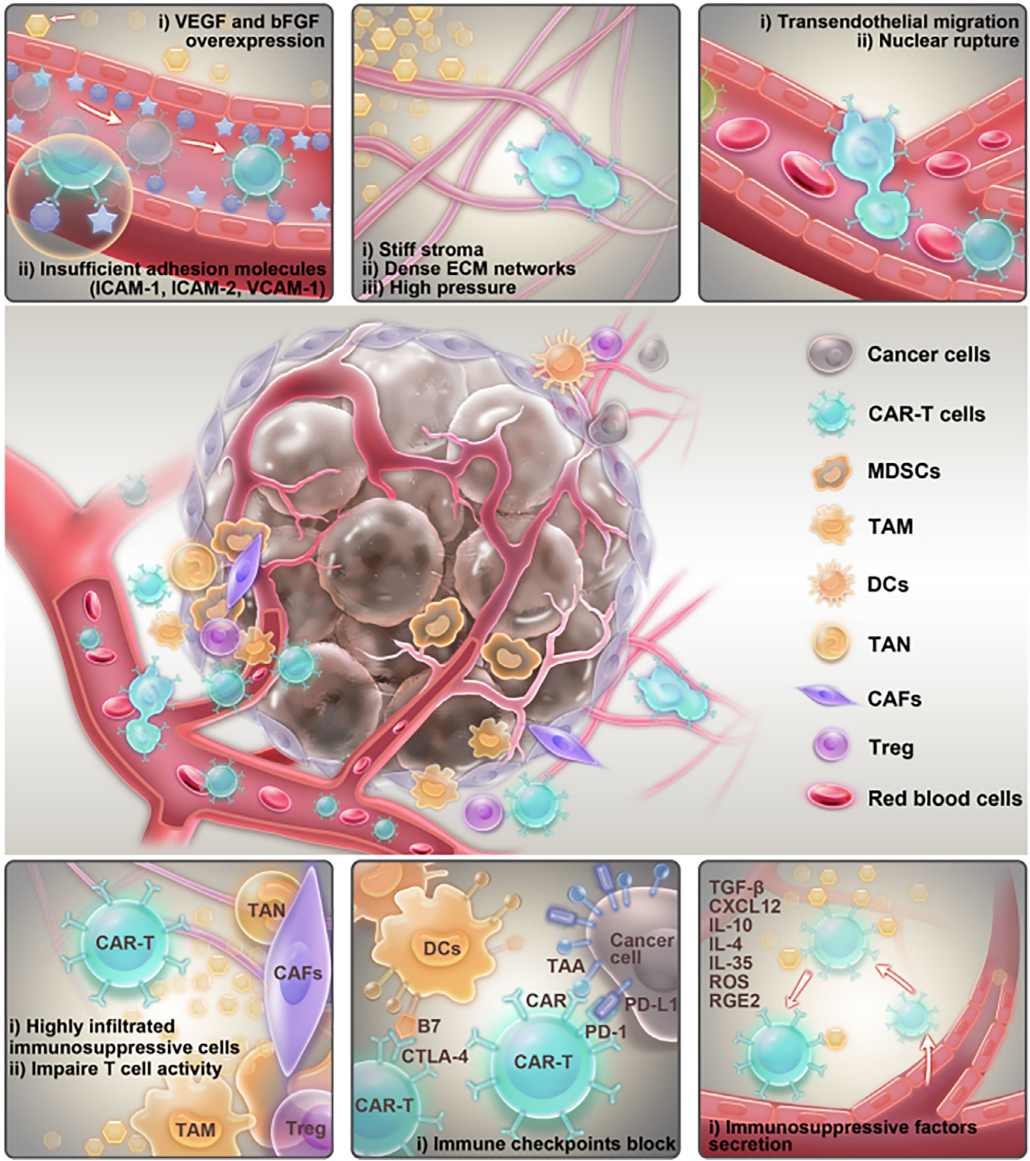
The difficult circumstances of CAR‐T cells in the ITME. During this process, CAR‐T cells adhere to the vascular wall to penetrate endothelial cells and reach the tumor matrix. During the entire process, CAR‐T cells first need to overcome the hard physical barrier formed by the ECM, and second, there may be nuclear rupture during the shuttle process. The last few CAR‐T cells may encounter a large number of immunosuppressive cells or come into contact with cancer cells that express high immune checkpoints, leading to further decline in antitumor function.

The remarkable efficacy of CAR‐T cells in hematologic malignancies has sparked significant interest in harnessing their therapeutic potential against solid tumors. However, CAR‐T cell trafficking and migratory dynamics within solid tumor microenvironments exhibit substantial disparities compared to hematologic malignancies, primarily attributed to the ITME. This pathological milieu results in multiple impediments that compromise therapeutic efficacy, including limited tumor infiltration, restricted cellular trafficking, and modest effector activity. In this section, we are discussing in detail the obstacles encountered by CAR‐T cells in ITME, including three parts as follows: (A) “Integrate physical obstacles,” (B) “Mechanical constraints,” and (C) “Biochemical suppression” (Figure 2). It is of critical importance that promoting preclinical models recapitulate the in vivo ITME to more effectively enhance CAR‐T cell function.

### 3.1 Integrate Physical Obstacles

Prior to arriving at the tumor foci, CAR‐T cells predominantly access the systemic circulation and are transported via the bloodstream to the ITEM. Extravasation is initiated when circulating CAR‐T cells gain sufficient traction and adhesion to counteract receptors expressed on the endothelial walls, which is the first step to entering ITEM. However, the heightened secretion of angiogenic factors (VEGF and bFGF) during tumorigenesis has profoundly diminished the expression of endothelial adhesion molecules, notably intercellular adhesion molecule 1 (ICAM‐1), intercellular adhesion molecule 2 (ICAM‐2), and vascular cell adhesion molecule 1 (VCAM‐1) [42]. This molecular attenuation critically obstructs CAR‐T cell engagement with tumor cells. Although the mechanisms underlying transendothelial migration have been elucidated, the development of a coherent strategy to enhance T cell extravasation remains a significant challenge.

Traversing endothelial junctions and navigating through tumor stroma are basic requirements for CAR‐T reaching tumor cells. There are enormous differences between hematologic malignancies, which can be freely accessed by CAR‐T cells, and solid tumors, which exhibit a complex three‐dimensional structure outside the tumor. The ECM has been widely recognized as a formidable physical barrier that significantly impedes CAR‐T cell infiltration into tumor tissues [43]. A critical hallmark of tumor progression is the progressive stiffening of the ECM, which may be attributed to heightened matrix crosslinking, excessive collagen deposition, and aberrant fiber alignment [44]. These structural modifications collectively regulate cellular migration, proliferation, and apoptotic processes through mechano‐transduction pathways. Collagen deposition and crosslinking represent critical contributors to tissue stiffening, with detailed analysis demonstrating that this process is primarily driven by elevated secretion of lysyl oxidase (LOX) and heightened synthesis of additional extracellular matrix components, including heparin sulfate proteoglycans (HSPGs). Matrix metalloproteinases (MMP) and heparinase enzymes secreted by the tumor itself play a critical role in mediating ECM degradation and reorganization, thereby facilitating primary tumor cell proliferation and migratory capacity [45, 46]. However, this pathway did not appear in the T cells; densely packed and oriented stromal fibers are still great obstacles for CAR‐T infiltration. Additionally, continuous adjustments of ITEM during the tumor growth are characterized as elevated solid stress, interstitial fluid pressure, enzymatic secretion, and cellular contractility onto the ECM, thereby further promoting tighter physical arrangements of collagen fibers as well as stiffening ITME to suppress immune activity and cell migration [47]. An in vitro study revealed that an interstitial fluid pressure exceeding 1 KPa‐simulated through hydrostatic pressure significantly impedes antigen‐specific T cell infiltration into tumor sites. Moreover, growth‐induced solid stress (≥2 kPa), while suppressing cancer cell proliferation, induces blood vessel collapse, exacerbating vascular abnormalities and amplifying interstitial pressure. This reciprocal interaction further elevates total intertumoral pressure, intensifies hypoxia, and augments pH imbalances alongside metabolic waste accumulation [48]. Under hypoxic conditions and TGF‐β infiltration, LOX secretion is upregulated, augmenting ECM stiffness and thereby impeding the engagement between CAR‐T cells and tumor cells. The feedback loop continually contributes to tumor physical barriers responsible for durative tumor development.

### 3.2 Mechanical Constraints

The migratory behavior of T cells within the ITME is a critical focus in immuno‐oncology. Studies indicate that T cells dynamically adapt their migration strategies in response to ECM composition, though the underlying molecular mechanisms remain incompletely understood. Elucidating these migratory properties could enhance the infiltration efficiency of CAR‐T cells into solid tumors. Key determinants of T cell migration include substrate binding‐site density, proteolytic activity, actin‐mediated contractility, microtubule stability, and the biophysical constraints of the surrounding matrix. As highly mobile immune sentinels, T cells prioritize rapid migration to fulfill immune surveillance functions. Unlike cancer and stromal cells, which often degrade the ECM via protease secretion and rely on stable adhesions for movement, T cells preferentially navigate low‐resistance paths by probing the ITME for preexisting pores rather than enzymatically remodeling barriers [49]. Although capable of integrin‐dependent migration, T cells form only transient adhesive contacts to sustain motility. Their dominant migratory mode, deformational migration, integrates membrane plasticity, actomyosin‐driven contraction, and traction forces to propel cells through confined spaces. This process is governed by Rho/ROCK signaling and myosin II activity, which regulate cortical contractility. Experimental evidence confirms that myosin II activation or Rho/ROCK pathway stimulation promotes deformational migration. Notably, optimal geometric confinement is essential for this mode, as T cells utilize leading‐edge protrusions to sense paths while retrograde actin flow translates contractile forces into propulsion [50].

Cell migratory capacity is intrinsically linked to mechanical compliance, with higher cellular deformability facilitating contractile migration through confined environments. This mechanical adaptability is largely governed by nuclear physical properties. Notably, CAR‐T cells traversing endothelial barriers may sustain nuclear damage, potentially impairing their immune effector functions [51]. Nuclear stiffness is regulated by two critical factors: (i) lamin A/C expression and (ii) chromatin condensation state. These components modulate cell–cell and cell–matrix interactions via cytoskeletal networks and mechanotransduction pathways mediated by the LINC (linker of nucleoskeleton and cytoskeleton) complex, which bridges the nuclear envelope to the cytoskeleton. Mechanistically, lamin A imparts plasticity to the nucleus, resulting in irreversible deformation post‐migration, whereas lamin B confers elasticity, enabling shape recovery [52]. Intriguingly, while elevated lamin A expression restricts transit through sub‐3‐µm constrictions, it simultaneously enhances stress resistance by upregulating the DNA repair chaperone HSP90, thereby promoting cell survival. This dual role underscores an evolutionary trade‐off between migratory efficiency and stress adaptation [53].

As critical mechano‐sensors, cells dynamically perceive and adapt to extracellular mechanical cues, modulating their migration strategies accordingly. Studies reveal stark contrasts in cellular responses to substrate stiffness: soft matrices elevate lamin A/C phosphorylation, whereas stiff substrates enhance lamin A/C stability through myosin II‐mediated tension, increasing nuclear rigidity [54]. Mechanical signaling further governs T cell activation and cytotoxic function, with matrix stiffness directly influencing their chemotactic (durotactic) and contact‐guided migration in 3D environments [55]. T cells probe mechanical cues via TCRs, which exert ∼100 pN traction forces, and preferentially migrate along aligned ECM fibers. While moderate spatial confinement and matrix organization facilitate rapid amoeboid migration, excessive physical constraints impair T cell function. Key obstacles include: (1) collagen density: High‐density collagen deposition with extensive cross‐linking within triple‐negative breast cancer inhibits CD8+ T cell proliferation and antitumor activity; (2) fiber architecture: perivascular niches and tumor margins frequently feature aligned stromal fibers that impede T cell infiltration [56].

### 3.3 Biochemical Suppression

Within the tumor microenvironment, the localized accumulation of inhibitory chemokines and cytokines, including CXCL12, TGF‐β, IL‐10, IL‐4, IL‐35, as well as reactive oxygen species (ROS), lactate, prostaglandin E2 (PGE2), and adenosine, represents a primary barrier to immune response [57]. The capacity of immune cells to detect and migrate toward tumor‐specific sites serves as a critical prerequisite for immune evasion and infiltration. However, as a protective physiological mechanism, the chemokine ligands produced by tumors remain insufficient to fulfill this demand, thereby impeding locally administered CAR‐T cells from efficiently executing chemotactic migration in the absence of biochemical cues. Even when CAR‐T cells traverse physical barriers and chemical resistance to accumulate near tumor sites, stromal cells and immunosuppressive cell populations significantly diminish their tumor‐targeting efficacy [58]. Cancer‐associated fibroblasts (CAFs), ubiquitous in the periphery of solid tumors, constitute a vital component of the ECM and play a pivotal role in sustaining and amplifying the ITME. During tumor progression, CAFs secrete abundant extracellular matrix components, cross‐linking enzymes like LOX and LOXL family proteins, and cytokines that fortify tumor defense mechanisms and enhance the structural integrity of physical barriers through collagen fiber remodeling. Studies demonstrate that immunosuppressive factors such as TGF‐β, highly expressed in the ITME, originate predominantly from CAFs through paracrine signaling. These cytokines induce T‐cell anergy via SMAD‐dependent pathways and obstruct their penetration into tumor cores by upregulating junctional adhesion molecules [59]. Furthermore, CAFs suppress antitumor immunity by upregulating PD‐L2 and FASL through NF‐κB activation, enabling antigen‐dependent cross‐presentation via MHC class I molecules and subsequent CD8+ T‐cell depletion through caspase‐mediated apoptosis [60].

Beyond CAFs, tumors harbor extensive immunosuppressive cell populations that impede both innate and adaptive immunity through redundant molecular mechanisms. Tumor‐associated macrophages (TAMs), constituting over 50% of immunosuppressive cells, predominantly exhibit an M2‐like phenotype characterized by CD163 and CD206 surface markers [61]. M2 macrophages recruit TAMs via CCL2 secretion through CCR2 receptor signaling and promote tumor progression through the release of epidermal growth factors like amphiregulin, angiogenic factors such as VEGF‐A, and inhibitory cytokines such as IL‐10 and TGF‐β that activate STAT3 phosphorylation pathways [62]. Myeloid‐derived suppressor cells (MDSCs), immature immune cells derived from bone marrow precursors, are recruited to tumor sites by chemokines including CCL1, CCL2, CCL5, and CXCL5 through CXCR2 interactions, where they suppress systemic immunity via arginase‐1‐mediated L‐arginine depletion. Monocytic MDSCs attenuate T‐cell responses by elevating nitric oxide derivatives through iNOS overexpression while simultaneously blocking T‐cell receptor ζ‐chain expression. Additionally, TAMs secrete IL‐4, IL‐10, and arginase‐1 to further recruit MDSCs into the ITME through JAK/STAT6‐dependent chemotaxis, thereby inhibiting cytotoxic T‐cell activity via PD‐1/PD‐L1 axis potentiation [63]. CD4+Foxp3+ regulatory T cells (Tregs) represent another highly infiltrative immunosuppressive population within the ITME, utilizing multiple synergistic mechanisms, including contact‐dependent suppression through membrane‐bound TGF‐β. Through cytokine secretion (e.g., IL‐10, IL‐35, TGF‐β) that activates SMAD7 signaling, upregulation of cytotoxic T‐lymphocyte‐associated antigen 4 (CTLA‐4) that outcompetes CD28 co‐stimulation, and inhibition of the CD80/CD86 co‐stimulatory axis via transcriptional repression, Tregs competitively bind IL‐2 through high‐affinity CD25 receptors and antagonize effector T‐cell functions by sequestering mTOR activation signals, thereby enforcing immune tolerance through epigenetic modification of T‐cell effector genes [64].

Adenosine, a pivotal immunosuppressive metabolite within the ITME, synergistically impedes CAR‐T cell activation, proliferation, and effector function through dual mechanisms involving extracellular receptor signaling and intracellular metabolic perturbation. Adenosine accumulation in the ITME primarily arises from the enzymatic hydrolysis of extracellular adenosine triphosphate (ATP). Under hypoxic and inflammatory conditions, tumor cells and immune cells exhibit widespread surface expression of the ectoenzymes CD39 (ectonucleoside triphosphate diphosphohydrolase‐1) and CD73 (5'‐nucleotidase) [65]. These enzymes catalyze the sequential conversion of immunostimulatory ATP into adenosine, which possesses potent immunosuppressive activity, thereby establishing a profoundly immunosuppressive local microenvironment. In detail, adenosine‐mediated immune suppression is primarily achieved through activation of the adenosine A2A receptor (A2AR) on CAR‐T cells. A2AR, a G protein‐coupled receptor, binds adenosine and activates adenylate cyclase. This results in a sustained elevation of intracellular cyclic adenosine monophosphate (cAMP), the second messenger, which subsequently activates protein kinase A (PKA). The PKA signaling cascade exerts multiple inhibitory effects: (i) It directly interferes with activation signaling downstream of both TCR and CAR, suppressing the nuclear translocation and transcriptional activity of key transcription factors such as NF‐κB and NFAT; (ii) It induces the expression of inhibitory receptors, including PD‐1 and lymphocyte activation gene‐3 (LAG‐3), on CAR‐T cell surfaces, thereby promoting an exhaustion phenotype; (iii) It significantly inhibits the synthesis and secretion of effector cytokines (e.g., IFN‐γ, TNF‐α) and impairs the release of cytotoxic granules (e.g., granzyme B), directly compromising cytotoxic efficacy against tumor cells [66]. Beyond the classical receptor pathway, recent studies indicate that activated CAR‐T cells actively import extracellular adenosine via the equilibrative nucleoside transporter 1 (ENT1). Intracellular adenosine is subsequently phosphorylated by adenosine kinase to form AMP. This elevates the intracellular AMP/ADP ratio, leading to allosteric inhibition of inosine monophosphate dehydrogenase (IMPDH), the rate‐limiting enzyme in the de novo pyrimidine synthesis pathway. Consequently, this inhibition directly hinders the synthesis of cytidine triphosphate (CTP) and uridine triphosphate (UTP), creating a deficit in the raw materials essential for DNA and RNA synthesis. This metabolic blockade induces CAR‐T cell cycle arrest at the S phase, fundamentally inhibiting clonal expansion and long‐term persistence [67].

Lactate‐driven metabolic stress within the ITME synergistically impairs CAR‐T cell effector function, clonal expansion, and long‐term persistence through three interconnected mechanisms: ITME acidification, disruption of critical metabolic pathways, and functional reprogramming. This metabolic perturbation represents a core impediment to the efficacy of adoptive cell immunotherapy for solid tumors. Within the solid tumor ITME, tumor cells exhibit a pronounced reliance on aerobic glycolysis (the Warburg effect) to sustain rapid proliferation, even under normoxic conditions. This metabolic phenotype results in substantial lactate production and efflux, primarily mediated by monocarboxylate transporters (MCTs, predominantly MCT4). Consequently, the ITME undergoes significant acidification (pH typically declining to 6.0–6.5) and accumulates elevated lactate concentrations (up to 10–30 mm) [68]. This lactate‐induced metabolic stress imposes multifaceted suppression on infiltrating CAR‐T cells. First, extracellular acidification exerts direct inhibitory effects, causing broad functional impairment. The low‐pH microenvironment impairs perforin polymerization and pore‐forming efficiency on target cell membranes, attenuates granzyme B‐dependent cytotoxicity, and impedes full CAR‐T cell activation by disrupting kinase activity and ion channel function dependent on TCR/CAR signaling. Concurrently, it disrupts ligand binding and internalization of chemokine receptors (e.g., CXCR3), impairing CAR‐T cell migration and tumor core infiltration. Second, lactate reprograms intracellular signaling and metabolism within CAR‐T cells, exacerbating functional impairment. Lactate functions not only as a metabolic end‐product but also as a signaling molecule and competitive metabolic substrate. Lactate specifically activates the G protein‐coupled receptor GPR81 expressed on CAR‐T cells [69]. Sustained GPR81 activation elevates cyclic adenosine monophosphate (cAMP) levels, activating protein kinase A (PKA). PKA subsequently inhibits the mechanistic target of rapamycin complex 1 (mTORC1) signaling pathway and key transcription factors (e.g., NFAT, MYC), ultimately reducing cytokine production (e.g., IFN‐γ, IL‐2) and arresting proliferation. Furthermore, lactate influx into CAR‐T cells, primarily via MCT1, is catalyzed by lactate dehydrogenase A (LDHA) to pyruvate, concomitant with NAD^+^ reduction to NADH. This reaction elevates the intracellular NADH/NAD^+^ ratio, which feedback‐inhibits the glycolytic rate‐limiting enzyme glyceraldehyde‐3‐phosphate dehydrogenase (GAPDH), thereby constraining the glycolytic flux essential for CAR‐T cell activation and effector function [70]. Elevated pyruvate and NADH levels may also compromise mitochondrial oxidative phosphorylation efficiency and promote ROS accumulation, further exacerbating cellular dysfunction. Third, lactate promotes CAR‐T cell depletion through negative regulation of epigenetic states and differentiation. Prolonged exposure to high‐lactate environments induces alterations in the CAR‐T cell epigenome [71]. For instance, lactate‐derived pyruvate can modulate the activity of α‐ketoglutarate‐dependent histone demethylases (e.g., KDM5), potentially increasing repressive histone modifications at genes critical for memory phenotype maintenance and effector function. Concurrently, acidosis and metabolic stress cooperatively upregulate expression of multiple inhibitory receptors (e.g., PD‐1, TIM‐3, LAG‐3), driving CAR‐T cell differentiation toward terminally exhausted and senescence‐like phenotypes [72].

## 4 The Application and Translation Boundaries of Cytokine‐Engineered CAR‐T Cells

Since promising clinical outcomes have shown in curing hematological malignancies, the possibility of CAR‐T cells revolutionizing the therapeutic efficiency of solid tumors has aroused increasing attention. However, there are still some obstacles as above‐mentioned restricted the accessibility of CAR‐T cells treating solid tumors in trials. Cytokines, characterized by natural immunoregulatory function, have been considered as the optimal option joining immunotherapy to compensate for these disadvantages. Successful clinical trial results of cytokines focused on tumor treatment are presented in Table 1. In this section, recent advances and potential limitations in cytokine‐engineered CAR‐T cells are discussed. Cytokine payloads and delivery modalities, alongside intended functional benefits and associated toxicity considerations, are summarized in Table 2.

**TABLE 1:** Cytokine‐based immunotherapy for cancer: Clinical trial results.

Clinical trial results in the early stage			
Investigators	Intervention	Cancer type and number of patients	Study phase
Rook et al.	Intralesional or s.c. IL‐12 monotherapy	10 patients with cutaneous T cell lymphoma	Phase I
Duvic et al.	IL‐12 monotherapy	23 patients with early‐stage mycosis fungoides and received at least 3 prior anti‐lymphoma therapy	Phase II
Younes et al.	i.v. or s.c. IL‐12	32 patients with non‐Hodgkin lymphoma (NHL) and 10 patients with Hodgkin lymphoma (HL)	Phase II
Ansell et al.	s.c. IL‐12 combined with rituximab	43 patients with CD20 + NHL, including indolent NHL, diffuse large B‐cell lymphoma (DLBCL), and mantle cell lymphoma	Phase I
Ansell et al.	s.c. IL‐12 combined with rituximab or rituximab followed by s.c. IL‐12	58 patients with relapsed B‐cell NHL were randomized to receive rituximab combined with subcutaneous IL‐12	Phase II
Robertson et al.	IL‐18 combined with rituximab	19 patients with CD20+ NHL	Phase I
Robertson et al.	IL‐18 combined with ofatumumab	9 patients with NHL, including 7 with DLBCL	Phase I
Timmerman et al.	IL‐21 combined with rituximab	21 patients with indolent R/R NHL IL‐21 combined with rituximab, including 9 patients with FL and 1 patient with marginal zone lymphoma.	Phase I

Clinical trials			
Cytokine	Combination therapy agents	Cancer types	Clinical Trials.gov identifier
IL‐2	Nivolumab	Melanoma, renal cell carcinoma	NCT03991130
IL‐2	Pembrolizumab	Renal cell carcinoma	NCT02964078
IL‐2	Nivolumab; ipilimumab	Melanoma	NCT04562129
IL‐2	Enoblituzumab	Ovarian cancer	NCT04630769
IL‐2	TASO‐001	Solid tumors	NCT04862767
IL‐12	DC/tumor vaccine	Glioma	NCT04388033
IL‐12	Cetuximab	Head and neck cancer	NCT01468896
IL‐12	Pembrolizumab	Solid tumors	NCT03030378
IL‐15	Mogamulizumab	Cutaneous T cell lymphoma	NCT04185220
IL‐15	Nivolumab; ipilimumab	Solid tumors	NCT03388632
IL‐15	Avelumab	Cutaneous T cell lymphoma, Polymorphic T‐cell lymphoma	NCT03905135

**TABLE 2:** Cytokine payloads, delivery modalities, intended functional benefits, and associated toxicity considerations.

Payload	Delivery modality	Intended functional benefits	Toxicity consideration	Ref.
IL‐12	Constitutive	Saved exhaustion and dysfunction of tumor‐infiltrating CAR‐T cells. Further reversed TME through decreased numbers of both CD4+ T cells and Tregs.	Local delivery of IL‐12 avoids systemic immune activation effects.	[97]
IL‐12	Exogenous attachment and drug‐controlled delivery	Three‐fold more infiltration in Raji cell spheroids interior zone was observed in the INS‐CAR T group.	INS‐CAR T‐mediated IL‐12 delivery effectively decreased the free IL‐12‐triggered inflammatory response and thus improved the biosafety of the combination therapy in vivo.	[98]
IL‐12	Non‐genetic extracellular cytokine delivery	Amplify CAR‐T cell‐mediated tumor killing with antigen‐dependent manner. achieved excellent antitumor effect in various tumor models.	Collagen‐binding domain‐IL‐12 fusion cytokine achieved tumor‐specific enrichment and reduced circulating IL‐12 levels.	[99]
IL‐12	Constitutive	Boost the function of CD19 CAR‐T cells with fewer side effects.	Oxygen degradation domain of HIF1α limits IL‐12 secretion only at the tumor site and improves treatment safety.	[100]
IL‐12	MSC‐mediated delivery of cytokine‐armed oncolytic virus	Predictable cancer cell lysis and ITEM disruption.	MSC carriers express a low level of IL‐12 in circulation, while robust viral replication and IL‐12 production occur only after transfer to tumor cells.	[105]
IL‐12	DC‐mediated constitutive cytokine expression	Significantly ameliorated immunosuppressive ITME.	cDC1‐committed cells initiate long‐lasting immune responses and persist in sizable numbers for only several days.	[105]
IL‐2	Intraperitoneal injection	Promoted proliferation and response of IL‐2Rβ‐expressed T cells and evaded dose‐induced immunotoxicity of wild‐type IL‐2 skillfully.	Orthogonal IL‐2/IL‐2Rβ pair restricts cytokine signaling to engineered CAR‐T cells exclusively.	[110]
IL‐2	synNotch	Achieve foci‐targeted production of synthetic IL‐2 in a TCR/CAR‐independent manner, which could help bypass tumor immune suppression and enhance CAR‐T cells infiltration and expansion only limited to the tumor lesions.	SynNotch enables antigen‐restricted, tumor‐localized IL‐2 expression, which boosts CAR‐T function while avoiding the systemic toxicity of IL‐2.	[111]
IL‐15	Constitutive	Enhance the expansion, survival, and central memory phenotype of CAR‐T while remodeling the immune microenvironment.	Used second and fourth generation CAR‐T without any specific mention of biosafety considerations.	[116]
IL‐15	Constitutive	Potent antitumor activity in vitro and in vivo, with superior tumor control in serial tumor rechallenge assays.	No GvHD, CRS, or histologic toxicity reported.	[124]
IL‐15	Constitutive	Increases the expansion, intratumoural survival, and antitumour activity of GPC3 CAR T cells in patients.	Although CRS was more common in 15. CAR versus CAR T cell‐treated patients, IL‐15 serum concentrations were not higher, suggesting that these events were probably due to marked T cell activation.	[129]
IL‐18	Constitutive	Broadened target engagement to reduce antigen escape, with enhanced persistence and immune activation via 4‐1BB signaling and IL‐18 secretion.	Attenuated CAR signaling and avoidance of overly strong costimulation, with iCasp9 included to control potential cytokine toxicities.	[130]
IL‐18	Constitutive	Broadened target engagement to reduce antigen escape, with enhanced persistence and immune activation via 4‐1BB signaling and IL‐18 secretion.	Attenuated CAR signaling and avoidance of overly strong costimulation, with iCasp9 included to control potential cytokine toxicities.	[131]
IL‐18	Constitutively active IL‐18 receptor	Counter T‐cell dysfunction in antigen‐persistent solid tumor microenvironment, sustaining MyD88 signaling under chronic antigen exposure.	Design‐level toxicity mitigation was not explicitly engineered into the Zip18R CAR construct, and safety concerns are acknowledged.	[132]
IL‐18	Constitutive	Constitutively secreting IL‐18 to enhance antitumor activity and local immune activation. The 3‐day manufacturing process is designed to preserve stem‐cell–like characteristics and reduce exhaustion in T cells.	Incorporated a humanized anti‐CD19 scFv to mitigate immunogenicity. No HLH‐like syndrome/unexpected AEs observed.	[133]
IL‐18	Constitutive	Fully human anti‐CD371 armored CAR‐T with modified CD28 + constitutive IL‐18 to boost CAR‐T persistence and to modulate the AML‐associated immune microenvironment.	Five cases of CRS were observed in the clinical trial, with two cases reaching grades 3–4.	[134]
IL‐18	Constitutive secretion of GrB‐cleavable IL‐18	Enhance local CAR‐T function and metabolic fitness.	Protease‐activated cytokine limits systemic IL‐18 toxicity.	[135]
IL‐18	Inducible	Achieving localized release and accumulation of IL‐18 in ITME through NFAT response elements.	GD2 engagement–dependent IL‐18 production mitigates risks of systemic immune activation.	[136]
IL‐23	Constitutive p40 subunit expression enabling IL‐23 assembly	Boost CAR/TCR‐T proliferation, survival, persistence, and effector function in solid tumors via STAT3‐linked IL‐23 signaling.	Activation‐restricted autocrine IL‐23 limits bystander activation.	[137]

### 4.1 What Has Changed Since 2023?

Tunable cytokine signaling circuits represent advanced methodologies employing synthetic biology principles to achieve modularization and programmable engineering of cytokine signaling pathways within CAR‐T cells [73]. These circuits enable quantitative, dynamic, and on‐demand cellular responses to specific external stimuli or internal states, thereby facilitating precise regulation of cellular behavior. The core objective is to address limitations inherent to natural cytokine signaling in immunotherapy, such as toxicity and insufficient efficacy, ultimately achieving precise control over the therapeutic window [74]. A typical tunable signaling circuit generally comprises the following key modules. First, the sensing/input module detects specific signals and transduces them into processable internal instructions. This module is typically engineered as synthetic receptors responsive to diverse inputs, including small‐molecule drugs (e.g., tetracycline, rapamycin), specific antigens, light, or biomarkers (e.g., hypoxia, elevated ATP concentrations) [74]. Second, the processing/regulation module executes logical operations and amplifies the input signal to achieve precise signal control. Common implementations utilize transcriptional regulatory systems (e.g., Tet‐On/Off), protein‐protein interaction systems (e.g., FKBP‐FRB dimerization), or CRISPR‐dCas9 systems to regulate the expression levels of downstream effector genes, enabling signal “switching,” “modulation,” or “delay” [75]. Third, the output/effector module executes predetermined cellular functions, primarily mediating the controlled expression and secretion of specific cytokines. This module focuses on engineering customized expression units for cytokines (e.g., IL‐2, IL‐12, IL‐15, IL‐18) or their cognate receptors. Expression level, timing, and duration are precisely governed by the upstream regulatory module [76]. Fourth, the feedback/self‐stabilization module maintains system homeostasis and prevents excessive signal amplification or dysregulation [77]. This module is commonly designed as a negative feedback loop; for instance, elevated output cytokine concentrations can autonomously inhibit further cytokine production, thereby mimicking endogenous organismal homeostasis mechanisms. Chen et al. innovatively developed an endogenous gene reprogramming strategy based on CRISPR gene editing. By screening tumor‐specifically expressed NR4A2 and RGS16 promoters, the researchers successfully enabled the targeted delivery of cytokines such as IL‐12 and IL‐2 to tumor sites [78]. This study significantly enhanced the antitumor efficacy in murine allograft and xenograft models, while improving the polyfunctionality of CAR‐T cells and activating endogenous antitumor immunity, with favorable safety profiles demonstrated. Bell et al. designed chimeric cytokine receptors modified with leucine zippers to optimize the specificity of JAK/STAT signaling pathway activation [79]. This modification allows CAR‐T cells to survive in cytokine‐deprived environments, thereby enhancing their persistence and therapeutic efficacy. Meanwhile, it avoids bystander immune toxicity triggered by the activation of wild‐type cytokine receptors. In lymphoma xenograft models, the engineered CAR‐T cells exhibited enhanced antitumor effects without obvious systemic inflammatory responses. Zheng et al. constructed a TGF‐β signal‐inverting cytokine receptor (TB15), which converts the immunosuppressive signal of TGF‐β into the pro‐activating signal of IL‐15 [80]. This receptor not only blocks TGF‐β‐mediated CAR‐T cell exhaustion in the tumor microenvironment to boost therapeutic efficacy, but also circumvents the toxicity caused by additional cytokine infusion. In solid tumor models with high TGF‐β expression, the persistence of CAR‐T cells was tripled without an increase in toxicity.

The orthogonal IL‐2/IL‐2R systems were first conceptualized in 2018. Their design rationale and objective centered on establishing a functionally independent and signal‐specific synthetic cytokine signaling pathway. This approach aimed to overcome the limitations hindering the clinical translation of natural IL‐2, which stem from its pleiotropic effects and the ubiquitous expression of its cognate receptor. Leveraging the principle of “orthogonality” in synthetic biology, protein engineering was employed to generate a novel pair comprising IL‐2 mutants (orthoIL‐2) and their corresponding receptor mutants (orthoIL‐2Rβ) [81]. These engineered components engage in specific binding interactions, transmitting pro‐proliferation and pro‐survival signals analogous to natural IL‐2, yet crucially, they operate without cross‐reactivity within the endogenous IL‐2 system, thereby ensuring safety. When bound to CAR‐T cells, orthoIL‐2 circumvents persistent activation of these cells by other immune cells expressing wild‐type IL‐2 receptors in vivo. This strategy thus resolves the inherent efficacy‐toxicity contradiction associated with natural IL‐2 therapy and traditional armored CAR‐T approaches, while simultaneously preventing CAR‐T cell exhaustion induced by sustained stimulation [82]. Aspuria et al. developed an orthogonal IL‐2 receptor‐ligand system that enables specific in vivo regulation of CAR‐T cell expansion and activation. In this system, the engineered orthogonal human IL‐2 (STK‐009) can selectively bind to the orthogonal human IL‐2 receptor β‐chain (hoRb) expressed on the surface of CAR‐T cells [83]. Experiments have confirmed that STK‐009 can promote the expansion of hoRb‐expressing CAR‐T cells and maintain the phenotypes of stem cell memory T cells (TSCM) and effector T cells, regardless of the presence or absence of tumor antigens. In preclinical models of human CAR‐refractory lymphoma, STK‐009 treatment drove the systemic and intratumoral expansion and activation of hoRb‐expressing anti‐CD19‐CD28ζ CAR‐T cells (SYNCAR cells). By virtue of the selective in vivo expansion and activation of CAR‐T cells, this orthogonal IL‐2 receptor‐ligand system can still induce complete remission of large subcutaneous lymphomas even when the CAR‐T cell infusion dose is substantially reduced. In addition, withdrawal of STK‐009 allows for physiological contraction of CAR‐T cells, thereby effectively limiting the CRS induced by tumor antigen‐specific T cell activation. More detailed applications of orthogonal IL‐2/IL‐2R systems are discussed in “***4.3. IL‐2 cytokine signaling***.”

By integrating synthetic biology, materials science, and protein engineering technologies, synthetic cytokine scaffolds achieve precise delivery, signal regulation, and functional expansion of cytokines and have become a key direction for breaking through the bottlenecks of traditional cytokine therapy. David et al. developed T‐cell Enhancing Scaffolds (TES): subcutaneously injectable biomaterials composed of IL‐2 mesoporous silica rods (MSRs) with precisely controlled pore sizes (200–500 nm diameter). These MSRs were functionalized via covalent conjugation of T‐cell activating ligands (αCD3/αCD28 antibodies) at optimized surface densities (50–200 µg cm^−2^) [83]. This design generates spatiotemporally regulated synthetic immune niches that actively recruit circulating CAR‐T cells via chemokine gradients (CCL19/CCL21), deliver coordinated Signal 1 (TCR) and Signal 2 (co‐stimulatory) activation through ligand‐presenting surfaces, and enable effector cell egress via tunable biodegradation kinetics (4–6 week half‐life) [84]. Importantly, TES overcomes endogenous APC dysfunction by providing supraphysiological stimulation while avoiding systemic toxicity through local containment. This stands in stark contrast to nanovaccine strategies, which carry risks of uncontrolled systemic antigen distribution. To target CNS disorders with precision, Roybal et al. engineered brain‐sensing synNotch T cells, a programmable cellular platform. Central to this platform are CNS‐specific extracellular matrix ligands (e.g., brevican/BCAN), identified via transcriptomic mining and used as anatomical “GPS coordinates” [85]. The T cells incorporate protease‐cleavable synthetic Notch receptors; these receptors are engineered with anti‐BCAN single‐chain variable fragments (scFvs) and undergo orthogonal γ‐secretase‐mediated cleavage upon binding brain antigens. This cleavage event triggers localized transcriptional induction of therapeutic payloads solely within the CNS parenchyma. The design enables dual‐layer spatial control: first, anatomical specificity is achieved through blood‐brain barrier trafficking and brain‐restricted antigen recognition; second, payload confinement is realized via inducible expression kinetics (6–24 h post‐activation) mediated by optimized nuclear localization signal‐P65 fusion constructs. A key advantage is its elimination of systemic payload exposure, which resolves the fundamental delivery‐toxicity paradox in CNS therapeutics. When tested in murine models of glioblastoma and breast cancer brain metastases, BCAN‐sensing T cells that induce localized CAR expression achieved 83% tumor reduction without peripheral off‐target engagement. Reddy et al. developed synthetic suppressor T cells (SSTs) by reprogramming conventional CD4^+^ T cells with synthetic Notch (synNotch) circuits. These circuits induce localized expression of combinatorial immunosuppressive payloads, exclusively upon antigen encounter at disease sites, including anti‐inflammatory cytokines (IL‐10, TGFβ1), checkpoint ligands (PD‐L1), and cytokine sinks (CD25). This design achieves spatially constrained immunosuppression via synNotch‐mediated transcriptional control, which restricts payload delivery to targeted tissues. This not only eliminates systemic toxicity but also mimics natural regulatory T cell function with enhanced customization. In therapeutic validation, SSTs acted as precision “NOT gates,” protecting normal organs from off‐tissue CAR‐T cytotoxicity with >90% efficacy without compromising antitumor activity. Concurrently, they provided 100% graft protection in pancreatic islet transplant models while preserving endocrine function and avoiding global immunosuppression. The integrated CD25 component formed a self‐amplifying feedback loop through IL‐2 consumption: this expanded SST populations while starving effector T cells, boosting local immunosuppressive potency by 3.7‐fold compared to single‐agent constructs. This platform establishes a customizable cellular delivery strategy for spatially restricted immune modulation, ideal for transplantation, autoimmunity, and cell therapy safety applications where targeted intervention is paramount [86].

Since its clinical breakthrough in 2011, CAR‐T therapy has been dominated by adoptive cell therapy. This approach involves extracting autologous immune cells from patients, genetically engineering them to express CAR ex vivo, and reinfusing the modified cells back into patients. By leveraging the patient's own immune system, it achieves disease remission and holds potential for the development of “universal” and “off‐the‐shelf” products [87]. However, this model is hindered by high costs and stringent technical barriers, which limit its accessibility and widespread application. Additionally, the prolonged ex vivo manufacturing process may induce phenotypic and functional alterations in CAR‐T cells, leading to product heterogeneity and elevated therapeutic risks [88]. In vivo CAR‐T cell induction technology has emerged from multidisciplinary advances to circumvent these limitations. This methodology employs vectors to deliver CAR‐encoding genetic material directly to target immune cells within the organism, thereby generating CAR‐expressing immune cells in situ. It significantly reduces manufacturing costs and timeframes while mitigating risks associated with ex vivo manipulation [89]. The selection of appropriate delivery vectors and methodologies constitutes the critical determinant for successful in vivo CAR‐T cell production. Three distinct technical platforms are currently employed for in vivo CAR‐T cell generation, including viral vector‐based in vivo CAR‐T generation, nanoparticle‐based in vivo CAR‐T generation, and in vivo implantation‐mediated CAR‐T generation. Currently, in vivo CAR‐T therapy has entered the clinical trial phase, with research covering B‐cell malignancies, leukemia, and autoimmune diseases. In antitumor applications, studies focus on well‐validated targets such as CD19, CD20, and BCMA. The preparation technology is mainly based on lentiviral vector‐mediated CAR gene delivery, and targeting strategies include CD3, CD8, CD7 molecules and anti‐TCR nanobodies. Limited preliminary clinical results have demonstrated promising potential: ESO‐T01, a CAR lentiviral vector targeting BCMA, was used to treat 4 patients with refractory multiple myeloma, among whom 2 achieved complete remission, and 2 achieved partial remission, with reduced tumor burden and negative minimal residual disease status [90]. JY231 was administered to 1 patient with relapsed/refractory diffuse large B‐cell lymphoma bearing a high tumor burden, leading to complete remission and successful discharge. Notably, the patient maintained stable vital signs and did not experience grade ≥ 2 CRS or neurotoxicity, adverse events that are prevalent in adoptive CAR‐T therapy [91]. These findings highlight the antitumor value and clinical feasibility of in vivo CAR‐T therapy. In the field of autoimmune diseases, relevant research is steadily advancing. Clinical trial NCT06917742 is currently recruiting healthy volunteers to evaluate the safety of CPTX2309, an mRNA‐lipid nanoparticle‐based in vivo CAR‐T product intended for the treatment of systemic lupus erythematosus (SLE) and Sjögren's syndrome [92]. HN2301, an engineered lipid nanoparticle targeting CD8^+^ T cells and encapsulating mRNA encoding anti‐CD19 CAR, was used to treat 5 patients with refractory SLE. Circulating B‐cell counts decreased sharply and were completely eliminated within 6 h after the first treatment, and all 5 patients showed significant improvement in their condition by the 3‐month follow‐up [93]. In summary, in vivo CAR‐T therapy boasts both logistical advantages and potent B‐cell depletion capacity. These findings fully confirm that this therapy is expected to become a novel strategy for the treatment of tumors and autoimmune diseases, with broad prospects for clinical translation.

Armored CAR‐T cells represent an engineered enhancement of conventional CAR‐T technology. Building upon the foundation of traditional CAR‐T cells, this approach incorporates one or more additional functional genes. These modifications facilitate the secretion of cytokines, expression of co‐stimulatory ligands, or counteraction of immunosuppressive pathways, thereby enabling the CAR‐T cells to better sustain their activity, proliferative capacity, and persistence within the tumor microenvironment. Consequently, armored CAR‐T cells exhibit an improved ability to overcome immunosuppressive barriers and enhance antitumor efficacy. Compared to traditional CAR‐T therapy, armored CAR‐T enhances the survival and functionality of CAR‐T cells in immunosuppressive tumor milieus. This is achieved through mechanisms such as the expression of immunostimulatory cytokines (e.g., IL‐12, IL‐15) or the antagonism of inhibitory signals (e.g., via PD‐1 blockade). Furthermore, continuous autocrine cytokine support prolongs the persistence duration of CAR‐T cells in vivo, promotes their expansion, and may mitigate tumor relapse. By exerting localized effects rather than inducing systemic high‐dose cytokine release, Armored CAR‐T has the potential to reduce the incidence of cytokine‐related adverse events, including severe CRS. The current clinical trials of armored CAR‐T cells are summarized in Table 3.

**TABLE 3:** Current clinical trials of armored CAR‐T cells.

Cytokine	Intervention	Cancer type	Study stage	Number of patients	Source
IL‐2	CD19 CAR‐T co‐expressing an engineered orthogonal IL‐2 receptor with exogenous orthogonal IL‐2 support	CD19+ Hematologic Malignancies	Phase I	36 estimated	NCT05665062
IL‐10	targeting CD19 and expressing IL‐10	Diffuse Large B‐cell Lymphoma	Early Phase I	18 estimated	NCT06120166
IL‐12	Anti‐CD19 19–28z CAR‐T co‐expressing IL‐12 and EGFRt	CD19+ Hematologic Malignancies	Phase I	1 actual	NCT06343376
IL‐15 IL‐21	GPC3 CAR‐T co‐expressing IL‐15 and IL‐21 with iCasp9 safety switch	solid tumor expressing GPC3	Phase I	21 estimated (not yet recruiting)	NCT07224568
IL‐15, IL‐21	GPC3 CAR‐T co‐expressing IL‐15 and IL‐21 with iCasp9 safety switch	GPC3+ solid tumors	Phase I	21 estimated	NCT06198296
IL‐18	Anti‐CD19 19–28z CAR‐T expressing IL‐18	Relapsed or Refractory Acute Lymphoblastic Leukemia	Phase I	18 estimated	NCT06287528
IL‐18	Nectin‐4‐targeted CAR‐T cell injection to induce IL‐18 secretion	Nectin‐4 + advanced solid tumors	Phase I	25 estimated	NCT07101549
IL‐18	targeting CD19 and expressing IL‐18	CD19+ Non‐Hodgkin lymphoma	Phase I	24 estimated	NCT05989204

### 4.2 IL‐12‐Based CAR‐T Cell Therapy

IL‐12, a heterodimeric cytokine, is secreted by antigen‐presenting cells that relates to multiple signal transduction processes, including pathogen‐associated molecular patterns, damage‐associated molecular patterns, cytokine stimulation, and direct immune cell‐cell contact [94]. IL‐12Rb1/IL‐12Rb2 heterodimers show continuous expression on the membrane of activated T cells and B cells, which share high binding affinity with IL‐12, and their combination can lead to activation of both JAK2 and TYK2, as well as downstream signal STAT4. Recent evidence has indicated that enhanced and catenated function of IL‐12 in the innate and adaptive immune system contributes to promising antitumor effects in preclinical immunotherapy. Through activating the production of IFN‐γ, IL‐12 secreted from NK and T cells to further promote differentiation both of naïve CD4 Th0 cells into Th1 cells and the CD8 T cells into CTL, which in turn has amplified tumor‐cytotoxicity and proliferation of CTL and NK cells. B‐cell survival and IgG production can also be enhanced through IL‐12 function [95, 96]. Mechanistically, potent antitumor capability of IL‐12 mainly attributed to reasons as following: (i) produce monokine induced by IFN‐γ (MIG; CXCL9) and IFN‐γ‐induced protein 10 (IP‐10; CXCL10) to functioning antiangiogenic effects; (ii) impair mechanical strength both of peritumoral extracellular matrix and tumor stroma, as well as up‐regulating the expression and cross‐presentation of MHC class I molecules to improve drug penetration and immune response. Regrettably, clinical studies have demonstrated that the systemic administration of IL‐12 is associated with unacceptable proinflammatory toxicity and may even result in fatal outcomes, severely limiting the direct application of IL‐12 in cancer treatment. Thus, a carefully managed approach is required urgently to deliver IL‐12 to the tumor site, which can induce tumor‐specific immune responses while minimizing systemic toxicity [97, 98].

**FIGURE 3:**
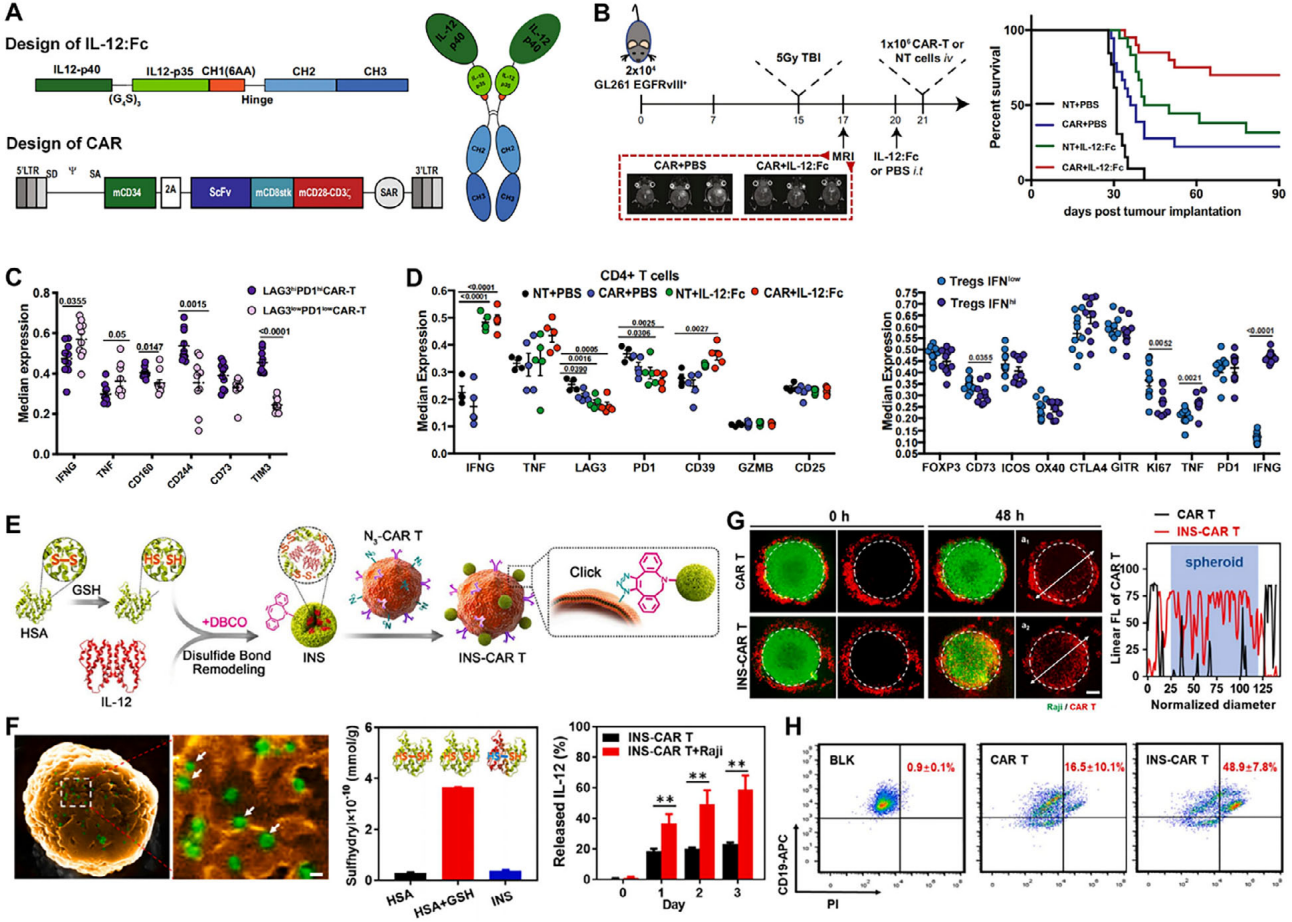
Evaluation of therapeutic efficacy in IL‐12 cytokine‐engineered CAR‐T cell immunotherapy. (A) CAR construct and scheme of the IL12:Fc construct, as well as a schematic representation of heterodimeric IL‐12 fused to the Fc portion of murine IgG3. (B) Pharmacodynamic investigation of locally administered IL‐12:Fc in orthotopic tumor‐bearing mice. (C) Median expression of selected cell markers shown for LAG3hiPD1hi CAR‐T cells and LAG3^low^PD1^low^ CAR‐T cells in various groups. (D) IL‐12 reprograms the endogenous T cell compartment within the glioma ITME. Figure 3A–D is reprinted with permission from ref. [99], Copyright 2021, Springer Nature. (E) Schematic illustration of INS‐CAR‐T biohybrids. (F) SEM images of INS‐CAR‐T. Scale bar = 100 nm. (G) Infiltration of INS‐CAR T and CAR T cells in Raji cell spheroids. (H) The death rate of tumor cells in 3D spheroids. Figure 3E–H are reprinted with permission from ref. [100], Copyright 2022, Elsevier.

In the previous study, recombinant single‐chain IL‐12 was first fused to the Fc portion of murine IgG3 (IL‐12:Fc) and combined with EGFRvIII‐oriented CAR‐T cells for glioblastoma treatment (Figure 3A). Combination of EGFRvIII‐specific CAR‐T cells and a single dose of locally administered IL‐12:Fc significantly controlled tumor growth and prolonged the mice's survival period (Figure 3B). Compared with single CAR‐T cell therapy, IL‐12:Fc prevented exhaustion and dysfunction of tumor‐infiltrating CAR‐T cells through reducing the expression of LAG3 and PD‐1, as well as up‐regulating the expression of TIM3, CD160, CD244, and CD73 (Figure 3C). The recruitment results of endogenous T cells to TEM showed that IL‐12:Fc reshapes ITME through reprogramming the distribution of CD4+T, CD8+T, and Treg cells. After IL‐12:Fc treatment, there was a great expression reduction of checkpoint receptors (LAG3 and PD1) in CD4+T cells and inhibitory molecules (CD25) in Treg cells, which indicated them from “stable” to “fragile.” Meanwhile, the increased frequency of IFN‐γ^hi^ Tregs in the presence of IL‐12:Fc and displayed low expression of CD73, ICOS, and OX40 (Figure 3D). Taken together, the data supported that locally delivered IL‐12 boosted cytotoxicity of CAR‐T cells, and further reversed TME through decreased numbers of both CD4+ T cells and Tregs [99]. However, security management and application costs are always concerns if IL‐12 administration is independent of CAR‐T. Cai et al. utilized the technology of “Click chemistry” to engineer IL‐12‐hybridized CAR‐T cells, which were used to achieve potent CAR‐T cell‐mediated tumor‐killing and ITME reconstruction [100]. IL‐12 assembled with HSA via intermolecular disulfide bond remodeling and modified with dibenzocyclooctyl (DBCO) groups (INS), to which finally clicked with azide‐sugar labeled CAR‐T cells (INS‐CAR‐T) (Figure 3E). After reaching the tumor site, the disulfide bond displayed redox‐responsive fracture, and IL‐12 was released to further enhance T cell infiltration (Figure 3F). Three‐fold more infiltration in the Raji cell spheroids' interior zone was observed in the INS‐CAR T group, whereas the CAR‐T cells group alone mainly existed in the spheroid edge, with few cells detected in the interior (Figure 3G). In addition, the cytotoxicity of INS‐CAR‐T was nearly three‐fold higher than that of the CAR‐T control group, confirming that the introduction of IL‐12 plays a significant role in T cell‐mediated tumor‐killing (Figure 3H). The design of membrane‐bound IL‐12 to engineer CAR molecules is an alternative method to manage therapeutic efficiency and safety. Priceman et al. explored the optimized transmembrane domain of IL‐12 (mbIL‐12), which directly assembled to the CAR molecule to amplify CAR‐T cell‐mediated tumor killing in an antigen‐dependent manner. With advanced multi‐objective optimization, including dCH2 as a hinge region, CD28tm as a transmembrane region, 4‐1BBz as a co‐stimulatory region, and mbIL‐12 as IL‐12 membrane‐binding formation, TAG‐72‐CAR/mbIL‐12 achieved excellent antitumor effect in various tumor models, including ovarian cancer, breast cancer, and further reversed ovarian cancer peritoneal metastasis.

**FIGURE 4:**
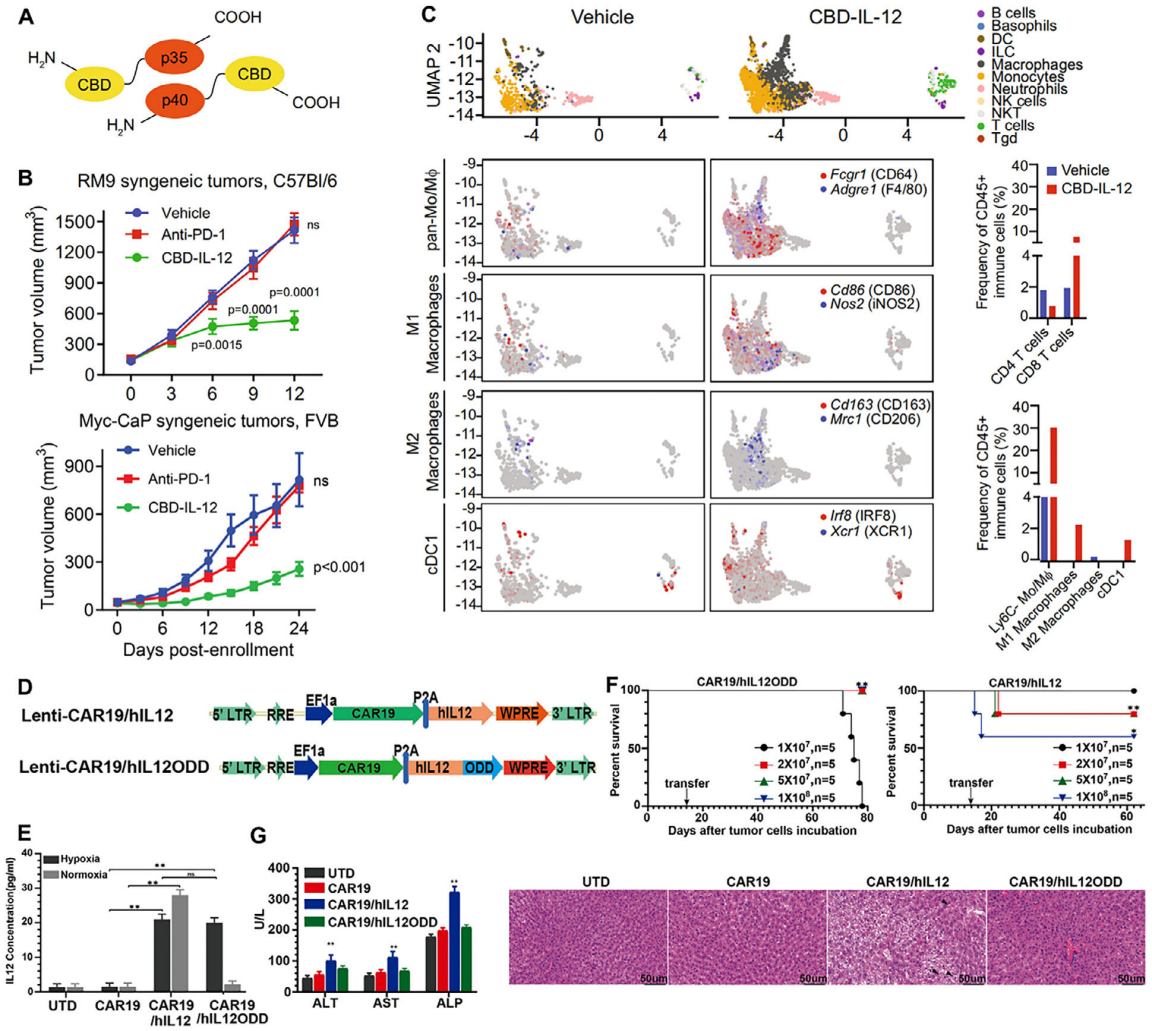
Evaluation of therapeutic efficacy in IL‐12 cytokine‐engineered CAR‐T cell immunotherapy. (A) Schematic of CBD‐IL‐12, composed of the p35 and p40 subunits fused to the CBD from von Willebrand factor domain A3. (B) Volumes of RM9 and Myc‐CaP subcutaneous tumors in syngeneic C57Bl/6 mice over time. (C) Different immune cell subsets (top) from single‐cell RNA‐seq (scRNA‐seq) analysis and UMAP plots colored with gene expression of immune cell subset‐specific markers, as well as their frequency of specific immune cell populations. Figures 4A–C are reprinted with permission from ref. [101], Copyright 2023, Springer Nature. (D) Schematic representation of CAR. (E) The secretion of bioactive IL‐12 p70 under hypoxic and normoxic environments. (F) Survival was monitored after treatment. (G) Security testing and representative histopathology of the liver after treatment. Figure 4D–G are reprinted with permission from ref. [102], Copyright 2023, Elsevier.

In addition to conventional IL‐12 combined with CAR‐T cells directly, alternative strategies to circumvent some crucial issues, such as dose‐limiting toxicity and off‐tumor targeting, have attracted much attention and rely on modifying IL‐12 structure. K. Lee et al. described a novel approach to fuse the von Willebrand factor A3 domain, as a typical collagen binding domain (CBD), with two subunits of IL‐12 (p40 and p35), which endowed these fused proteins with precise binding to exposed collagen in disordered tumor vasculature (Figure 4A) [101]. By localizing IL‐12 at tumor foci, systemic CBD‐IL‐12 administration resulted in a notable inhibition of tumor growth in both the syngeneic RM9 and Myc‐CaP models, which had previously demonstrated a limited response to anti‐PD‐1 therapy (Figure 4B). After treatment, obvious improvements were presented in CD8+ T cells (from 0.76% to 7.55%), cDC1 (from 0% to 1.33%), M1 polarized macrophages (from 0% to 2.26%), and CD64+F4/80+ monocytes/macrophages (from 4.43% to 29.2%), which indicated that the antitumor activity of CBD‐IL‐12 in tumor models mainly through reprogramming of the TEM and engagement of both the innate and adaptive immune systems (Figure 4C). However, the tumor has not been cured completely in this issue may indicate that the administration and dosage regimen need to be optimized, as well as the design of IL‐12 should be more adaptive to TEM characteristics. Hypoxia, a typical tumor feature, provides a perfect breakthrough point for regulating the efficiency of IL‐12 combined with CAR‐T therapeutic strategies. Wang et al. developed a novel IL‐12‐armed CAR‐T cell system, named CAR19/hIL12ODD‐T cells, which combined IL‐12 with the oxygen degradation domain (ODD) of HIF1α to limit IL‐12 secretion only present at tumor site and improve treatment safety (Figure 4D) [102]. CAR19/hIL12ODD‐T cells secreted IL‐12 equivalent to CAR19/hIL12‐T cells under hypoxic conditions, but decreased 80% after 2 h cultured under normoxic conditions, which demonstrated that CAR19/hIL12ODD‐T cells produced IL‐12 in a hypoxia‐dependent manner (Figure 4E). OCI‐Ly3 xenograft‐bearing ZZU001 mice were treated with CAR19/hIL12‐T cells or CAR19/hIL12ODD‐T cells to assess professionally hIL‐12‐induced inflammatory cytokine and hepatic dysfunction. CAR19/hIL12ODD‐T treated mice basically survived completely under all agents’ doses; however, various degrees of death (20%–40%) occurred within a week, and the dosage of 1 × 10^8^ resulted in 40% mice death within 4 days under the CAR19/hIL12‐T cell treatment (Figure 4F). Compared with CAR19/hIL12‐T cells‐treated mice, CAR19/hIL12ODD‐T cells‐treated mice presented equivalent levels to CAR19 or UTD‐T cell groups both in the levels of alanine aminotransferase (ALT), aspartate aminotransferase (AST), and alkaline phosphatase (ALP), as well as there were no obvious blood vessel congestion, apoptosis, and necrosis of hepatocytes, indicating that the introduction of ODD potent avoided IL‐12‐mediated hepatotoxicity (Figure 4G).

ITME, featured with regional hypoxia, low pH, nutritional deprivation, and immunosuppression, is a formidable barrier to the success of clinical CAR‐T cell therapies for solid tumors. Although combining with IL‐12 overcomes some intractable issues, such as improving CAR‐T cell infiltration and reprogramming immunocells expression, the potent immune‐inhibitory net, including ligands, chemokines, and negative cytokines produced by ITME, always diminishes CAR‐T cell responses and skew infiltrating myeloid cells to an inhibitory phenotype [103, 104]. Therefore, both disrupting ITME and favoring recruiting a broad immune response to promote T cell persistence are of great importance. The cooperation of CAR‐T cells and other immunostimulatory therapeutic agents, such as oncolytic viruses or other immunoregulated cells, has attracted much attention. Brenner et al. Developed a novel synergic‐nanosystem, which combined mesenchymal stromal cells (MSCs)‐loading binary vectors (CAd), oncolytic adenovirus (OAd), and helper‐dependent Ad expressing IL‐12 and PD‐L1 blocker (HDAd), with HER.2‐specific CAR‐T cells to incorporate predictable cancer cell lysis and TEM disruption [105]. After 5 days in H1650 GFP tumor spheroids, CAD‐RFP MSC group showed increased tumor‐spheroid penetration, approximately reaching 8 × 10^6^ total fluorescence intensity compared with the MSC group with no fluorescence intensity. Meanwhile, even reduced CAR‐T cell numbers from 50 (blue) to 12, CAd MSC also shared the greatest tumor killing capability. UMap analysis demonstrated that both CD4 and CD8 HER.2 CAR‐T cells cultured with CAd MSC amplified the secretion of IFN‐γ, GzmB, TNF‐α, and CCL11, which played an important role in increasing T cell adhesion and TEM reconstruction. Furthermore, the analysis of T cells in the blood, spleen, and tissues in the tumor re‐challenge mice after treatment by CAd MSC + CAR‐T cell also showed limited expression of Tim3, potent demonstrated enhanced T cell persistence. Conventional DCs (cDCs), the population in the process of DC development, are capable of antigen‐crossing presentation and regulating T cell function and have been proven to be implicated in the orchestration of antitumor immunity. Palma et al. developed IL‐12/FLT3L‐armed conventional type‐I DCs (DCP‐IL‐12/FLT3L) and synergized with anti‐GD2 CAR‐T cells, which successfully overcome the limitation of CAR‐T therapeutic efficacy caused by antigenic heterogeneity and ITME. Compared with untreated glioma‐bearing mice, tumor regression was observed after mice receiving DCP‐IL‐12/FLT3L or CAR‐T monotherapies. However, there were 80% mice presented tumor regression and remained tumor‐free until termination in the DCP‐IL‐12/FLT3L + CAR‐T group. Interestingly, abundant CD3+ T cell infiltrates persisted at the site where the tumor had fully regressed, which indicated that DCP‐IL‐12/FLT3L significantly ameliorated ITME and reversed the CAT therapy dilemma [106].

### 4.3 IL‐2 Cytokine Signaling

In the process of combating malignancies development, IL‐2, as the first cytokine for clinical translation, which possesses a 4 alpha helix structure and activates innate and adaptive immune responses. There are three IL‐2 receptor subunits as follows: IL‐2Rα (CD25), IL‐2Rβ (CD122), and the γ common chain (CD132). Compared with IL‐2Rα‐binded IL‐2 compounds, IL‐2Rβγc heterodimer combined with IL‐2 presents higher affinity, and the IL‐2Rαβγc appears to have the strongest affinity. IL‐2Rαβγc is constantly expressed on the Tregs and CD565^bright^ NK cells, while it is transiently expressed on activated CD4+ and CD8+ T cells. Through facilitating CD8+ T cells to CTL, IL‐2 is capable of activating an adaptive immune response with T cell enhanced proliferation. However, there is a contradiction that the proliferation and function of Tregs could also be produced by IL‐2 stimulation, which may intensify immunosuppressive TEM and compromise anticancer therapy [107, 108].

**FIGURE 5:**
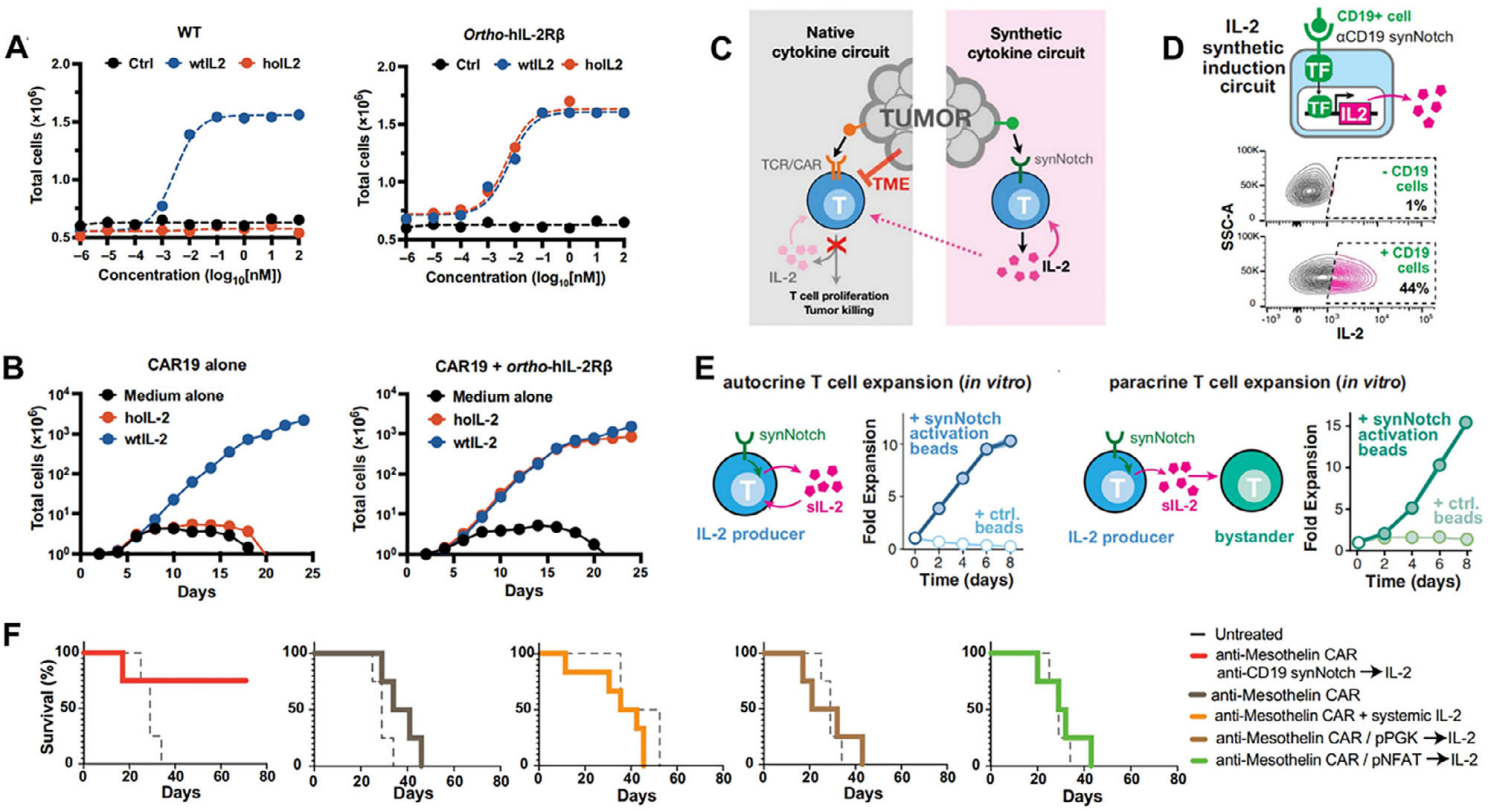
IL‐2 cytokine signaling assists improved CAR‐T therapy efficacy. (A) and (B) Viable cells were enumerated by trypan blue exclusion. Figure 5A,B is reprinted with permission from ref. [109], Copyright 2021, American Association for the Advancement of Science. (C) and (D) Synthetic synNotch→IL‐2 circuits can drive local T cell proliferation independent of TCR activation or cooperatively with T cell killing. (E) The synthetic IL‐2 circuit drives autocrine proliferation of primary human T cells (left) and synthetic IL‐2 circuit signals in a paracrine fashion to stimulate proliferation of a bystander population of human T cells that lack a synthetic circuit in vitro (right). (F) Survival rate after different treatments. Figure 5C‐‐F is reprinted with permission from ref. [110], Copyright 2023, American Association for the Advancement of Science.

To avoid the serious side effects, Sockolosky et al. used a directed evolution strategy to design an orthogonal (ortho) murine IL‐2 and IL‐2Rβ pair, which demonstrated that this orthogonal cytokine‐receptor pair promoted proliferation and response of IL‐2Rβ‐expressed T cells and evaded dose‐induced immunotoxicity of wild‐type IL‐2 [109] skillfully. The scope of this novel strategy was limited to mice in 2018; however, interventions of amino acid substitutions seem a promising choice to expand this approach into humans. Milone et al. Mutated “hotspot” residues of IL‐2Rβ (called ortho‐hIL‐2Rβ) to disrupt the binding between IL‐2Rβ and wild‐type human IL‐2, but only activated by ortho‐hIL‐2, which significantly limited CRS and immunosuppression caused by excessive secretion of IL‐2 [109]. H^133^D and Y^134^F were mutated in IL‐2Rβ, as well as Q22K and Q23A were mutated in IL‐2. SeAx, IL‐2‐dependent CD25+ cutaneous T cell lymphoma (CTCL) cell line, was used to support the proliferation specificity of ortho‐hIL‐2 activated by ortho‐hIL‐2R. Transduced with ortho‐hIL‐2Rβ‐T2A‐mCherry, ortho‐hIL‐2 enhanced the great expansion of SeAx cells that was close to wild type with analogous EC50, while ortho‐hIL‐2 concentrations exceeding the median EC50 of wild‐type IL‐2 by more than four logs failed to support the proliferation of wild‐type SeAx cells (Figure 5A). Furthermore, the proliferation enhancement function of CAR‐T cells was only activated when they were engineered with ortho‐hIL‐2Rβ, which increased by nearly 10^3^‐fold compared with CAR‐T cells alone at the same time (Figure 5B). The addition of ortho‐hIL‐2 improves the proliferation of T cells co‐expressing CART‐19 and ortho‐hIL‐2Rβ when expressed from a single vector and in response to CAR antigen restimulation by coculturing with irritated Nalm6 cells. Although direct transfection is capable of controlling cytokine release via the pairing effect as mentioned above, the complicated design and mutation have significantly hindered clinical translation. In addition, solid tumors generally create an immune‐excluded local microenvironment that blocks the activation of the TCR/CAR pathway. While tumor suppression reversal could be compromised by local administration of high‐dose cytokines, combining or engineering cytokines with T cells has shown obvious side effects and insufficient efficiency. Therefore, relying on the natural route, including the receptor‐mediated signaling pathway, to bypass the TCR/CAR pathway provides a novel point both for maximizing therapeutic efficacy and minimizing systemic toxicity. Wendell A et al. developed a synthetic Notch (synNotch) receptor to achieve foci‐targeted production of synthetic IL‐2 with TCR/CAR‐independent manner, which could help bypass tumor immune suppression and enhance CAR‐T cells infiltration and expansion only limited in the tumor lesions (Figure 5C) [110]. Tumor‐triggered circuits IL‐12 derived from CD19‐expressing T cells to improve transcription of super IL‐2 or sIL‐2 via synNotch sensor. The positive rate of IL‐2 was increased from 1% to 44% after adding CD19+ cells (Figure 5D). Only engineered with synNotch receptor, the expansion of T cells showed a potent increase via the autocrine pathway, as well as stimulating the expansion of synNotch receptor‐lacking T cells via the paracrine pathway (Figure 5E). In the pancreatic tumor‐bearing mice, the final survival days were extended for more than 80 days of CAR‐T/synNotch‐IL‐2 circuits, which were compared with other treatments of single CAR‐T with nearly 40 days, CAR‐T + Systemic IL‐2 administration with 50 days, CAR‐T + constitutive IL‐2 expression with 45 days, and CAR‐T + TCR/CAR activation with 45 days (Figure 5F).

### 4.4 IL‐15 Cytokine Signaling

Produced by antigen‐presenting cells, such as macrophages, monocytes, and dendritic cells, IL‐15 is structured with four alpha helices and specifically recognized by its heterotrimer receptor (IL‐15Rα (CD215)) through β subunit. The mRNA products of IL‐15 protein post‐translation share a great affinity with IL‐15Rα, and their compounds are transferred to the surface of cells. Additionally, IL‐15/IL‐15Rα complex is transpresented to the IL2/IL‐15Rβγc heterodimer expressed on NK and CD8 T cells. IL‐15 possesses potent immunostimulating ability by enhancing T cell proliferation, activation, and further differentiating into CTL. Recently, it has been demonstrated that IL‐15 may also play a crucial role in promoting prolonged expansion and activation of NK cells. There are some similar biological effects between IL‐15 and IL‐2, however, IL‐15 seems to represent more promising clinical translation owing to minor potential toxicity, including stimulating less Treg expansion, alleviating effector T cell death, and insignificant capillary leakage [111, 112, 113]. Battram et al. demonstrated that the function and expansion of ARI2h, a classical BCMA‐directed CAR‐T cell currently being administered to multiple myeloma patients, presented obvious improvement when cultured with IL‐15 rather than that of IL‐2 or IL‐15/IL‐7 [114]. Less‐differentiated cells with a more stem cell‐like phenotype could be conducive to CAR‐T cell products. Compared with IL‐2‐grown CARs, both IL‐15‐grown and IL‐15/IL‐7‐grown CARs displayed higher naïve CD4+ cells expression. Further investigation proved that many of the mice treated with ARI2h presented typical tumor recurrence at day 28, with those under the treatment of ARI2^hIL‐15/IL‐7^ experiencing more early recurrence than those of ARI2h^IL‐15^ or ARI2h^IL‐2^. Correspondingly, the ARI2h^IL‐15^ groups showed the longest survival time, as well as yielded spontaneous tumor clearance when new tumor development occurred. Following sacrifice, over 20% expression of CD3 in spleen and 1.5% expression of CD3 in bone marrow were found only in the ARI2h^IL‐15^‐treated mice, compared with 0.39% expression of CD3 in spleen and 0.09% expression of CD3 in the ARI2h^IL‐2^‐treated mice. Molecular level confirmed that the CD8+ cells from ARI2h^IL‐15^‐treated mice expressed intermediate levels of the exhaustion markers PD‐1, TIM‐3, and TIGIT, but low levels of LAG‐3, which may explain why there were optimal therapeutic effects with enhanced CAR‐T persistence. From those results, IL‐15 has aroused much interest in overcoming obstacles in the CAR‐T immunotherapies.

**FIGURE 6:**
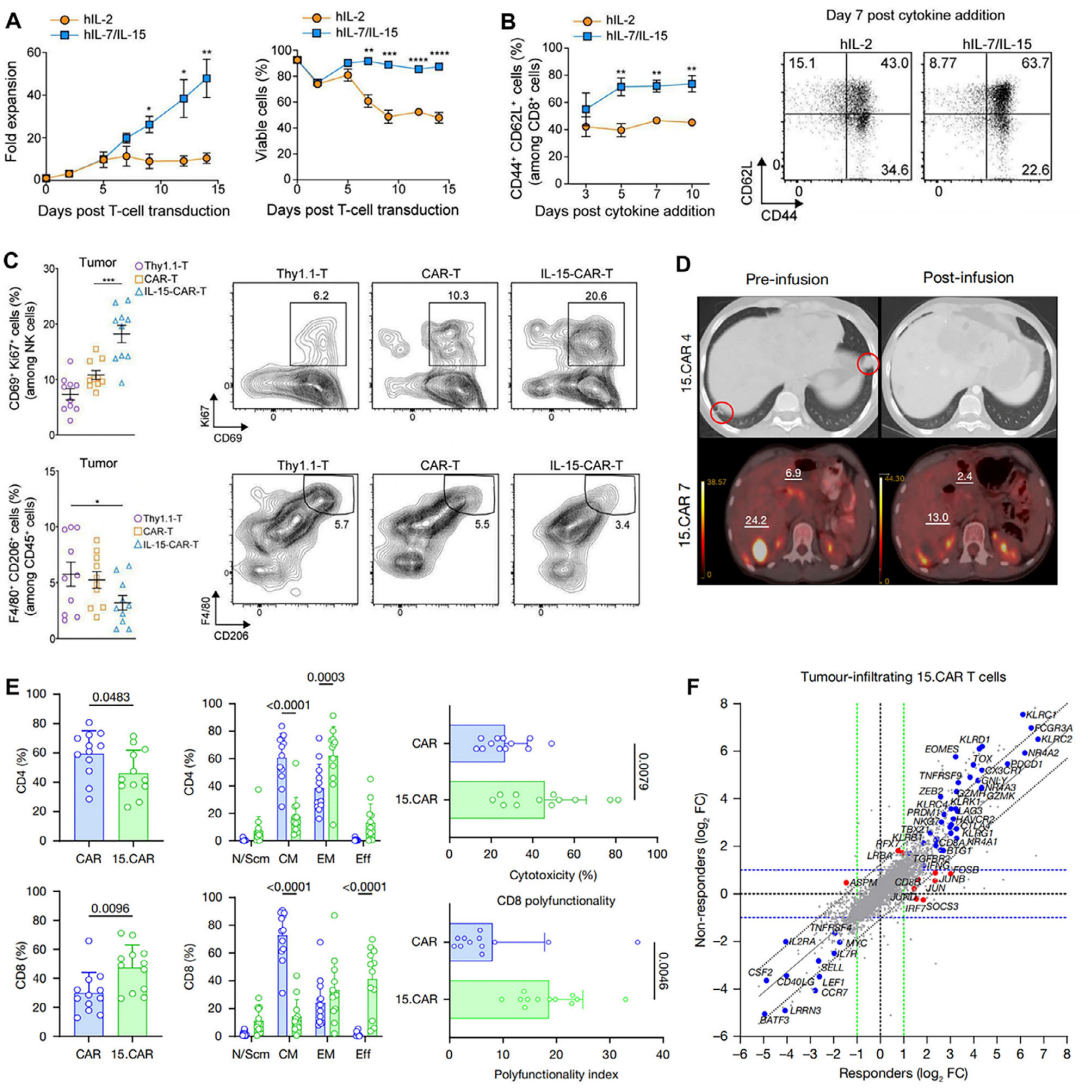
IL‐15 cytokine signaling assists improved CAR‐T therapy efficacy. (A) Cell proliferation after different treatments. (B) Quantitative analysis of CD44+CD62L+ in CD8+ cells. (C) Quantitative analysis and representative flow charts of CD69+Ki67+ cells and F4/80+CD206+ cells after different treatments. Figure 6A‐‐C is reprinted with permission from ref. [115], Copyright 2020, Rockefeller University Press. (D) Antitumor responses were determined by comparison of pre‐ and post‐infusion three‐dimensional imaging. (E) Frequencies of CD4/8 and effector/memory subsets, cytotoxicity analysis, and polyfunctionality index in the 15.CAR. (F) DEG comparison (product versus tumor‐infiltrating 15.CAR T cells) in responders (x axis) versus non‐responders (y axis) from the 15.CAR cohort (linear regression). Figure 6A‐‐C is reprinted with permission from ref. [122], Copyright 2025, Springer Nature.

Lentivirus, especially retrovirus, most commonly harnessed from the murine stem cell virus (MSCV), has been demonstrated as the most effective approach to stably transfect murine T cells. However, impaired completion of reverse transcription and limited nuclear import of the viral pre‐integration complex still hinder long‐term protein expression. Lanitis et al. optimized the application of retroviral vector into T cells, which introduced γ‐chain cytokines hIL‐7/IL‐15 as functional enhancers for improving CAR‐T cell proliferation and survival, as well as stimulating T_CM_ cell phenotype and preserving effector function [115]. Compared to hIL‐2 reached plateau with nine‐fold expansion, CAR‐T cells cultured with hIL‐7/IL‐15 appeared 26‐fold expansion at the same day 9 and maintained stable growth for 2 weeks with over 80% cell viability (Figure 6A). Furthermore, the population of CD8+ T cells showed a significant increase and differentiation to T_CM_ phenotype expressed positive CD62L and CD44 markers, which played a favorable role in elevating T cell persistence and antitumor capability (Figure 6B). After being engineered with CAR‐T cells to form IL‐15‐CAR‐T cells, the enhanced therapeutic effects in vivo could be attributed to increased population of CD69+ki67+ among NK cells (two‐fold) and decreased population of F4/80+CD206+ among macrophages (1.67‐fold), resulting in reversing ITME (Figure 6C).

Allogeneic Vγ9Vδ2 (Vδ2) T cells have garnered significant attention as promising candidates for cancer immunotherapy, owing to their demonstrated safety in allogeneic applications and intrinsic antitumor properties. The extensive body of research on αβ T cells, coupled with their clinical validation in autologous therapeutic products, has catalyzed substantial efforts toward developing allogeneic αβ CAR‐T cell therapies. To mitigate graft‐versus‐host disease (GvHD) risks associated with endogenous αβ T cell receptor (TCR)‐mediated recognition of mismatched MHC molecules, gene editing techniques are routinely implemented. Alternatively, the engineering of CAR‐modified antigen‐specific αβ T cells, exemplified by cytomegalovirus (CMV)‐specific T cell platforms, represents an alternative strategy for developing GvHD‐resistant allogeneic CAR‐T cell products [116]. Parallel investigative efforts focus on innate and innate‐like immune cell populations with inherently reduced alloreactive potential, including macrophages, NK cells, invariant iNKT cells, and γδ T cells. γδ T lymphocytes, characterized by TCRs comprising γ and δ chains, constitute a functionally distinct T cell subset accounting for merely 1–10% of circulating T cells, yet demonstrating evolutionarily conserved antimicrobial and antitumor activity [117]. These cells exhibit hybrid immunological features, integrating innate immune responsiveness through NK receptor signaling with adaptive immune capabilities mediated by TCR activation, while concurrently secreting context‐specific immunoregulatory cytokines [117, 118, 119]. The therapeutic potential of γδ T cells has been substantiated by clinical studies revealing that tumor‐infiltrating γδ T cell populations demonstrate superior prognostic significance compared to other immune cell subsets across diverse hematological malignancies and solid tumors. Furthermore, their non‐canonical MHC‐independent antigen recognition mechanism inherently eliminates GvHD risks, establishing γδ T cells as particularly suitable for allogeneic therapeutic applications in clinical oncology [120]. Yang et al. have developed a strategy that employs IL‐15‐fused CAR‐Vδ2 T cells from CD16 high (CD16Hi) donors. Flow cytometry analysis revealed that activated/expanded Vδ2 T cells with high CD16 expression exhibited significantly enhanced tumor cytotoxicity. Leveraging this finding, the authors engineered mesothelin (MSLN)‐targeting CAR and further introduced IL‐15, named as MCAR15‐Vδ2T. MCAR15‐Vδ2T cells demonstrated potent antitumor activity in vitro and in vivo, with superior tumor control in serial tumor rechallenge assays. Notably, H&E staining confirmed the absence of significant monocytic infiltration in MCAR15‐Vδ2T‐treated tumors, indicating effective mitigation of CRS typically associated with conventional CAR‐T therapies [121].

Heczey et al. have innovatively verified the evaluation in humans of the effects of IL‐15 co‐expression on GPC3‐expressing CAR T cells (hereafter GPC3 CAR T cells) [122]. Among patients with stable disease (SD), subject 15.CAR 4 and 15.CAR 10 exhibited a reduction in tumor burden exceeding 26%. Patient 15.CAR 7 demonstrated a decrease of approximately 12.8%, accompanied by a marked decline in positron emission tomography (PET) avidity within residual masses. This observation substantiates antitumor activity, thereby indicating therapeutic efficacy even in the absence of meeting formal Response Evaluation Criteria in Solid Tumors (RECIST) thresholds (Figure 6D). At the protein level, relative to conventional CAR T cell products, 15.CAR T cells exhibited a higher proportion of CD8+ T cell subsets alongside a marked reduction in central memory cell frequency. This shift correlated with expanded effector memory and terminally differentiated effector populations. Meanwhile, 15.CAR T cells demonstrated significantly enhanced cytolytic capacity and greater polyfunctional activity compared to standard CAR‐T cells, attributable to their elevated secretion of effector cytokines during target engagement (Figure 6E). The gene expression changes of 15.CAR T cells relative to baseline were evaluated during both the pre‐infusion and post‐infusion periods. In responders and non‐responders alike, genes linked to cytotoxic function, NK cell‐like transition, terminal effector differentiation, and exhaustion exhibited significant upregulation. Concurrently, genes associated with less differentiated naïve and memory T cell phenotypes demonstrated consistent downregulation in both cohorts (Figure 6F).

### 4.5 IL‐18 Cytokine Signaling

IL‐18 is a potent pro‐inflammatory cytokine, whose precursor molecule (pro‐IL‐18) is activated following cleavage by caspase‐1. Modification of therapeutic T cells with IL‐18 (i.e., “armoring” engineering of T cells) can enhance autocrine stimulation and positively regulate the ITME. Brandon et al. have engineered platforms for the constitutive secretion of the proinflammatory cytokine IL‐18 by chimeric antigen receptor T cells (CAR‐T cells). They initially developed CD19‐targeted IL‐18‐secreting CAR‐T cells (IL‐18 CAR‐T) to enhance CAR‐T cell activation and stimulate bystander immunity for treating B‐cell malignancies, with this approach demonstrating early clinical activity (NCT04684563). To address the challenge of insufficient CAR‐T cell activity against multiple myeloma with low antigen expression, the team further constructed: (1) single‐target CAR‐T cells secreting IL‐18 and targeting key tumor necrosis factor‐superfamily receptors for myeloma growth (BCMA, TACI, or BAFF‐R); and (2) dual‐target CAR‐T cells co‐expressing CARs specific for BAFF‐R and APRIL, combined with IL‐18 secretion capability. Utilizing a syngeneic immunocompetent mouse model of multiple myeloma, they demonstrated that engineered IL‐18 secretion enabled single‐target CAR‐T cells (e.g., targeting BCMA or BAFF‐R) to eliminate antigen‐low myeloma tumors. This efficacy was achieved through enhanced CAR‐T cell activation and the induction of type‐I/II interferon signaling in both T cells and myeloid cells, leading to the activation of tumoricidal macrophages. Furthermore, the BAFF‐R/APRIL dual‐target CAR design synergized with IL‐18 secretion, significantly amplifying cytotoxicity against myeloma exhibiting low expression of both BCMA and BAFF‐R (BCMA^low^BAFF‐R^low^), while also triggering an antigen‐dependent increase in IL‐18 production upon target recognition. This combinatorial strategy of engineered IL‐18 secretion and multi‐antigen targeting represents a rational approach to overcome antigen escape in myeloma by simultaneously boosting CAR‐T cell function and endogenous immune activation [123].

Wickman et al. engineered second‐generation CAR‐T cells targeting the C‐domain of oncofetal tenascin C (designated C.TNC‐CAR T cells), utilizing the G11 monoclonal antibody to recognize conserved human/murine epitopes for pediatric brain and solid tumor immunotherapy. To counter T‐cell dysfunction in antigen‐persistent solid tumor microenvironments, they integrated a constitutively active IL‐18 receptor into C.TNC‐CAR T cells via their modular leucine zipper receptor (ZipR) platform, bypassing endogenous cytokine production limitations. While baseline C.TNC‐CAR T cells mediated antigen‐specific cytolysis in vitro but showed limited efficacy in vivo, the engineered IL‐18 receptor, which sustained MyD88 signaling under chronic antigen exposure, synergistically bolstered T‐cell effector function, ultimately achieving potent tumor control in xenograft models through enhanced persistence and cytotoxicity [124].

Svoboda et al. engineered huCART19‐IL18, an autologous armored CAR T‐cell therapy targeting CD19 by genetically modifying T cells to constitutively secrete IL‐18, designed to circumvent resistance in relapsed/refractory CD19+ lymphomas where malignant cells retain antigen expression but microenvironmental suppression and T‐cell dysfunction drive treatment failure. This fourth‐generation construct incorporates multicomponent engineering: a humanized anti‐CD19 scFv domain to enhance persistence, a streamlined 3‐day manufacturing protocol preserving stem‐like T‐cell properties to limit exhaustion, and integrated IL‐18 secretion to reprogram immunosuppressive macrophages and amplify IFNγ production. Preclinical studies demonstrated synergistic efficacy with IL‐18 secretion, driving superior tumor control and prolonged survival in murine models by overcoming microenvironmental suppression [125].

To target CD371, an antigen expressed on AML blasts and leukemia‐initiating cells, Geyer et al. engineered CD371‐SAVVYζ‐IL18 CAR T cells, a next‐generation immunotherapeutic construct. Key design features include a fully human scFv, a modified CD28 co‐stimulatory domain to limit T‐cell exhaustion, wild‐type CD3ζ, and genetic engineering for constitutive IL‐18 secretion [126]. A Phase I trial (NCT06017258) assessed this armored CAR‐T therapy in relapsed/refractory acute myeloid leukemia (R/R AML) patients, a population with dismal prognoses. Although all patients had baseline CD371 expression, efficacy proved dose‐dependent: three heavily pretreated patients (all post‐allogeneic transplant) attained morphologic leukemia‐free state (MLFS) with MRD negativity within 15 days post‐infusion. This therapeutic response coincided with robust CAR‐T expansion and elevated IL‐18 levels (1.3–58.2 k pg mL^−1^). Of note, responders showed distinct immunophenotypic shifts: by day 14, CD8+ effector memory T cells dominated the CAR‐T population, acquiring cytotoxic markers (GZMB, PRF1) and NK‐like characteristics (CD56/CD16), alongside expansion of host NK cells with activated phenotypes. Treatment‐related toxicities included CRS (grade 3–4 in 2 of 5 patients) and prolonged cytopenias necessitating stem cell boosts. Mechanistically, IL‐18 secretion potentiated bidirectional immune activation, enhancing autocrine CAR‐T expansion and cytotoxic function.

GzB‐IL18, a protease‐redirected latent cytokine, was engineered by the research team through substitution of GzB cleavage sites for the native caspase‐1 recognition motifs in the IL‐18 precursor. This molecular design facilitates constitutive release of functionally inert pro‐IL18 from armored CAR T cells; cytokine activation occurs exclusively upon GzB secretion induced by target engagement during tumor cell killing. When incorporated into αβ or γδ CAR T platforms, GzB‐IL18 enhanced cytolytic activity, proliferation, and IFNγ production to levels matching constitutively active IL‐18. It further significantly improved antitumor efficacy by augmenting metabolic fitness and reprogramming myeloid cells in the tumor microenvironment. A key advantage is that while constitutive IL‐18 secretion unmasked cytokine release syndrome in immunocompetent models, the activation‐dependent GzB‐IL18 eliminated this toxicity while retaining therapeutic potency. This precise coupling of IL‐18 bioactivity to CAR T cell activation status confers a clinically desirable safety‐efficacy profile [127].

Lena et al. developed an IL‐18‐inducible GD2‐specific CAR‐T cell therapy [128]. GD2, as an embryonic glycolipid antigen, demonstrates significant abnormal cell surface expression in neuroblastoma (NBL) and diffuse midline glioma, while also exhibiting varying degrees of heterogeneous expression in various malignant tumors, including Ewing's sarcoma, osteosarcoma, breast cancer, and lung cancer. As one of the earliest tumor‐associated antigens used in CAR‐T cell therapy, GD2 has shown favorable safety profiles in multiple clinical trials, despite its baseline expression levels on neuronal cell bodies. This study employed a self‐developed self‐inactivating lentiviral vector system that achieves CAR activation‐dependent IL‐18 expression regulation through NFAT response elements. This inducible expression design offers dual advantages: (1) ensuring localized release and accumulation of IL‐18 in ITME; and (2) preventing potential risks associated with systemic immune activation. Preliminary proof‐of‐concept experiments have confirmed that compared with conventional GD2‐CAR‐T cells, this IL‐18‐enhanced construct significantly improves T cell effector functions. Currently, Lena et al. have completed the preclinical studies required for the first‐in‐human Phase I clinical trial (EU CT 2022‐501725‐21‐00) targeting relapsed/refractory GD2‐positive solid tumors (applicable to both pediatric and adult patients). By integrating the CAR signal‐driven IL18 inducible expression mechanism, the GD2‐IL18CART product provides an innovative solution to overcome challenges such as the immunosuppressive tumor microenvironment in solid tumor treatment [128].

### 4.6 IL‐23 Cytokine Signaling

IL‐23 is a cytokine that activates the STAT3 signaling pathway and is composed of two subunits: IL‐23A and IL‐12β. This cytokine is primarily secreted by activated macrophages and dendritic cells, and promotes the proliferation of memory T cells, particularly IL‐23 receptor (IL‐23R)‐expressing T helper 17 (TH17) cells [129]. Studies have demonstrated that incorporating the ICOS intracellular domain into CAR structures to drive TH17 phenotype differentiation of CAR‐T cells can significantly enhance their antitumor activity.

The IL‐23/IL‐23R signaling pathway plays a pivotal role in the pathogenesis of Crohn's disease (CD), a conclusion supported by substantial research evidence. Notably, genome‐wide association studies have revealed that loss‐of‐function mutations in the IL23R gene significantly reduce the risk of developing inflammatory bowel disease (IBD). Animal studies demonstrate that IL‐23 promotes intestinal inflammation and induces tissue damage, a pathological process that can be effectively inhibited by specific antibodies. Clinical investigations further confirm the therapeutic efficacy of IL‐12/23p40 or IL‐23p19 blockade in IBD patients. However, achieving long‐term disease remission requires establishing stable immune tolerance, a therapeutic goal that may not be attainable through cytokine blockade alone. To address this therapeutic challenge, Cui et al. innovatively designed an IL23R‐targeting CAR and successfully engineered regulatory T cells expressing this receptor (IL23R‐CAR Treg) [130]. Experimental data demonstrate: (1) This CAR construct exhibits minimal basal activation with excellent signal specificity; (2) While maintaining characteristic immunoregulatory phenotypes, IL23R‐CAR Tregs display antigen‐dependent immunosuppressive functions; (3) Most importantly, these engineered cells can selectively recognize and suppress abnormal activation of primary colon cells derived from CD patients. These findings not only validate IL23R‐CAR Tregs as a feasible novel therapeutic strategy for CD, but their unique activation signature also provides a potential biomarker system for subsequent clinical translation research.

### 4.7 Translation Boundaries in Solid Tumors

Although cytokine‐engineered CAR‐T cells have achieved breakthrough progress in reversing ITEM to enhance CAR‐T cell tumoricidal effects, there remain certain crucial limitations yet to be addressed.

First, on‐target off‐tumor toxicity (OTOT) represents a critical safety challenge in CAR‐T cell therapy, occurring when genetically engineered T cells recognize and attack healthy tissues that express the same target antigen as tumor cells, potentially leading to severe and sometimes life‐threatening adverse effects [131]. Upon binding to the target antigen, activated CAR‐T cells form specialized cytotoxic immune synapses with antigen‐positive cells, initiating a cascade of lytic mechanisms primarily mediated through the directed release of perforin and granzymes, which penetrate target cell membranes and induce apoptosis. Additional molecular pathways, such as FAS‐FAS ligand interactions triggering apoptosis, or substantial secretion of inflammatory cytokines including IFN‐γ and TNF, may further amplify immune activation and contribute to collateral tissue damage [132]. The development of safe and effective CAR‐T therapies for solid tumors is particularly dependent on rigorous antigen selection. Ideally, target antigens should be neoantigens, thus tumor‐exclusive proteins, derived from somatic mutations, aberrant post‐translational modifications (such as specific glycosylation patterns), or re‐expressed oncofetal antigens that are absent in normal adult tissues. However, such perfectly tumor‐specific antigens are clinically rare and often heterogeneous; for instance, the EGFRvIII mutation is found in only 24–67% of glioblastoma patients, limiting its broad applicability. As a result, most current CAR‐T products target tumor‐associated antigens (TAAs) such as EGFR, HER2, mesothelin, and GD2, which are overexpressed on tumors but still exhibit baseline expression in certain normal tissues, thereby elevating the risk of OTOT [133].

Second, the inevitable depletion of T‐cells constitutes a critical factor constraining the clinical efficacy of CAR‐T cell therapy. Continuous exposure of T cells to disease‐specific antigens and the ITME drives their differentiation into a dysfunctional state characterized by diminished proliferative capacity and effector function. Specifically, upon acute antigen stimulation, naïve CD8+ T cells (Tn) differentiate into effector T cells (Teff) [134]. During this differentiation, these cells undergo functional and metabolic reprogramming, forming antigen‐specific effector T cell populations through vigorous clonal expansion. The magnitude of this response is regulated by antigen density presentation; increased antigen density promotes T cell expansion. However, T cell sensitivity to antigen is inversely correlated with presentation density. Following antigen clearance, the majority of effector T cells undergo apoptosis. A minor population of surviving cells differentiates into memory T cells (Tmem), capable of persisting in the host without dependence on stimulatory antigens. Conversely, prolonged antigen exposure (e.g., to tumor antigens or chronic pathogens) induces sustained TCR signaling, triggering epigenetic reprogramming that forces cells into exhaustion differentiation pathways. This involves extensive alterations in DNA methylation, histone modifications, and chromatin accessibility, collectively locking T cells into a dysfunctional state. During this process, T cells progressively lose proliferative capacity and effector functions (including cytotoxicity and cytokine secretion, such as IFN‐γ and TNF‐α), while concurrently upregulating multiple inhibitory receptors (e.g., PD‐1, TIM‐3, LAG‐3, CTLA‐4), which further suppress immune responses. Within the tumor microenvironment, immunosuppressive factors (e.g., TGF‐β, adenosine, IL‐10) act synergistically with persistent antigen signaling to accelerate and intensify exhaustion programs by inhibiting TCR and cytokine signaling pathways. Crucially, even upon transfer to an antigen‐free environment, exhausted T cells fail to regain memory T cell characteristics or full functionality, confirming exhaustion as a terminal differentiation endpoint governed by stable transcriptional and epigenetic constraints [135, 136]. This process is particularly prominent in CAR‐T therapy: persistent tumor antigen exposure prompts CAR‐T cells to undergo exhaustion trajectories analogous to endogenous T cells, accompanied by metabolic alterations such as mitochondrial dysfunction and reduced oxidative phosphorylation, ultimately resulting in functional impairment and diminished tumor control efficacy. Although current research has established that cytokine‐armored CAR designs can effectively mitigate the inhibitory effects of the ITME to prevent T cell exhaustion, investigations specifically addressing sustained T cell exhaustion are still lacking owing to the limited duration of current dosing regimens and efficacy assessments, alongside inadequate long‐term monitoring of tumor recurrence dynamics [137].

Third, while demonstrating considerable therapeutic potential, CAR‐T cell immunotherapy remains burdened by significant treatment‐related toxicities, particularly CRS and ICANS [138]. Although absent in early murine models, these adverse effects emerged prominently in clinical translation. CRS typically manifests as a febrile prodrome accompanied by constitutional symptoms (rigors, neurological manifestations including ataxia, and anorexia), which may escalate into a life‐threatening systemic inflammatory cascade [139]. Progression to severe manifestations involves systemic inflammatory responses culminating in hemodynamic instability (hypotension), impaired tissue oxygenation (hypoxia), and multiorgan dysfunction affecting cardiovascular, pulmonary, and hepatic systems [140]. ICANS presents as a neurotoxic continuum ranging from subtle cognitive impairment (manifesting as dysphasia, executive dysfunction, and fine motor deficits) to potentially fatal neurological complications, including status epilepticus, cerebral edema‐induced coma, and motor paralysis. Notably, there are individuals exhibiting clinical signs of ICANS who also have pre‐existing CRS, with CRS often being considered the “initiating event” or cofactor for ICANS. Therefore, optimizing CAR‐T cell design is critical to balance the management of CRS and ICANS while enhancing cytokine‐mediated CAR‐T functionality [141].

## 5 Pathophysiology of CRS and ICANS

Current clinical applications of CAR‐T therapy predominantly target CD19+ hematological malignancies in treatment‐refractory populations, with toxicity management constituting a critical determinant of therapeutic success. These imperative underscores the necessity to elucidate the molecular underpinnings of CRS‐ICANS pathophysiology, particularly their intersection with systemic inflammatory pathways. Such mechanistic insights could inform next‐generation CAR constructs with enhanced safety profiles through tunable activation thresholds or built‐in cytokine modulation capabilities [142].

### 5.1 CRS in CAR‐T Therapy

The mechanistic progression of CRS can be delineated through five distinct phases [143, 144]: (i) Phase I: initiation & local activation. Following intravenous administration, CAR T‐cells migrate to tumor sites and engage antigen‐expressing targets through specific receptor‐ligand interactions; (ii) Phase II: amplified cytokine production. Clonal expansion of activated CAR T‐cells at tumor beds generates cytokine gradients (IFN‐γ, GM‐CSF) that recruit bystander immune cells, particularly monocytes/macrophages, establishing the CRS initiation microenvironment. (iii) Phase III: systemic inflammation. Circulating cytokine levels (IL‐6, IL‐1β) escalate exponentially, driving vascular endothelial activation through NF‐κB signaling. This manifests clinically as capillary leak syndrome (hypoalbuminemia >3 g dL^−1^), hemodynamic instability (MAP <65 mmHg), and multiorgan dysfunction via tissue hypoxia (SpO_2_<90%); (iv) Phase IV: neurovascular compromise. Disruption of blood‐brain barrier integrity by MMP‐9 facilitates CNS penetration of inflammatory mediators, precipitating ICANS through glutamate excitotoxicity and astrocyte activation; (v) Phase V: resolution & memory formation. Negative feedback mechanisms (PD‐1/PD‐L1 upregulation) induce CAR T‐cell apoptosis, while memory subsets persist (CD45RO+CD62L+ phenotype). Serum IL‐6 normalizes (≤ 7 pg mL^−1^) within 14–21 days post‐infusion in responsive patients. Early CD19 CAR‐T cell models failed to predict CRS due to species‐specific differences in IL‐6 receptor biology. The syndrome was first clinically characterized in the NCT02435849 trial (2015), where 94% of B‐ALL patients developed CRS (33%≥ grade 3). Current registry data (NCT03166878) show 41% ICANS co‐occurrence with severe CRS [136].

However, researchers have divergent opinions on the definition and grading scheme of CRS. In the Common Terminology Criteria for Adverse Events Version 3 (CTCAE v3) [145], which was in effect when many studies were initiated, CRS was defined as an event occurring within 24 h after the start of treatment, a criterion that is typical for CAR‐T cell therapy and other immune effector cell therapies. In CTCAE v4.03 [146], fever was not listed as a prerequisite for CRS, and the grading partially relied on whether the drug infusion was interrupted. This characteristic is not applicable to CAR‐T cells, which are typically administered as a single, short‐duration infusion (ranging from 2 to 30 min). In fact, CTCAE v4.03 is more suitable for grading toxicities associated with antibody infusions rather than cell infusions. In detail, Grade 1 CRS is defined as fever with a body temperature ≥38.0°C, which may be accompanied by or without constitutional symptoms such as myalgia, arthralgia, and malaise. These constitutional symptoms can be assessed according to CTCAE v5.0, but they do not affect the CRS grading. Currently, all proposed grading schemes have reached a basic consensus on the definition of Grade 1 CRS, yet not all of them list fever as a mandatory criterion. If the above symptoms occur concurrently with fever within the expected time frame, the condition is more likely to be diagnosed as CRS‐induced [147]. Grade 2 CRS is defined as fever complicated by hypotension that does not require vasopressors, and/or hypoxemia that requires oxygen therapy via low‐flow nasal cannula (≤6 Lmin^−1^) or blow‐by mask. Lee et al. [148]. once attempted to differentiate the grading of patients with different support needs. The original intention was to avoid the impact of early use of tocilizumab and corticosteroids on antitumor efficacy. However, retrospective analyses have shown that administering these drugs after CRS has fully manifested will not affect the therapeutic effect. At present, clinical practice tends to initiate anti‐cytokine therapy early, which can accelerate symptom relief without reducing efficacy. In addition, treatment may mask fever symptoms, while hypotension and hypoxemia usually take longer time to resolve. Grade 3 CRS is defined as fever complicated by hypotension requiring a single vasopressor (vasopressin may be used in combination), and/or hypoxemia requiring advanced oxygen therapy devices such as high‐flow nasal cannula (>6 L /min^−1^) or non‐rebreather mask, with the symptoms unexplainable by other causes. This grading is based on the type of vasopressors used (single vs. multiple types), and the use of vasopressin or milrinone is not taken into account. Previous grading methods based on the duration of vasopressor use or fraction of inspired oxygen (FiO_2_) were problematic due to cumbersome operation or unstable judgment. Now, the grading of hypoxemia is determined according to the type of oxygen therapy equipment, which is more practical. Due to the significant differences in hypoxemia judgment standards among different medical centers and patient groups, physicians are allowed to make flexible judgments based on the minimum level of oxygen therapy equipment required to correct hypoxia. Grade 4 CRS is defined as fever complicated by hypotension requiring multiple vasopressors (excluding vasopressin), and/or hypoxemia requiring non‐invasive or invasive positive pressure ventilation (such as CPAP, BiPAP, or intubation with mechanical ventilation), with the symptoms unexplainable by other causes. Regardless of the dosage, the combined use of multiple vasopressors is determined as Grade 4 CRS. When CRS progresses, pulmonary edema caused by capillary leakage can impair ventilation function, and patients requiring positive pressure ventilation support are also determined as Grade 4 CRS. It should be noted that intubation is performed only due to neurotoxicity‐induced impairment of airway protection ability without hypoxemia, or intubation due to seizures secondary to hypoxemia is not classified as Grade 4 CRS. If a patient remains intubated due to neurological diseases but other CRS symptoms have resolved, the diagnosis of CRS will no longer be made. The above risk factor assessment of CRS is summarized in Table 4.

**TABLE 4:** Risk factor assessment of CRS.

Published CRS grading systems	Grade 1	Grade 2	Grade 3	Grade 4
CTCAE version 4.03 [146]	Mild reaction: infusion‐related reactions, infusion interrupted, and supportive care indicated.	Therapy indicated: infusion interrupted; no improvement following symptomatic treatment; pharmacologic intervention indicated (antihistamine, NSAID, acetaminophen, IV fluids).	Persistent (e.g., not rapidly responsive) symptoms requiring pharmacologic intervention and recurrence of symptoms following initial improvement; hospitalization indicated.	Life‐threatening consequences: persistent or visual effects; urgent intervention required.
CTCAE version 5.0 [147]	Fever with or without constitutional symptoms.	Fever, hypotension (responding to fluids), Hypoxia (requiring ≤40% FiO_2_).	Fever, hypotension (requiring >40% FiO_2_).	Life‐threatening consequences: urgent intervention required.
Lee criteria [148]	Symptoms are life‐threatening: symptoms are mild, requiring only symptomatic treatment (e.g., antipyretics, fluids, analgesics, antiemetics).	Symptoms require intervention: Oxygen requirement: ≤40% FiO_2_; Hypotension responsive to fluids or low‐dose vasopressors (e.g., ≤10 mcg min^−1^ norepinephrine).	Symptoms require and respond to intervention: Oxygen requirement: >40% FiO_2_; Hypotension requiring high‐dose or multiple vasopressors; Grade 3 organ toxicity or Grade 4 transient.	Life‐threatening symptoms: Life‐threatening intervention required (e.g., mechanical ventilation); Grade 4 organ toxicity.
Penn criteria [149]	Mild reaction: Treated with supportive care; Mild organ toxicity (Grade 1).	Moderate reaction: Some degree of organ dysfunction (Grade 3) not related to CRS and/or attributed to other causes (e.g., infection); Hypotension (non‐CRS‐related symptoms, CRS‐related symptoms, including fever, myalgias, arthralgias, nausea, vomiting, fatigue, headache, rash, diarrhea).	More severe reaction: Hospitalization required for management, including Grade 4 LFTs or Grade 3 creatinine; Hypotension treated with multiple vasopressors; Hypoxia requiring supplemental oxygen (nasal cannula or face mask).	Life‐threatening complications such as intubation requiring mechanical ventilation.
MSKCC criteria [132]	Mild symptoms requiring observation or supportive care (e.g., antipyretics, analgesics, antiemetics).	Hypotension requiring vasopressors: ≤ 24 h; Hypoxia (supplemental oxygen, nasal cannula).	Hypotension requiring any vasopressors: >24 h; Hypoxia (supplemental oxygen, face mask or higher).	Life‐threatening symptoms: Hypotension refractory to high‐dose vasopressors; Hypoxia requiring mechanical ventilation.
CARTOX criteria [150]	Temperature ≥ 38°C; Grade 1 organ toxicity.	Hypotension responsive to fluids or low‐dose vasopressors; Hypoxia requiring ≤ 40% FiO_2_; Grade 2 organ toxicity.	Hypotension needing high‐dose or multiple vasopressors; Hypoxia requiring >40% FiO_2_; Grade 3 organ toxicity or Grade 4 transient.	Life‐threatening hypotension; Needing ventilator support; Grade 4 organ toxicity except transaminases.

Under multiple pathophysiological mechanisms, CAR‐T cell activation triggers the release of cytokines such as gamma interferon, granulocyte‐macrophage colony‐stimulating factor, and soluble inflammatory mediators to activate macrophages [151]. Macrophage activation is further enhanced by damage‐associated molecular patterns (DAMPs) released by septic tumor cells. Macrophage‐CAR‐T cell interactions exacerbate this process. Inflammatory mediators such as IL‐6 and IL‐1 secreted by activated macrophages play a key role in CRS pathology, which can lead to systemic complications and vascular leakage and amplify the overall inflammatory response [152]. Local chemokines attract monocytes and promote aggregation of activated macrophages, further exacerbating the inflammatory cycle. Two mouse models confirm that CRS is driven by a multicellular network of CAR‐T cells with host cells (core of macrophages and monocyte lineage cells). Xenograft models of CD19+ lymphoma show that IL‐6 is produced by dendritic cells, monocytes, and macrophages at the tumor site, with significantly higher macrophage secretion. Distal tumor‐free sites do not induce IL‐6 production, supporting the idea that CRS originates locally and triggers systemic pathology. Monocyte‐and macrophage‐derived IL‐1 is also an important driver of CRS toxicity [132].

Potential mechanisms of macrophage recruitment or activation during CRS are gradually being revealed. CAR T cells must be activated by myeloid cells to induce cytokine production. CD40‐CD40L interaction, although not required for CRS, enhances macrophage activation, facilitates IL‐6 production, and exacerbates CRS. The bi‐directional CD28‐CD80/CD86 signaling axis may play a key role in inducing IL‐6 secretion by myeloid cells, and is of great interest in studying the mechanism of CRS. The CD28‐CD80/CD86 signaling axis has been shown to play an important role in developing the CRS mechanism. CD28‐CD80/CD86 bi‐directional signaling axis may play a key role in inducing IL‐6 secretion by myeloid cells, an important concern in studying the CRS mechanism [153].

In terms of damage‐associated molecular patterns, CAR‐T cells are more inclined to induce cell death via an inflammatory programmed cell death mode, thermos‐apoptosis (rather than apoptosis), a process that results in the release of damage‐associated molecules, such as ATP and high‐mobility‐group protein B1 (HMGB1). Studies have shown that DAMPs released by tumor cells can induce macrophage activation in vitro. GM‐CSF is secreted by a variety of cells, including activated CAR‐T cells, and blockade of GM‐CSF inhibits the production of cytokines such as IL‐6 by monocytes in vitro [154]. TNF is a pleiotropic cytokine that activates immune cells to mediate inflammation and has direct tumor‐killing activity. Recent studies have suggested that TNF may be a potential factor in the induction of CRS by CD3/HER2 bispecific antibodies in the HER2 humanized mouse MMTV breast cancer model [155].

### 5.2 ICANS in CAR‐T Therapy

While the clinical manifestations of ICANS are well‐documented, the elucidation of its underlying pathophysiological mechanisms has yet to be fully achieved. Emerging evidence from recent animal model studies demonstrates that, beyond the established roles of pro‐inflammatory cytokines, pathological activation and subsequent injury of blood‐brain barrier (BBB) endothelial cells may directly induce neuronal damage, thereby contributing to the progression of ICANS [156]. Analogous to CRS, the pathogenic mechanisms underlying ICANS are postulated to originate from coordinated processes encompassing both CAR‐T cell‐mediated production of inflammatory cytokines (notably IL‐1β and TNF‐α) and paracrine‐mediated activation of resident immune cells‐predominantly macrophages‐within the tumor microenvironment [157]. Current research indicates that inflammatory mediators secreted by both CAR‐T cells and bone marrow‐derived stromal cells within the tumor microenvironment, including interleukin family members (IL‐1β, IL‐6, IL‐10), chemokines (CXCL8, CCL2), IFN‐γ, GM‐CSF, and TNF, enter systemic circulation through paracrine signaling. This cytokine storm initiates a pathological cascade culminating in BBB disruption, which facilitates three concurrent neurotoxic mechanisms: (1) enhanced CNS penetration of circulating cytokines and CAR‐T cells; (2) activation of resident microglial populations; and (3) amplified neuroinflammatory responses [158]. Experimental studies utilizing humanized NSG murine models have demonstrated that administration of human CD19‐specific CAR T cells incorporating either CD28 or 4‐1BB co‐stimulatory domains induces characteristic pathological sequelae, including impaired B‐cell regeneration, CRS, neurotoxic effects, and delayed‐onset lethal ICANS following initial CRS presentation [159]. In a translational non‐human primate ICANS model employing immunocompetent rhesus macaques, infusion of CD20‐targeted CAR T cells containing 4‐1BB co‐stimulatory domains precipitated ICANS symptomatology correlating with systemic cytokine elevation (particularly IL‐6) and encephalopathic changes following 8 days post‐infusion. Notably, this neurological manifestation temporally coincided with peak peripheral CAR T‐cell proliferation and upregulated expression of very late antigen‐4 (VLA4) integrin surface markers [160]. Crucially, this molecular alteration in adhesion molecule expression may facilitate CAR T‐cell transmigration across the BBB, thereby potentiating CNS infiltration and subsequent neuroinflammatory pathogenesis. Furthermore, in the whole pathophysiological progression of ICANS, there are typically associated characteristics, including enhanced vascular permeability, endothelial cell dysfunction, and neuroglial cell injury [161]. Clinical evidence demonstrates that ICANS patients exhibit elevated cerebrospinal fluid concentrations of total protein (14.5–19.3 mg dL^−1^), altered CD4+/CD8+ lymphocyte ratios (median 2.8 vs 1.4 in controls), and detectable CAR‐T cell populations (1.5–3.2% of total nucleated cells), indicative of BBB integrity compromise [162]. Notably, severe CRS and ICANS share coagulopathic manifestations, including hypofibrinogenemia (<150 mg dL^−1^) and elevated fibrin degradation products (>20 µg mL^−1^), which mirror disseminated intravascular coagulation (DIC) parameters observed in septic shock (OR 4.2, 95% CI 2.1–8.3). Mechanistic investigations of the ANG‐TIE2 signaling axis reveal that severe ICANS patients demonstrate significant elevations in serum biomarkers: ANG2/ANG1 ratio (median 3.8 vs 1.2, *p*<0.001), von Willebrand factor (vWF) levels (248% vs 98%, *p* = 0.003), and CXCL8 concentrations (156 pg mL^−1^ vs 32 pg mL^−1^, *p* = 0.007), collectively exacerbating endothelial activation and microvascular leakage (r = 0.82, *p* = 0.012). Post‐mortem neuropathological analyses in pediatric and young adult cohorts following CD19‐directed CAR‐T therapy demonstrate diffuse astrocytic degeneration (GFAP+ cell reduction 42±8%) and microglial activation (Iba1+ cell increase 3.2‐fold), confirming neuroglial pathology in ICANS pathogenesis [163].

In early clinical trials, researchers graded ICANS using the Common Terminology Criteria for Adverse Events Version 4.03 (CTCAE v4.03). Later, multiple oncology institutions across the United States that led CAR‐T cell clinical trials jointly issued the CARTOX criteria for grading ICANS in adults, thereby further refining the ICANS grading system. The CARTOX system assesses ICANS from multiple neurology‐related dimensions to determine its grade, and these dimensions cover all the symptoms and signs that may occur with grading ICANS (Table 5).

**TABLE 5:** Risk factor assessment of ICANS.

ICANS grading system	Grade 1	Grade 2	Grade 3	Grade 4
CTCAE v4.03 [146]	Encephalopathy (Immune Effector Cell‐Associated Encephalopathy) Mild symptoms: confusion, disorientation	Encephalopathy (Immune Effector Cell‐Associated Encephalopathy) Moderate symptoms: focal neurological deficit	Encephalopathy (Immune Effector Cell‐Associated Encephalopathy) Severe symptoms: prolonged or severe focal neurological deficit; requires intensive care	Encephalopathy (Immune Effector Cell‐Associated Encephalopathy) Life‐threatening consequences: urgent intervention required
	Seizure Benign partial seizure	Seizure Benign generalized seizure	Seizure New‐onset seizures: nonconvulsive status epilepticus	Seizure Life‐threatening consequences: urgent intervention required
	Dysphasia Awareness of error; occasional speech characteristics (e.g., paraphasic errors, word‐finding difficulty, not easily understood)	Dysphasia Moderate expressive deficit: speech characteristics (e.g., paraphasic errors, word‐finding difficulty, speaking in short sentences, easily understood)	Dysphasia Severe expressive or receptive deficit: requires adding text or visual cues to communication	
	Tremor Mild symptoms	Tremor Moderate symptoms: interfering with instrumental activities of daily living (IADL)	Tremor Severe symptoms: interfering with ADL	
	Headache Mild pain	Headache Moderate pain: IADL	Headache Severe pain: limiting self‐care ADL	Headache Life‐threatening consequences: urgent intervention required
	Confusion Mild disorientation	Confusion Moderate symptoms: focal neurological deficit	Confusion Severe symptoms: prolonged or severe focal neurological deficit; requires intensive care	Confusion Life‐threatening consequences: urgent intervention required
	Decreased Level of Consciousness Decreased level of consciousness	Decreased Level of Consciousness Sedation: low level of consciousness	Decreased Level of Consciousness Difficult to arouse: requires continuous stimulation	Decreased Level of Consciousness Life‐threatening consequences: urgent intervention required
	Cortical Oedema	Cortical Oedema	Cortical Oedema New‐onset moderate cerebral oedema; increased intracranial pressure	Cortical Oedema Life‐threatening consequences: urgent intervention required
CARTOX criteria [150]	Neurological Assessment (CARTOX‐10 Score) 0–1 (mild impairment)	Neurological Assessment (CARTOX‐10 Score) 2–4 (mild impairment)	Neurological Assessment (CARTOX‐10 Score) 5–6 (moderate impairment)	Neurological Assessment (CARTOX‐10 Score) ≥2 (severe impairment)
	Elevated ICP N/A	Elevated ICP N/A	Elevated ICP Stage 1–2: papilledema (Frisén scale) with normal cerebrospinal fluid opening pressure	Elevated ICP Stage 3–5: papilledema (Frisén scale) with increased cerebrospinal fluid opening pressure (>20)
	Seizure or motor weakness N/A	Seizure or motor weakness N/A	Seizure or motor weakness Partial seizure or focal weakness (transient)	Seizure or motor weakness Generalized seizure: generalized status epilepticus with continuous electroencephalographic seizures

The CARTOX neurotoxicity grading system also includes several other assessment dimensions, such as the patient's level of consciousness, motor symptoms, presence of seizures, and signs of increased intracranial pressure. When evaluating increased intracranial pressure and determining the grade of neurotoxicity, the guidelines recommend considering two aspects: first, whether the initial cerebrospinal fluid pressure is elevated; second, using the Frisén Scale to assess the severity of papilledema. Unfortunately, these testing methods are cumbersome to perform and lack sufficient accuracy, making them difficult to promote in routine clinical practice.

In terms of papilledema grading, different hospitals vary in their ability to conduct rapid assessments of this symptom. The grading results may also deviate due to factors such as the examining physician's personal experience and the use of fundus photography. For example, the same case may be graded as Frisén Grade 2 by one physician and Frisén Grade 3 by another. Lee et al. recommended the use of a slightly modified version of the CARTOX‐10 screening tool, which they renamed the Immune Effector Cell‐Associated Encephalopathy (ICE) Score [164]. The updated encephalopathy screening tool also adds an assessment item for “receptive aphasia” (inability to understand spoken language) in such patients. Its total score, ease of use, and scoring and grading criteria are all consistent with the original CARTOX‐10. It should be emphasized that the 10‐point ICE screening tool can only help physicians determine whether a patient has concurrent encephalopathy. The final grade of ICANS requires not only reference to the ICE score but also evaluation of other neurology‐related indicators, including the level of consciousness, motor symptoms, seizures, and signs of increased intracranial pressure or cerebral edema. These manifestations may occur together with encephalopathy or independently.

### 5.3 Biomarker Monitoring Strategies of CRS and ICANS

Researchers around the world have been striving to identify effective predictive biomarkers for CAR‐T cell therapy, but progress has remained limited so far. While the various existing predictive tools and indicators each have their own application scenarios, they all have obvious limitations. The Eastern Cooperative Oncology Group (ECOG) performance status is a general scale mainly used to assess disease progression and patients’ abilities to perform activities of daily living [165]. Tumor burden is a critical factor affecting the success of CAR‐T cell therapy; lower tumor burden and biomass are more conducive to CAR‐T cells exerting their antitumor effects. Some studies have also suggested that evaluating tumor burden prior to treatment may help predict treatment outcomes [166]. However, due to the stringent inclusion criteria for CAR‐T cell therapy and the complex mechanisms influencing treatment outcomes and the occurrence of adverse reactions, tumor burden alone cannot fully and effectively predict treatment progress. Meanwhile, clinical evidence has shown that patients who have received ≥ 3 prior lines of therapy tend to have a poorer prognosis, indicating that earlier administration of CAR‐T cell therapy may yield better therapeutic efficacy [167].

The CAR‐HEMATOTOX score is one of the commonly used predictive models, which incorporates baseline cytopenia indicators (such as thrombocytopenia, anemia, neutropenia, etc.) and inflammatory markers (such as C‐reactive protein, ferritin, etc.) [168]. All the indicators included in the score are associated with prolonged cytopenia following CAR‐T cell therapy. Although some studies have suggested that this score is easy to use and can assist in screening patients at high risk of hematotoxicity, the baseline CAR‐HEMATOTOX score alone has not been proven to accurately predict the overall progress of CAR‐T cell therapy [169]. The Inflammation‐Based Prognostic Score (IBPS) has been validated for assessing systemic immune‐inflammatory status and prognostic nutritional index, which may theoretically provide a reference for predicting CAR‐T therapy outcomes, but further research is needed to confirm its practical application value. [170]

The Endothelial Activation and Stress Index (EASIX score), a marker associated with endothelial damage, has been attempted to predict the risk of adverse reactions related to CAR‐T cell therapy. However, its core limitation lies in the use of surrogate blood biomarkers, including baseline levels of lactate dehydrogenase, creatinine, platelets, and, in some versions, C‐reactive protein and ferritin as well. These biomarkers cannot directly reflect endothelial damage but may also be linked to other pathological conditions, resulting in insufficient predictive specificity. The modified Cumulative Illness Rating Scale (CIRS) is mainly used to assess comorbidities in patients with hematologic malignancies. The comorbidities that exert the greatest impact on treatment prognosis have been categorized into four major groups, referred to as the “Severe4,” covering diseases of the respiratory, upper gastrointestinal, hepatic, and renal systems. Patients exhibiting a total CIRS score of ≥ 7 prior to CAR‐T cell therapy, which signifies the existence of severe or life‐threatening comorbidities, are prone to experiencing less favorable CAR‐T treatment progression and overall survival [171]. Nevertheless, since the number of patients with such severe underlying diseases is relatively small, this score is only applicable for distinguishing treatment responders from non‐responders among this small subset of critically ill patients, and has no reference value for patients without comorbidities or for identifying those at increased risk of developing severe adverse reactions. [172]

In addition, other studies have confirmed that specific single biomarkers, such as lactate dehydrogenase, PD‐1, ferritin, C‐reactive protein, IL‐6, and IL‐15, are statistically correlated with treatment progress prior to CAR‐T cell infusion [173]. However, these single indicators cannot fully capture the complex mechanisms underlying the body's immune response to CAR‐T cells and the antitumor effects of the cells. Overall, although some of the aforementioned predictive scores and biomarkers are associated with CAR‐T therapy outcomes and the risk of adverse reactions, none of them have been able to comprehensively reflect the complex interactions among various factors involved in the antitumor activity of infused CAR‐T cells and the body's immune system response. Therefore, there is an urgent need to develop more robust and comprehensive predictive scoring systems.

## 6 Current Strategies to Manage CRS and ICANS in CAR‐T Therapy

Toxicity represents a significant limitation for the clinical translation of CAR‐T cell therapy. Cytokines exhibit a dualistic role in immunotherapy; while capable of directly targeting tumor cells and reversing the ITME, thereby enhancing CAR‐T cell therapeutic efficacy, they conversely trigger CRS and ICANS, potentially life‐threatening toxicities mediated by systemic inflammatory cascades. Consequently, toxicity management has emerged as a critical research priority for future CAR‐T cell development. This chapter will focus on discussing strategies for managing CAR‐T cell‐associated toxicities, including orderly first‐ and second‐line clinical intervention, advanced logic‐gated circuits, autonomous neutralization of cytokines, and a reversible switching system. A clear summary of toxicity mitigation strategies will be outlined in Table 6.

**TABLE 6:** Toxicity mitigation strategies.

Category	Subtype	Mechanism	Reversibility	Readiness
Clinical management	Tocilizumab	IL‐6 receptor blockade; alleviates CRS symptoms without impairing CAR‐T efficacy	Yes	FDA‐approved first‐line CRS treatment
Clinical management	Siltuximab	Neutralizes IL‐6 but does not increase CSF IL‐6 levels	Yes	Clinical use reported in severe CRS/ICANS cases
Clinical management	Anakinra	IL‐1 receptor blockade and able to penetrate BBB	Yes	Clinically used but under clinical evaluation
Clinical management	Lenzilumab	Neutralizes GM‐CSF and reduces inflammatory markers and CNS immune infiltration	Yes	Under clinical evaluation
Clinical management	Emapalumab	Neutralizes IFN‐γ	Yes	Under clinical evaluation
Clinical management	Corticosteroids	Broad anti‐inflammatory effects via cytokine suppression and transcriptional regulation	Yes	Guideline‐directed
Clinical management	Metoprolol	Inhibits IL‐6 protein translation in monocytes and reduces CRS severity without impairing CAR‐T	Yes	Exists clinical trial evidence in treating CRS
Clinical management	Natalizumab	VLA‐4 blockade limits CAR‐T and myeloid cell CNS infiltration	Yes	Experimental
Logic‐gated CAR‐T cells	AND‐logic activation	Require simultaneous recognition of two targeting signals to achieve full CAR activation, reducing off‐tumor activation and CRS.	Yes	Approval
Logic‐gated CAR‐T cells	ITME‐sensitive IF/THEN logic (IF‐THEN‐logic activation)	Recognize tumor‐specific features to conditionally permit CAR signaling only in diseased tissues.	Yes	Phase I
Logic‐gated CAR‐T cells	Exogenous‐logic circuits: ON/Off‐switch zipper	Splits antigen recognition from signaling, so that soluble adaptor molecules externally bridge universal CARs to selected antigens	Yes	Phase I
Logic‐gated CAR‐T cells	NOT‐logic inhibition	Couple a stimulatory CAR with an inhibitory counterpart that delivers potent suppressive signals upon detecting NOT‐malignant cell markers	Yes	Phase I/II
Autonomous cytokine neutralization	Secretion of soluble antagonists	Release cytokine blockers to neutralize inflammatory mediators.	Yes	Phase II
Autonomous cytokine neutralization	Receptor‐based neutralization	Conjugate scFv derived from an anti‐IL‐6 monoclonal antibody to a TM anchoring domain	Yes	Preclinical only
Switch systems	Suicide genes	Incorporate inducible apoptotic triggers to enable desired T‐cell elimination	No	Phase I
Switch systems	Reversible switches‐CAR kinase inhibitors	Specifically obstructing phosphorylation of immune receptor tyrosine‐based activation motifs within T‐cell activation‐associated proteins	Yes	Phase I
Switch systems	Reversible switches‐anti‐GD2 CAR‐T cells engineered with ligand‐induced degradation domains	Fuse a ligand‐inducible degradation domain to the CAR so that addition of a small‐molecule ligand triggers rapid proteasomal degradation of the CAR‐LID fusion, while ligand withdrawal allows CAR re‐expression	Yes	Preclinical only
Switch systems	Reversible switches‐proteolysis‐targeting chimera compounds	Introduce PROTAC compounds to enable reversible regulation of CAR‐T cells through CAR protein degradation	Yes	Preclinical only
Switch systems	Reversible switches‐hypoxia‐sensing CARs	Place CAR under the dual regulation of the HIF‐1α/HRE‐driven hypoxia‐inducible promoter and ODD‐mediated oxygen‐dependent degradation, ensuring its stable expression and cytotoxic function exclusively in the hypoxic tumor microenvironment.	Yes	Phase I

### 6.1 Orderly First‐ and Second‐Line Intervention Options

Supportive care remains the cornerstone of CRS management. Initial interventions for grade 1–2 CRS include antipyretic therapy, intravenous fluid replacement, oxygen therapy support, vasopressor administration for persistent hypotension, and prophylactic antibiotic treatment if infection cannot be ruled out [174]. Notably, the clinical boundary between CRS and sepsis is often blurred, and clinicians must strike a careful balance between early immunosuppressive therapy and the risk of masking or exacerbating underlying infections. In the field of cytokine‐targeted therapy, tocilizumab, the only IL‐6 receptor antagonist approved by the FDA for CRS treatment, exerts its therapeutic effects by blocking the IL‐6 signaling pathway. It can effectively alleviate fever, hypotension, and capillary leakage without compromising the efficacy of CAR‐T cell therapy [175]. In most cases, 1–2 doses are sufficient to reverse grade ≥2 CRS, and its rapid onset of action renders it the first‐line agent in emergency settings. Anakinra, an IL‐1 receptor antagonist, is now recognized as an effective adjunctive therapy for both CRS and ICANS, especially for patients refractory to tocilizumab or those with concurrent neurotoxicity, owing to its ability to readily cross the BBB [175]. Lenzilumab and emapalumab, which target GM‐CSF and IFN‐γ, respectively, are currently under clinical evaluation for the management of steroid‐refractory or severe CRS, and both agents have demonstrated promising potential in reducing inflammatory marker levels and restoring hemodynamic stability. In addition, in vitro experiments have confirmed that metoprolol can directly inhibit IL‐6 translation in human monocytes, while clinical trial data have indicated that it can significantly reduce the severity of CAR‐T cell‐induced CRS without impairing CAR‐T cell function [176]. These findings not only provide a solid scientific basis for repurposing metoprolol as a CRS therapeutic agent but also identify IL‐6 protein translation as a novel clinically relevant target for anti‐inflammatory drug development. Corticosteroids (e.g., dexamethasone and methylprednisolone) serve as a crucial second‐line treatment option for CRS patients unresponsive to IL‐6 receptor blockade or those with concurrent ICANS. Although previous studies raised concerns about their potential to inhibit CAR‐T cell proliferation, recent research has shown that early administration of moderate‐dose corticosteroids for a short duration does not significantly compromise antitumor efficacy [177]. Its recommended indications include grade ≥3 CRS refractory to tocilizumab, CRS complicated with neurological symptoms, life‐threatening organ dysfunction, and immune effector cell‐associated hemophagocytic lymphohistiocytosis (IEC‐HS) and other severe inflammatory syndromes. Clinical dosing regimens vary across institutions; the commonly used protocols involve intravenous infusion of dexamethasone (10 mg every 6 h) or methylprednisolone (1–2 mg/kg/day), with a gradual tapering of the dosage over 3–5 days based on the patient's clinical response [178].

The consensus from the American Society for Transplantation and Cellular Therapy (ASTCT) clearly states that the management of ICANS should be based on disease grading: supportive measures such as intravenous fluid infusion, aspiration support, and prophylactic anti‐seizure therapy are recommended for mild ICANS, while high‐dose corticosteroids or biological agents like siltuximab and anakinra should be considered for severe cases [179]. Potential biological agents with relevant mechanisms of action and CAR‐T cell modulation strategies represent important promising directions for the treatment or prevention of ICANS.

Corticosteroids exert potent anti‐inflammatory effects through rapid nongenomic regulation and gene transcriptional modulation, which can balance the production of pro‐inflammatory and anti‐inflammatory mediators, block cytokine signaling and adhesion molecule interactions, and induce apoptosis [180]. They are effective in alleviating severe CRS and ICANS, but their use requires caution because early studies have found that high‐dose or long‐term administration can lead to impaired CAR‐T cell function. A retrospective study involving 100 patients with large B‐cell lymphoma further confirmed that higher doses, earlier initiation, or longer durations of corticosteroid use were associated with significantly shorter progression‐free survival and overall survival [181].

In the field of biological agents, siltuximab, an IL‐6 signaling pathway inhibitor and IL‐6 neutralizing antibody, differs in mechanism of action from tocilizumab. Intravenous administration of siltuximab does not increase IL‐6 concentrations in the cerebrospinal fluid, making it suitable for the simultaneous treatment of CRS and ICANS [182]. Anakinra, an IL‐1 receptor antagonist, can cross the BBB to enter the CNS and is indicated for various autoimmune inflammatory diseases. Preclinical studies have shown that anakinra is more effective than tocilizumab in ameliorating severe neurotoxicity without impairing CAR‐T cell function, and multiple clinical trials are currently evaluating its combination with CAR‐T cell therapy [183]. Among other cytokine‐targeted therapies, lenzilumab, an anti‐GM‐CSF antibody, reduced the infiltration of CAR‐T cells and myeloid cells into the CNS, alleviated neurotoxic symptoms, and prolonged survival without compromising CAR‐T cell function in preclinical studies. Additionally, therapeutic strategies targeting adhesion molecules are under development. Since CAR‐T cells express higher levels of integrin α4β1 (VLA‐4) than non‐CAR T cells, blocking the VLA‐4 pathway with natalizumab can prevent CAR‐T cell infiltration into the CNS and thereby reduce CNS inflammation [152, 184].

### 6.2 Logic‐Gated CAR T Cells

#### 6.2.1 Activating CAR‐T via And‐Logic Circuits

**FIGURE 7:**
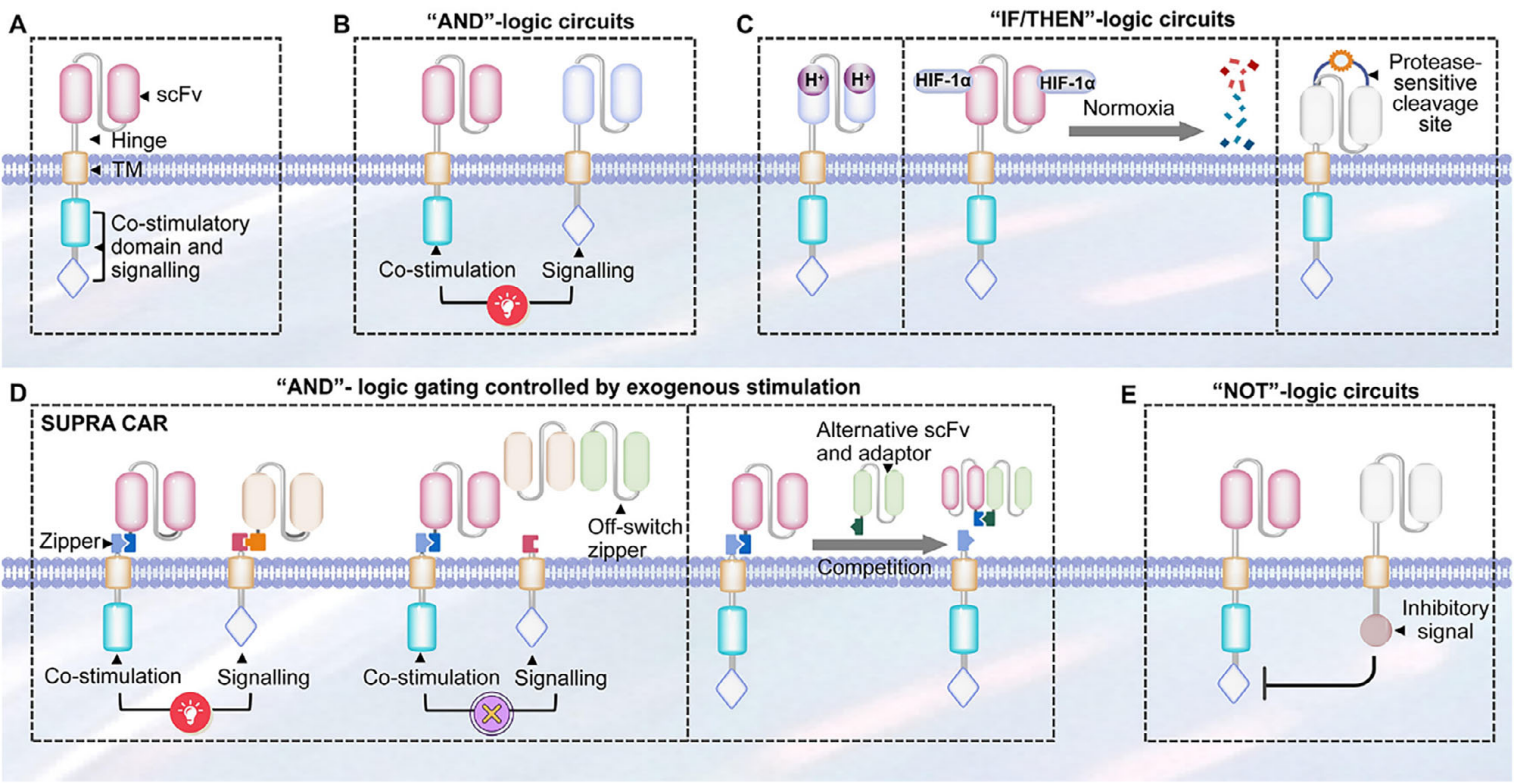
Examples of logic‐gating strategies tested in CAR T cells. (A) Conventional structure of the second‐generation CAR‐ T cells. (B) Dual AND‐logic CAR‐T cells accomplish signaling and co‐stimulation via two independent receptors. (C) IF/THEN‐logic CAR‐T cells utilize the unique tumor microenvironment. (D) AND‐logic gating controlled by exogenous stimulation, including a typical Zipper structure. (E) NOT‐logic CAR‐T cells utilize an inhibitory signal.

From second‐generation CAR‐T, the co‐stimulating domain and signaling domain are concatenated to trigger a potent T cell immune response (Figure 7A). However, the initial activation of CAR‐T and other endogenous immune cells could bring augmented systemic concentration of pro‐inflammatory cytokines, resulting in serious toxic side effects, including CRS. Engineering AND‐logic circuits that optimally co‐recognize multiple antigens expressed on the targeting malignant cells provides an example to overcome the above issues. Typically, AND‐logic CAR‐T cells express two distinct structures, one of which contains a signaling domain (like CD3ζ) and the other contains a co‐stimulating domain (like CD28 or 4‐1BB) (Figure 7B) [185]. Enabling complete CAR‐T activation, two scFv‐targeted TAAs are required to be expressed on the foci tissues, rather than a single TAA connection, which would reduce signaling to circumvent CRS induced by off‐target effects. AND‐logic‐based CAR‐T cells have exhibited optimized therapeutic effects through targeting CEA and mesothelin, while single‐CEA‐positive or mesothelin‐positive cells disappeared CAR‐T activating capability. In addition to dual‐TAAs management, other specific tumor‐related traits are also available for AND‐logic circuits designs [186]. For example, high‐expression signal factors, IL‐4 and TGF‐β, are introduced to PSCA‐CAR‐T cells to enhance potency and safety in tumor‐bearing mice models. Unfortunately, potential leakiness of CAR should always be taken into consideration, appealing for more intelligent logic‐system development.

#### 6.2.2 Activating CAR‐T via ITME‐Sensitive IF/THEN‐Logic Circuits

ITME‐responder systems have been introduced to precise CAR‐T structure design for reducing the risk of leakiness, which formed IF/THEN‐logic circuits (Figure 7C). pH‐sensitive CAR is a typical approach capitalizing on the Warburg effect, characterized by an obvious acidic intratumoral environment in tumors owing to accelerated glycolysis and enhanced lactate accumulation. With the pH‐limited CAR binding region, antigen‐targeting CAR‐T preferentially detected antigens in acidic ITME, which could avoid unnecessary connection between CAR‐T and non‐malignant tissues and ensure effective tumor regression [187]. Hypoxia response is another property that can be utilized to restrict CAR‐T behavior in ITME. Conceptually, the hypoxia‐sensing subdomain of HIF‐1α was fused to the intracellular C‐terminal end of a multichain anti‐CD19 CAR. Under normoxia, HIF‐1α‐fused CAR is degraded. Conversely, under hypoxic conditions in the ITME, CAR would be expressed smoothly locally with non‐HIF‐1α‐degradation. Kosti, P. et al. have developed a novel HIF‐1α‐modified pan‐anti‐ErbB CAR, possessing strict CAR‐T expression only under the hypoxia (0.1% oxygen), leading to great tumor control in the ovarian adenocarcinoma‐bearing mouse without significant CRS or other toxicity [188]. Anti‐EGFR CAR is masked by a protease‐sensitive cleavage site, providing a novel methodology of “IF/THEN”‐logic circuits. CAR emerges expose when a protease‐sensitive cleavage site is identified and disconnected by a masking peptide, inducing normal CAR function commission, and the function is only limited to ITME. Membrane‐type serine protease 1 (MT‐SP1), urokinase‐type plasminogen activator (uPA), and the lysosomal enzyme legumain have been recognized as reliable response sites owing to their overexpression in the ITME [189]. However, there is still a crucial question that should be considered that typical ITME features, such as acidic pH, hypoxia, and enzyme sensitivity, also occurred in non‐malignant tissues, resulting in varying degrees of systemic toxicity induced by ITME‐based CAR‐T cells activation in locations distant from tumor foci. Therefore, focusing both on the targeting antigens over‐expressed in the tumors, and ITME features is of great importance.

#### 6.2.3 Activating CAR‐T via Exogenous‐Logic Circuits

Split, universal, and programmable (termed SUPRA) CAR‐T cell enables the activation of CAR‐T controlled under the exogenous logic circuits, which expands the regulation scope to after administration. There are two classical strategies to design SUPRA CAR‐T cells as follows: (1) “ON/Off‐switch zipper” and (2) “Alternative zipper adaptor” (Figure 7D). Two parts are included in each type of SUPRA CAR‐T cells: (1) the TM and endodomain of a CAR connected to an extracellular leucine zipper, and (2) a soluble scFv attached to a leucine adaptor (adaptor‐scFv). When the scFv connects with CAR via zipper structure, CAR‐T cell shares complete activation [190]. In the “ON/Off‐switch zipper,” “AND”‐logic gating is introduced to achieve CD3ζ signaling and co‐stimulation independent delivery by two existences of SUPRA CARs. The utilization of adaptor‐scFv platform offers unique flexibility for dynamically redirecting CAR specificity in vivo through combinatorial targeting of TAAs. To mitigate off‐target activation, engineered soluble zipper scFvs (zipFvs) can competitively block adapter scFv‐antigen engagement, effectively decoupling CAR‐T cell signaling. This modular design circumvents the need for recurrent CAR‐T cell reengineering, as multi‐antigen targeting is achieved by simply reprogramming zipFvs with distinct scFv payloads [182]. By employing orthogonal leucine zipper pairs in both zipFv and zipCAR components, we establish a tightly controlled activation logic governed by combinatorial antigen recognition. The system permits on‐demand target switching through sequential administration of alternate zipFv‐scFv conjugates. Three critical parameters dictate system performance: scFv‐TAA interaction kinetics, ZipFv concentration gradients, and ZipCAR expression levels. These variables collectively determine CAR activation thresholds and cytokine production dynamics. Notably, administration of high‐affinity zipFv variants can competitively displace existing zipFv‐zipCAR interactions, effectively suppressing hyperactivated CAR‐T cell responses through dominant‐negative inhibition [191].

#### 6.2.4 Activating CAR‐T via NOT‐Logical Circuits

AND‐logic and IF/THEN‐logic circuit architectures simultaneously engage CAR activation and target recognition to trigger cytotoxic responses. In contrast, NOT‐logical gating strategies couple a stimulatory CAR with an inhibitory counterpart (iCAR) that delivers potent suppressive signals upon detecting NOT‐malignant cell markers (Figure 7E) [192]. The first validation using PSMA‐targeted iCARs in xenograft models has demonstrated selective attack of PSMA+/CD19+ NALM6 cells while alleviating cytotoxicity against PSMA‐ targets. This approach effectively mitigates OTOT, as further evidenced by anti‐CD93 CAR/anti‐CD19 iCAR systems in parallel studies. Notably, an alternative NOT‐gate mechanism has been characterized that employs synNotch receptors to induce CAR‐T apoptosis through truncated BH3‐interacting domain death agonist (tBID) expression when recognizing non‐malignant tissue antigens. Crucially, maintaining equilibrium between peripheral T‐cell depletion and on‐tumor prosperity emerges as a pivotal factor for successful clinical implementation of CAR‐T therapies.

### 6.3 Autonomous Neutralization of Cytokines

As previously discussed, CAR‐T cell expansion is inherently associated with the release of inflammatory cytokines, a process that subsequently induces CRS. Synchronizing inflammation inhibitors with CAR‐T cell proliferation enhancement may represent a reliable therapeutic strategy to mitigate this limitation. Here, two established clinical approaches, including engineered secretion of soluble cytokine antagonists and receptor‐mediated cytokine neutralization systems, are summarized. This paradigm shift toward self‐limiting CRS control redefines therapeutic windows, which achieves autonomous CRS inhibition without interrupting CAR‐T therapy function.

#### 6.3.1 Engineered Secretion of Soluble Cytokine Antagonists

IL‐1 emerges as the central pathogenic mediator of CRS, with its NF‐κB‐driven feedforward loop amplifying interleukin‐6/IFN‐γ production (>8‐fold in murine models; *p* < 0.001) and directly correlating with life‐threatening hypotension in 62% of anti‐CD19 CAR‐T recipients. Pharmacological blockade of IL‐1R not only abrogates this cytokine storm cascade but also preserves CAR‐T cytotoxicity, resolving the critical efficacy‐toxicity trade‐off. Giavridis et al. engineered CAR T cells to constitutively produce IL‐1 receptor antagonist (IL1RN/IL‐1Ra) [193]. Systemic anakinra delivery achieved 100% survival versus 40% in controls (*p* < 0.0001) in a xenogeneic CRS model (NSG mice with human PBMC reconstitution). Strikingly, IL‐1Ra‐armored CAR‐T cells recapitulated this protection (93% survival; *p*<0.001) while maintaining full cytolytic capacity and preserving cytokine output with obvious IL‐6 secretion decrease. This self‐regulating circuit resolves the efficacy‐toxicity paradox plaguing current CAR‐T therapies, establishing a paradigm for next‐generation autonomous cellular therapeutics. Correspondingly, Xue et al. conducted parallel phase I/II trials (ChiCTR2000031868/ChiCTR2000032124) evaluating multiple‐functional armored CAR‐T cells co‐expressing anti‐CD19 or anti‐BCMA with anti‐IL‐6 (form Sirukumab). Longitudinal cytokine profiling revealed IL‐1Ra serum levels positively correlated with CAR‐T expansion kinetics, while the classic IFN‐γ/IL‐6 coupling was decoupled in 16/18 patients (89%). Notably, IL‐1β remained subthreshold (<5 pg mL^−1^) throughout treatment, contrasting with historical controls (peak 48 ± 12 pg mL^−1^). CRS grading per ASTCT consensus criteria showed 14 patients (78%) developed only grade 1–2 symptoms, eliminating the need for tocilizumab rescue in 83% of cases (vs 35% in standard‐of‐care cohorts). Remarkably, this safety enhancement coexisted with undiminished efficacy that displayed complete remission in 90% AAL, 40% lymphoma, and 100% multiple myeloma [194].

The above findings establish a biomarker‐driven engineering framework that decouples therapeutic potency from cytokine‐mediated toxicity, paving the way for universal armored CAR architectures across hematologic malignancies. However, mono‐armed‐CAR‐T cells may not completely overcome the CRS risk. Notably, there are 22% (4/18) patients in the treatment of anti‐IL‐6/IL‐1Ra armored CAR‐T recipients who have developed grade 3 CRS despite dual cytokine blockade. These findings underscore the need for further investigation into complementary therapeutic targets [172]. Notably, Yi et al. recently conducted a phase I clinical trial employing CRISPR‐edited CAR‐T cells with GM‐CSF knockout and additional genetic modifications. Despite the limited sample size, this combined approach demonstrated potential for mitigating CRS risk, as no grade ≥ 3 CRS events were observed during the study period. These preliminary results warrant validation through larger‐scale clinical trials [195].

#### 6.3.2 Receptor‐Based Neutralization

Tan et al. recently developed a novel membrane‐bound anti‐IL‐6 receptor (mbaIL6R) engineered CAR‐T platform, wherein scFv derived from an anti‐IL‐6 monoclonal antibody was conjugated to a TM anchoring domain. This innovative design demonstrated dual functionality, including effective neutralization of both exogenous human IL‐6 and monocyte‐derived IL‐6 (as evidenced by in vitro co‐culture with THP‐1 cells), as well as preserved antitumor potency through enhanced in vivo interferon‐gamma (IFN‐γ) production (*p *< 0.01 vs. controls) and improved CAR‐T proliferative capacity. Flow cytometric analysis revealed a 1.8‐fold increase in central memory T cell populations with concomitant upregulation of effector memory markers. Transcriptomic profiling further identified sustained expression of immune checkpoint regulators PD‐1 (2.3‐fold), TIM‐3 (1.7‐fold), and LAG‐3 (1.5‐fold) in mbaIL6R‐CAR‐T cells compared to conventional counterparts. These findings propose a paradigm‐shifting strategy for CRS mitigation while maintaining therapeutic efficacy, though clinical validation remains warranted [196].

### 6.4 Switch Systems

Substantial progress has been made in engineering genetically encoded biosafety architectures into CAR‐T cell therapies, enabling preemptive toxicity termination and mitigation of cytokine overactivation. This section highlights critical safety mechanisms and design concepts exemplified by switch systems, which demonstrate precise spatiotemporal control over therapeutic cell populations.

#### 6.4.1 Suicide Genes

The suicide gene system has employed precision control systems through incorporating inducible apoptotic triggers, including inducible caspase 9 (iCasp9) or herpes simplex virus tyrosine kinase (HSV‐TK), to enable desired T‐cell elimination [175]. The HSV‐TK prototype demonstrates a phosphorylation‐dependent mechanism: viral thymidine kinase converts ganciclovir into cytotoxic triphosphate derivatives, inducing DNA chain termination and selective ablation of transduced cells. Clinical applications in hematopoietic stem cell transplantation reveal dose‐dependent mitigation of graft vs. host disease (GVHD) severity and CRS‐associated cytokine storms through CAR‐T/HSC control. However, HSV‐TK immunogenicity compromises cellular persistence, necessitating alternative approaches. The iCasp9 platform addresses these limitations through a synthetic caspase‐9/FKBP(F36V) chimera. Pharmacologic dimerizers (AP1903/AP20187) trigger caspase‐3‐mediated apoptosis cascades, achieving >90% transduction efficiency in target populations while preserving antitumor functionality (e.g., cytokine secretion capacity) [197]. Preclinical models demonstrate AP1903‐activated iCasp9 systems effectively resolve CRS pathophysiology, evidenced by IL‐6 suppression (82% reduction), weight stabilization, and GVHD prophylaxis without impairing therapeutic efficacy. Antibody‐mediated suicide switches represent another clinically translatable strategy leveraging existing therapeutic monoclonal antibodies (mAbs) for precision control of engineered cell therapies. This approach engineers CAR‐T cells to express surface neoepitopes compatible with FDA‐approved mAbs, enabling rapid therapeutic cell depletion via a ADCC. Certain research groups have integrated the utilization of FDA‐approved monoclonal antibodies to encourage the lysis of CAR‐T cells expressing the targeted antigens. In the Cetuximab/tEGFR system, CAR‐T cells expressing truncated EGFR (tEGFRΔL15) show 98.7±0.9% depletion efficiency (*p *< 0.001) within 24 h post‐cetuximab administration in NSG mouse models, while maintaining >80% target cell killing capacity pre‐depletion [198]. Additionally, CD52‐directed clearance has been considered as an alternative approach that Alemtuzumab‐mediated ablation achieves a reduction of CD52+ CAR‐T cells without compromising hematopoietic stem cell viability. Incorporation of a synthetic rituximab epitope (KDR*: Lys185Asp/Arg190Pro) into CAR constructs enables selective CD20+ B‐cell sparing (15% depletion) while eliminating 99.2% transduced CAR‐T cells in humanized mouse models. However, a critical consideration lies in ensuring that biomarkers expressed on CAR‐T cells do not adversely affect healthy cells or other lymphocytes. Since the CD20 antigen naturally occurs on B cells, rituximab‐mediated depletion of CD20‐expressing cells may inadvertently eliminate both B cells and engineered CAR‐T cells. Furthermore, if full‐length CD20 receptors are incorporated or epitopes are engineered based on CD20‐specific antibody targets, this configuration could potentially compromise the therapeutic immune response [199, 200].

#### 6.4.2 Reversible Switch

**FIGURE 8:**
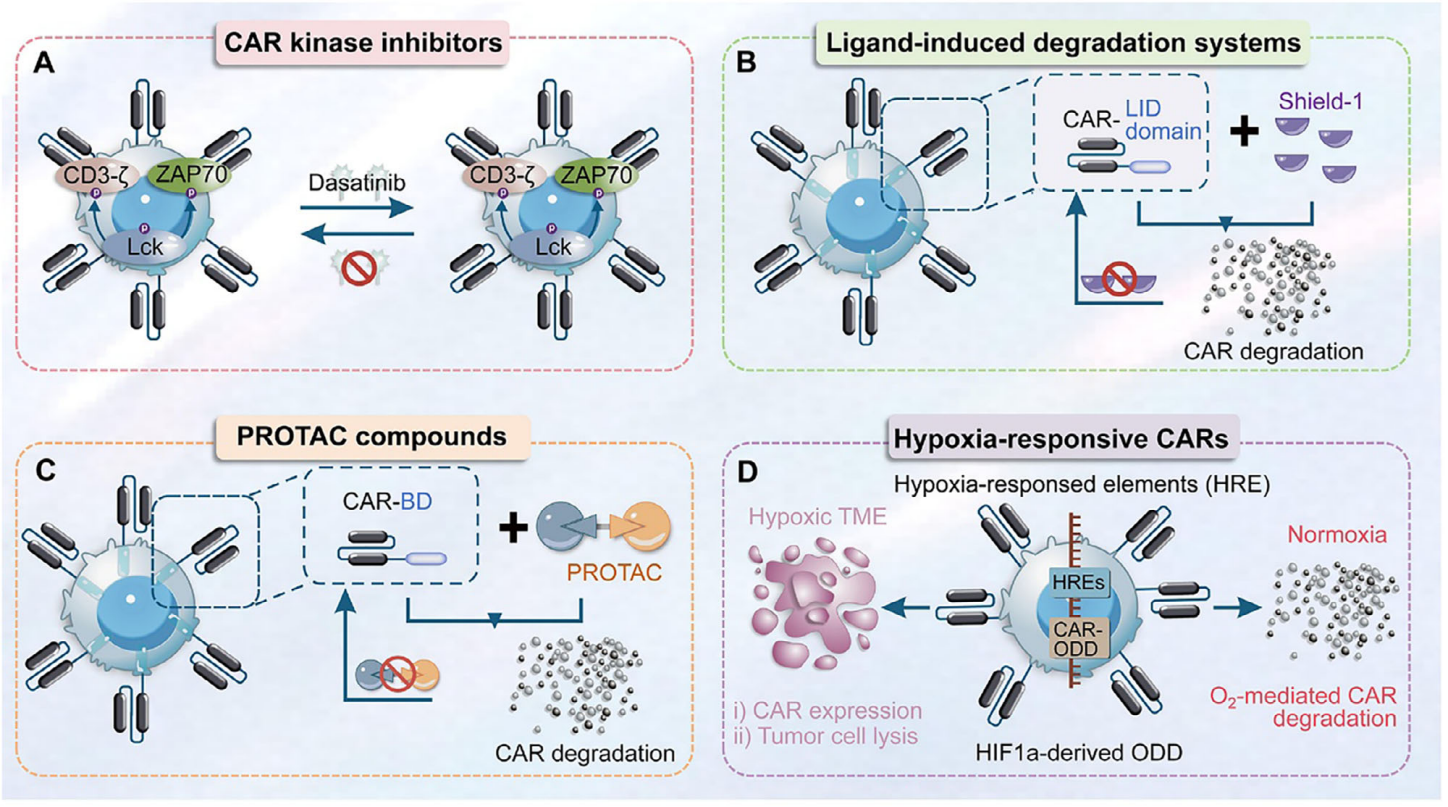
Reversible switching mechanisms in CARs provide a strategic alternative to the complete cessation of CAR‐T cell activity, primarily categorized into four distinct classes: (A) CAR kinase inhibitors, (B) ligand‐induced degradation systems, (C) PROTAC compounds, and (D) hypoxia‐responsive CARs.

In T‐cell therapy, suicide gene technology (facilitating engineered apoptosis induction) and antibody‐mediated T‐cell depletion (enabling cell surface antigen expression) have been implemented as “kill switches” to preempt adverse toxic reactions through irreversible cellular activity inhibition [201]. However, employing such kill switches to permanently eliminate CAR‐T cells from a patient's system may necessitate patient re‐infusion—a process that entails significant production costs and time expenditures. To advance clinical translation, researchers have engineered innovative methodologies for transiently inhibiting or degrading CAR proteins, thereby circumventing the necessity for repeated infusion procedures. These approaches encompass the administration of exogenous agents capable of temporarily suppressing CAR‐T cell functionality, including CAR kinase inhibitors, anti‐GD2 CAR‐T cells engineered with ligand‐induced degradation (LID) domains, proteolysis‐targeting chimera (PROTAC) compounds, and hypoxia‐sensing CARs. CAR‐T cell activity is subsequently reinstated upon agent withdrawal (Figure 8) [202]. Such reversible temporal inhibition strategies not only bolster temporal control but also effectively mitigate the risk of systemic CRS induction.

*CAR Kinases Inhibitors*: Dasatinib, a U.S. FDA‐approved tyrosine kinase inhibitor originally developed for chronic myeloid leukemia treatment, has been extensively investigated for its capacity to reversibly modulate CAR‐T cell activity as a strategy to mitigate CRS. Its mechanism of action involves inhibiting CAR‐T cell functionality through competitive ATP binding, specifically obstructing lymphocyte‐specific protein tyrosine kinase (Lck)‐mediated phosphorylation of ITAMs within T‐cell activation‐associated proteins, including the CD3‐ζ‐chain and ζ‐chain‐associated protein kinase 70 (ZAP70) [203]. This transient kinase blockade effectively suspends downstream signaling cascades while preserving cellular viability. Preclinical in vivo studies utilizing xenograft mouse models demonstrate that CAR‐T cell functional potency is fully restored within 24–48 h following cessation of dasatinib therapy, confirming the transient nature of its inhibitory effects through complete recovery of cytotoxic activity and cytokine production. A pioneering phase I dose‐escalation clinical trial (NCT04603872), employing a single‐arm open‐label design, is currently assessing the safety and efficacy of CD19‐targeted CAR‐T cells administered in conjunction with pulsed dasatinib therapy for treating relapsed/refractory MM, B‐cell ALL, and diffuse large B‐cell non‐Hodgkin's lymphoma [204]. Within this trial framework, dasatinib serves dual pharmacokinetic roles: as a pretreatment modulator administered 24 h prior to CAR‐T infusion to establish controllable activation dynamics, and as an on‐demand therapeutic intervention titrated through therapeutic drug monitoring to manage CRS severity (Grade ≥2) and neurotoxic adverse events while maintaining antitumor efficacy through intermittent dosing schedules.

*Ligand‐Induced Degradation*: Richman et al. designed an anti‐GD2 CAR‐T cell system incorporating a ligand‐induced degradation (LID) domain, originally engineered by Bonger et al. to enable reversible regulation of CAR protein expression. The mechanism centers on the interaction between the ligand Shield‐1 (or its water‐soluble derivative AquaShield, AS‐1) and mutant FKBP proteins [205]. Upon ligand binding, the degradation domain (degron) dissociates from its native binding site, triggering rapid proteasomal degradation of both the LID domain and its fused CAR construct. They have demonstrated that Shield‐1 administration substantially reduced CAR surface expression (by 85 ± 7% within 24 h) and IFN‐γ secretion (4.2‐fold decrease) in the CAR‐LID system, while moderate elution of Shield‐1 (0.5 µm AS‐1) restored CAR expression to near‐baseline levels. This study represents the inaugural demonstration of a ligand‐mediated regulatory system capable of precisely controlling CAR‐T cell expansion in vitro under antigenic stimulation (showing a 3.1‐fold proliferation difference between activated and suppressed states) and modulating antitumor efficacy in vivo (tumor volume reduction maintained at 63±9% in controlled groups vs 82±5% in continuous activation groups). Notably, the system maintained functional persistence through three on/off cycles without significant exhaustion markers, outperforming traditional transcriptional regulation approaches that typically permit only single ON/OFF transitions [206].

*PROTAC Compounds*: Lee et al. conceived a novel system leveraging PROTAC compounds to enable reversible regulation of CAR‐T cells through CAR protein degradation rather than genetic manipulation. This approach involved genetically fusing bromodomain (BD) tags to CAR proteins, followed by administering PROTAC compounds such as ARV825 and ARV771. These heterobifunctional molecules bridged BD‐tagged CAR proteins with E3 ubiquitin ligase complexes, initiating targeted ubiquitination and subsequent proteasomal degradation. In vitro experiments demonstrated dose‐responsive kinetics, with ARV771 achieving >90% CAR protein depletion within 6 h at 100 nM concentrations. The degradation directly correlated with functional attenuation, as evidenced by concentration‐dependent reductions in cytokine secretion (IL‐2 decreased from 4500 pg mL^−1^ to 300 pg mL^−1^) and tumor cell lysis efficacy (from 85% to 12% cytotoxicity). Crucially, upon removal of PROTAC compounds via medium exchange, the expression of the previously degraded CAR proteins was restored through de novo protein synthesis, with full functional recovery observed within 48 h. This reversible control mechanism maintained CAR‐T cell proliferative capacity across three treatment cycles, establishing a robust platform for precision immunotherapies requiring temporal modulation of cellular activity [207].

These novel regulatory mechanisms, founded upon analogous principles, enable precise modulation of CAR‐T cell activity and cytokine synthesis while circumventing the need for repeated therapeutic infusions. Nevertheless, their pronounced concentration‐dependent characteristics necessitate meticulous optimization of dosage parameters and delivery methodologies to maximize therapeutic efficacy while mitigating cytotoxic effects (e.g., PROTAC concentrations of 100–300 nM demonstrate a 20–30% reduction in CAR‐T cell viability) [208]. Advanced investigations into multidimensional control systems remain imperative to minimize iatrogenic cellular impairment and refine treatment outcomes. Reversible inhibition protocols emerge as a pivotal strategy for CRS prophylaxis, particularly as CAR‐T applications expand into solid tumor domains. This progression demands rigorous examination of therapeutic agent biodistribution, tumor microenvironment infiltration dynamics, and off‐target sequelae, potentially establishing a strategic paradigm for enhancing the safety profile of adoptive cell therapies [209].

*Hypoxia‐Sensing CARs*: To enhance the tumor microenvironment‐specific activity of CAR‐T cells while minimizing off‐tumor toxicity, researchers have developed hypoxia‐responsive regulatory strategies leveraging tumor‐specific metabolic features. The first‐generation approach integrates the oxygen‐dependent degradation (ODD) domain from HIF‐1α, a master regulator of cellular hypoxia responses, into CAR constructs [210]. This 54‐amino acid ODD module enables oxygen tension‐dependent proteasomal degradation through VHL‐mediated ubiquitination. Although CAR‐ODD T cells demonstrated 3.2‐fold increased tumor clearance in hypoxic tumor cores compared to conventional CAR‐T cells (*p* < 0.01), residual CAR activity persisted under normoxic conditions (21% baseline cytotoxicity in lung organoids). To achieve stricter spatial control, the second‐generation system combines post‐translational ODD regulation with hypoxia‐inducible transcriptional control. This dual‐lock mechanism incorporates both ODD‐mediated protein destabilization and HIF1α‐dependent CAR expression through synthetic promoters containing 8× hypoxia response elements (HREs). In orthotopic glioblastoma models, hypoxia‐induced CAR‐T cells showed complete target discrimination (0.3% vs 89% tumor cell lysis in normoxic vs hypoxic compartments) while maintaining potent tumor suppression (95.7% reduction in bioluminescent signal at day 14). Flow cytometry revealed 40‐fold higher CAR surface expression in tumor‐infiltrating lymphocytes compared to circulating counterparts. Notably, this oxygen‐sensing switch reduced serum IL‐6 levels by 83% and completely prevented cytokine release syndrome‐associated weight loss in NSG mice. Transcriptomic analysis of engineered T cells revealed downregulation of exhaustion markers (TIM‐3, LAG‐3) and enhanced metabolic fitness (increased PPAR‐γ signaling). Given that 92% of solid tumors exhibit oxygen gradients below 2% O_2_, this bioresponsive system addresses critical safety barriers for CAR‐T applications in epithelial cancers while preserving therapeutic potency through tumor‐selective activation.

## 7 Conclusions and Further Development

The development of CAR‐T cell therapy for solid tumors is continually refined to enhance efficacy, progressively advancing into the clinical stage. The inherent immune‐activating properties of cytokines provide a rationale for augmenting CAR‐T cell therapy. Through integrated design, cytokine‐engineered CAR‐T cells effectively potentiate T cell infiltration at tumor sites, counteract T cell exhaustion induced by the ITME, and foster the induction of long‐term immune memory, significantly improving patient survival. However, a recognized inherent limitation of conventional CAR‐T cell therapy is its propensity to induce severe oncologic toxicities in patients, notably CRS and ICANS, constraining its broader clinical application. Treatment necessitates administration within specialized medical institutions experienced in managing CRS and ICANS toxicities, imposing substantial financial and health burdens on patients. Concurrently, a deeper elucidation of the molecular and cellular pathophysiological mechanisms underpinning CRS and ICANS will facilitate the development of effective targeted therapies capable of mitigating toxicity without compromising antitumor activity. Current efforts in developing next‐generation CAR constructs focus on minimizing the risk of CRS and ICANS while optimizing tumor antigen recognition efficiency and T cell signaling potency. A critical additional bottleneck is the imperative to deepen our understanding of the biological characteristics and mechanisms of CAR T cells, particularly elucidating how the biophysical properties of the CAR molecule and its co‐stimulatory domains influence gene expression profiles, thereby modulating T cell subpopulation composition, functional status, memory potential, and susceptibility to exhaustion. To optimize therapeutic outcomes, there is an urgent need to cultivate T cell products exhibiting superior in vivo adaptability, durable persistence, and sustained effector function.

Current research endeavors prioritize the development of more precise CAR systems. This encompasses systems enabling the exogenous regulation of T cell function or survival. By modulating the affinity of single‐chain variable fragments (scFvs) and refining CAR architecture, the objective is to ameliorate toxicity and mitigate associated risks. Within preclinical models, investigators have explored diverse logic circuit strategies intrinsic to CAR proteins. These approaches aim to further constrain CAR T cell activation and their cytotoxic efficacy at tumor sites, concurrently controlling cytokine secretion levels. Furthermore, precise spatiotemporal regulation of CAR‐T–tumor cell contact, achieved through controlled cellular interaction dynamics, demonstrably reduces the risk of CRS; this methodology has garnered validation in early‐phase clinical trials. However, distinct from effector CAR T cell phenotypes, these regulatory CAR T cells manifest divergent in vivo biological characteristics and functionalities, potentially engendering novel toxicities. The capacity of engineered T cell therapy for safe, widespread application in both oncological and non‐malignant disease domains necessitates further empirical substantiation of its enduring safety and therapeutic efficacy.

The future advancement of cytokine‐engineered CAR‐T immunotherapy centers on achieving precise spatiotemporal control through molecular design and synthetic biology strategies. This approach aims to overcome the limitations and severe systemic toxicities inherent in traditional applications reliant on pharmacokinetic properties. First, protein engineering constructs “smart” cytokine variants exhibiting high receptor subtype selectivity, pH‐responsive dissociation, or protease‐conditional activation. This is achieved via rational design, including computational structural biology‐assisted interface optimization or directed evolution. These engineered molecules remain biologically inert during systemic circulation and are specifically activated only upon encountering specific stimuli within the ITME (e.g., elevated stromal metalloproteinase expression or mildly acidic pH conditions). Consequently, targeted release is achieved, significantly extending the therapeutic window and enhancing safety. Second, to systematically balance therapeutic efficacy and toxicity, current strategies extend beyond traditional delivery methods toward constructing dynamic gene circuits. By placing the cytokine expression cassette under the control of synthetic promoter systems (including tumor‐specific promoters, optogenetic regulatory elements, or exogenous small‐molecule induction systems) and integrating it into the genome of adoptive cell therapies (e.g., CAR‐T cells), a closed‐loop system with autonomous sensing‐feedback regulation capabilities is established. For instance, high‐potency cytokines such as IL‐12 are secreted locally only following CAR‐T cell recognition of tumor antigens, thereby fostering a self‐amplifying antitumor immune response. This enhances tumor clearance while effectively mitigating CRS. Critically, the most transformative paradigm shift involves the systematic integration of such controllable cytokine systems with off‐the‐shelf allogeneic cell therapy platforms. Utilizing high‐throughput gene editing tools like CRISPR‐Cas9 enables not only the knockout of endogenous TCR and MHC in host T cells to prevent GvHD and host immune rejection but also the simultaneous knockout of endogenous cytokine receptors. Based on this foundation, the introduction of orthogonal synthetic cytokine‐receptor pairs, such as rationally engineered IL‐2/IL‐2Rβγ systems designed to bind specifically to engineered receptors while avoiding cross‐reactivity with wild‐type counterparts, establishes a segregated “private communication” channel independent of the natural immune system. This channel allows exogenous supportive cytokines to selectively expand and maintain the infused off‐the‐shelf CAR‐T cells without activating host immune cells. Consequently, treatment persistence and efficacy are significantly enhanced while minimizing off‐target toxicity risks. Collectively, this synthetic immunology approach, integrating precision protein design, dynamic gene circuits, and advanced gene editing, signifies the transition of cytokine therapy from traditional, broad systemic delivery toward a next generation of highly intelligent, integrated, and unified “living” therapeutic systems.

## Conflicts of Interest

The authors declare no conflicts of interest.

## Data Availability

The authors have nothing to report.
